# Supplementary material for: Spatially targeted chemokine exocytosis guides transmigration at lymphatic endothelial multicellular junctions
Source: EMBO J. 2024 Jun 14;43(15):4. doi: 10.1038/s44318-024-00129-x (PMC11294460; doi:10.1038/s44318-024-00129-x)
Supplement: Supplementary file 1 — Appendix [file 44318_2024_129_MOESM1_ESM.pdf]

Appendix

Spatially targeted chemokine exocytosis guides transmigration at lymphatic endothelial multicellular junctions

Authors: Inam Liaqat, Ida Hilska, Maria Saario, Emma Jakobsson, Marko Crivaro, Johan Peränen, Kari Vaahtomeri*

*Correspondence to kari.vaahtomeri@helsinki.fi

Table of Contents:

Appendix Figure S1	p. 2
Appendix Figure S2	p. 3
Appendix Figure S3	p. 5
Appendix Figure S4	p. 6
Appendix Figure S5	p. 8
Appendix Figure S6	p. 12
Appendix Figure S7	p. 15
Appendix Figure S8	p. 17
Appendix Figure S9	p. 20
Appendix Figure S10	p. 23

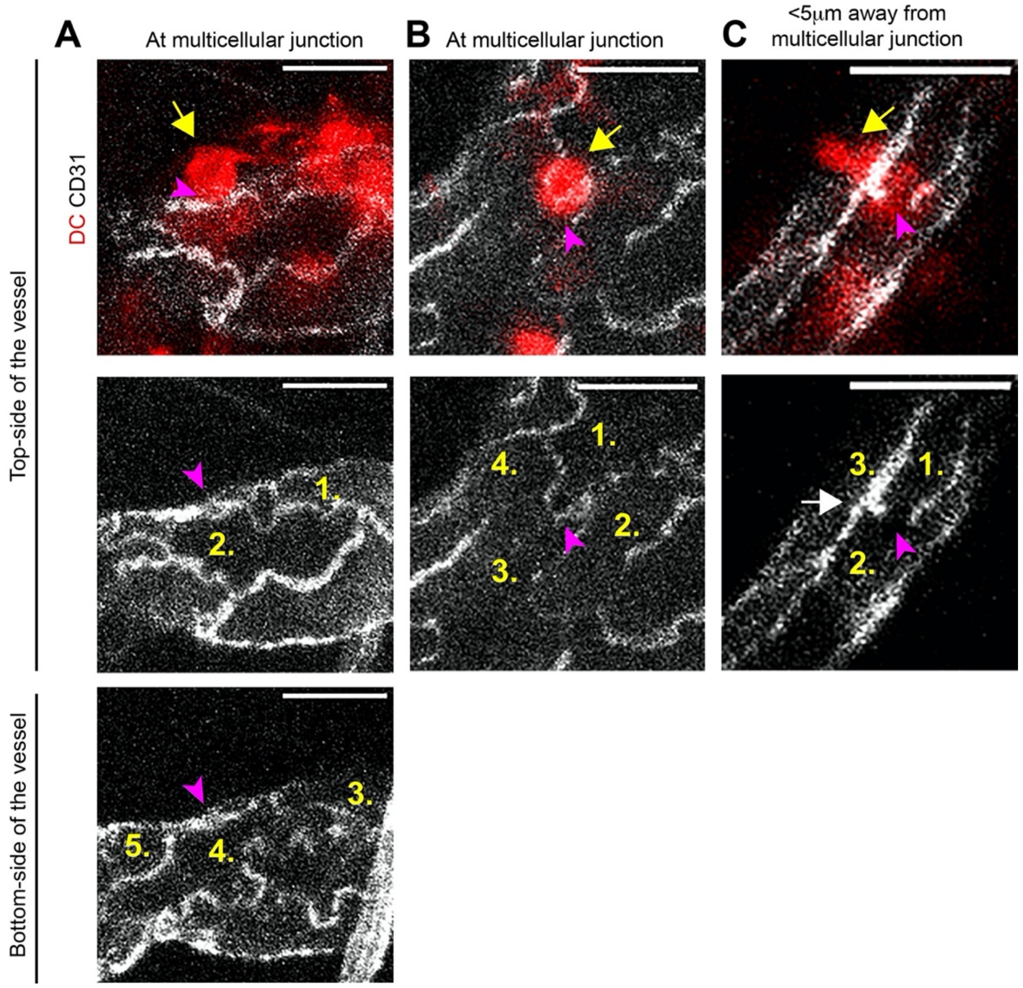

Appendix Figure S1: Multicellular junctions are the preferential DC transmigration sites in the dermal lymphatic vessels.

(A-C) Capture of a live recording shows a DC (red) transmigrating the α-CD31-FITC stained (gray) lymphatic endothelial junction in mouse ear pinna dermis explant. In (A) and (B) DC transmigrates a multicellular junction, and in (C) in the vicinity of the multicellular junction. The data represents (n=33 events) from 3 independent experiments and altogether 6 mice. The figure is related to Fig. 1D and Movies EV2-5.

Data information: The yellow arrows indicate the body of the transmigrating DC and the magenta arrowheads the site of the transmigration at the lymphatic endothelial junction. Scale bars are 20 μm.

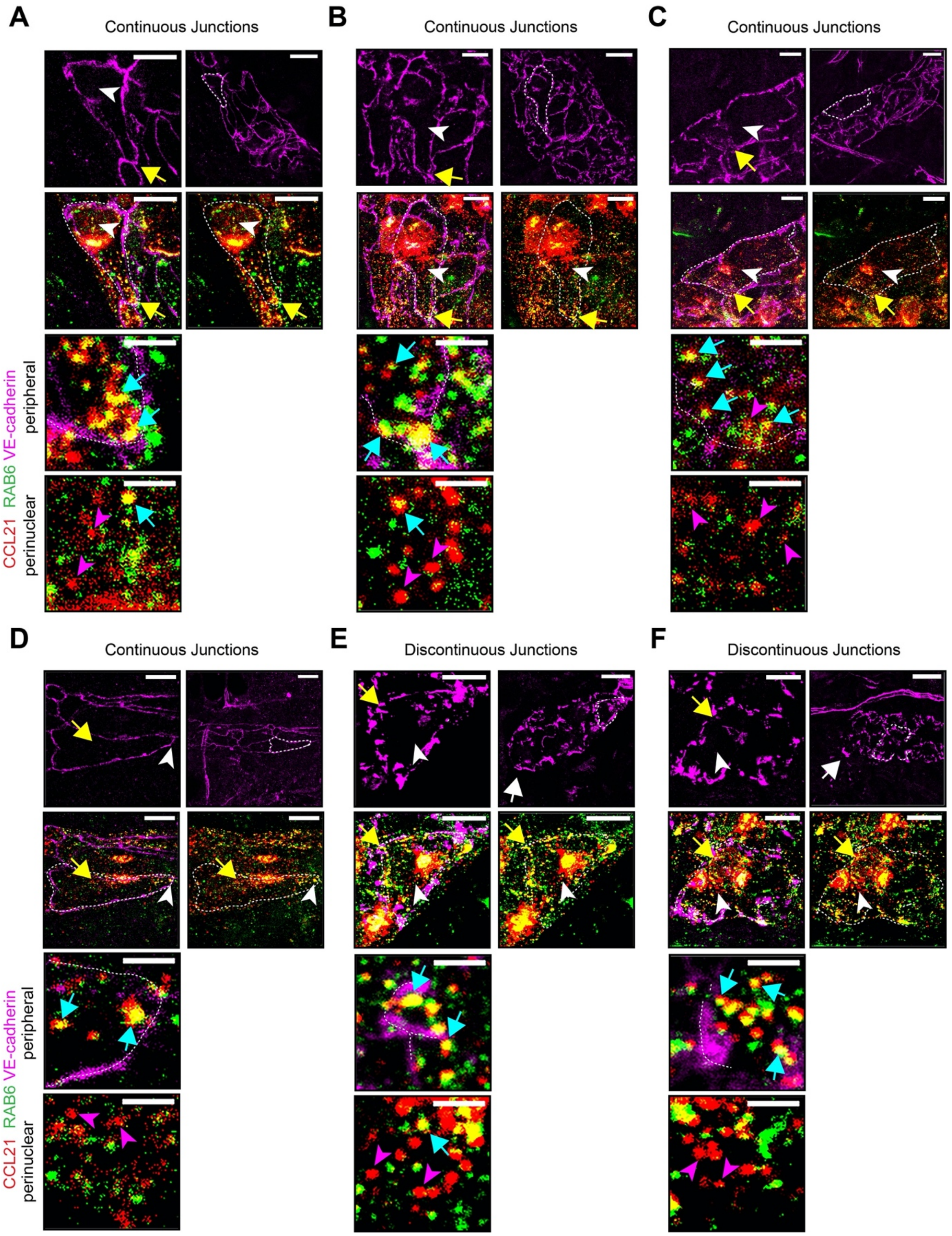
A
Continuous Junctions
B
Continuous Junctions
C
Continuous Junctions
CCL21 RAB6 VE-cadherin
peripheral
perinuclear
D
Continuous Junctions
E
Discontinuous Junctions
F
Discontinuous Junctions
CCL21 RAB6 VE-cadherin
peripheral
perinuclear

Appendix Figure S2: Colocalization of endogenous CCL21 and RAB6 in the dermal lymphatic vessels.

(A-F) Mouse-ear pinna dermis stained for CCL21 (red), RAB6 (green), and VE-cadherin (magenta). The top row of images shows the VE-cadherin-only channel of the analyzed LEC (left) and zoom-out image of part of the vessel (right) in each panel. The second row of images shows the same indicated LEC with all three channels or just CCL21 and RAB6. Panels (A-D) represent pre-collector LECs with continuous junctions, whereas (E-F) the blind-ended capillary LECs with discontinuous junctions. The images represent n=6 mice for continuous junctions (A-D) and n=3 mice for discontinuous junctions (E-F). The data is related to Fig. 2K-P. 4 out of 6 images shown in the Appendix Figures S2A and S2E are also shown in Fig. 2K and L, respectively, and with alternative colors (non-red and -green) in Appendix Fig S5K and S5L.

Data information: The cell borders are marked with white dotted lines. The yellow arrows indicate the peripheral, and the white arrowheads the perinuclear area shown in the zoom-in image. The cyan arrows indicate examples of CCL21 and RAB6 colocalization and the non-colocalized CCL21 vesicles are indicated with magenta arrowheads. The blind end of the capillaries in (E-F) are indicated with a white arrow in the overview image. Scale bars are 10 μm in overview images and 2 μm in the zoom-in images.

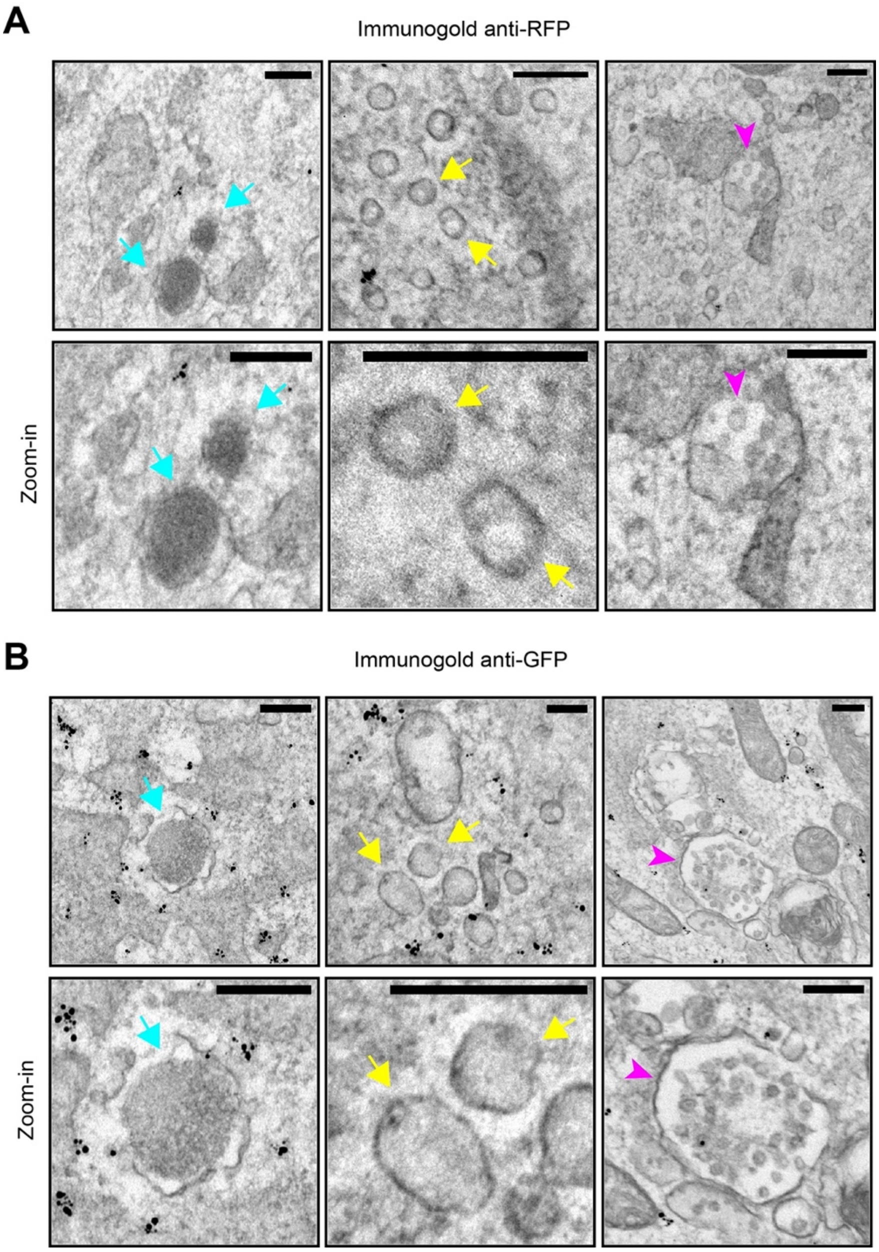

Appendix Figure S3: Transmission electron micrograph labelling controls

(A-B) Transmission electron micrographs of labelling controls: non-transduced LECs immunogold labelled with (A) α-RFP, or (B) α-GFP. Images are representatives of n=2 independent experiments.

Data information: Cyan arrows indicate dense core granules, yellow arrows low electron density vesicles, and magenta arrowheads the multivesicular bodies. Note the absence of

staining on the surface or inside of the vesicles. Scale bars are 200nm in overview and zoom-in images.

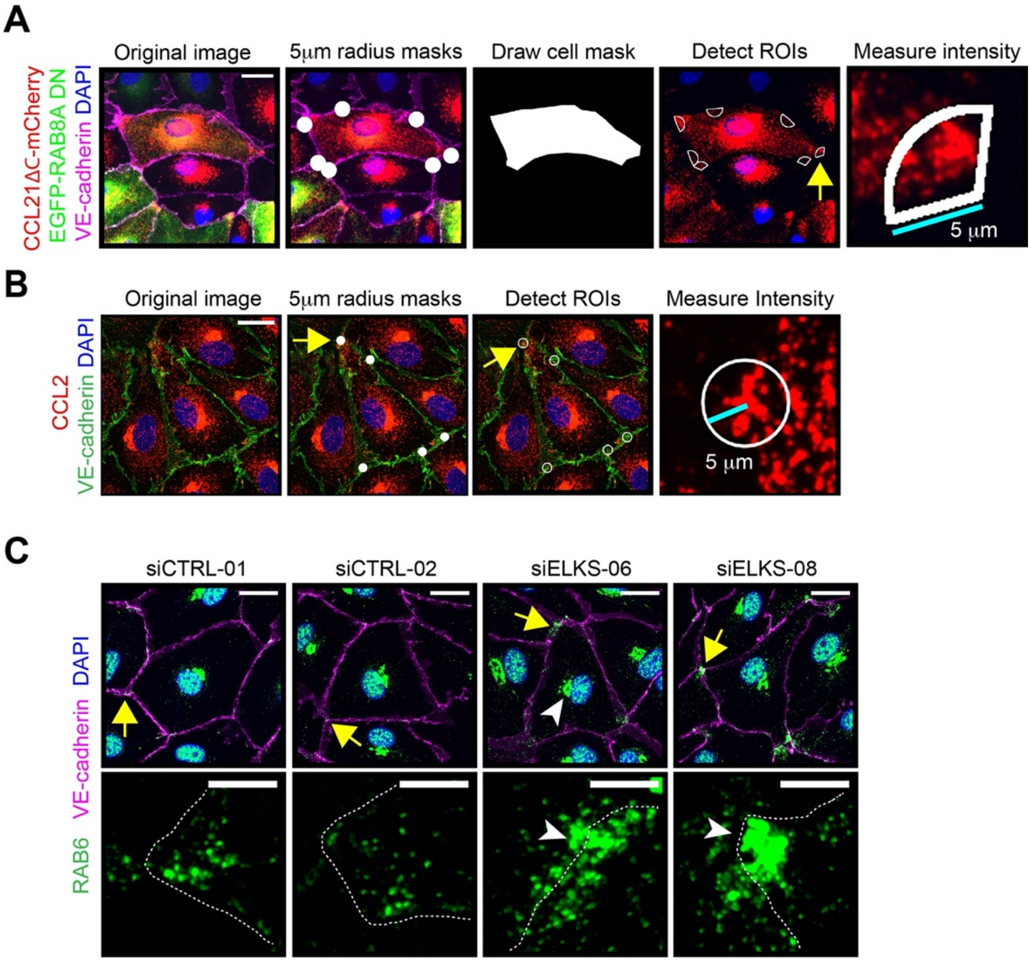

Appendix Figure S4: i) Method for quantification of the multicellular junction intensity of CCL21ΔC-mCherry and CCL2 and ii) multicellular junction accumulation of RAB6 upon ELKS silencing

(A-B) Immunofluorescence images showing the workflow for the quantification of the fluorescence intensity within a 5µm distance from multicellular junctions (see materials and methods). The workflow shown in (A) was applied to transduced LECs in quantifications related to Fig. 5E-G, 6C-E, and 7C-E; the shown example images are related to Fig. 7C-E: the LECs were expressing CCL21ΔC-mCherry (red) and EGFP-RAB8A DN (green) and stained

for VE-cadherin (magenta) and nuclei (DAPI, blue). The workflow shown in (B) was used for measuring CCL2 fluorescence intensity in siRNA-treated LECs in quantifications related to Fig. 6G-I, and 7H-J. Here, in the shown example images, the TNF-α-treated LECs are stained for CCL2 (red), VE-cadherin (green), and nuclei (DAPI, blue). The cell shown in "siELKS" image is also shown in original Fig. 6G (siELKS image) and with alternative colors (non-red and -green) in Appendix Fig S9. Here this same cell is used to explain the quantification method for the image analysis. **(C)** Images show the effect of siRNA-mediated silencing of ELKS on RAB6+ vesicles. The monolayers were treated with the indicated oligos and stained for RAB6 (green), VE-cadherin (magenta), and nuclei (DAPI, blue). The data represents n=4 biological replicates from four independent experiments.

Data information: In (A-C), the yellow arrows show the multicellular junction which is shown in the zoom-in image. In (C), white arrowheads indicate the accumulation of RAB6+ vesicles in siELKS-treated samples. Scale bars are 20μm in overview images and 5μm in zoom-in images.

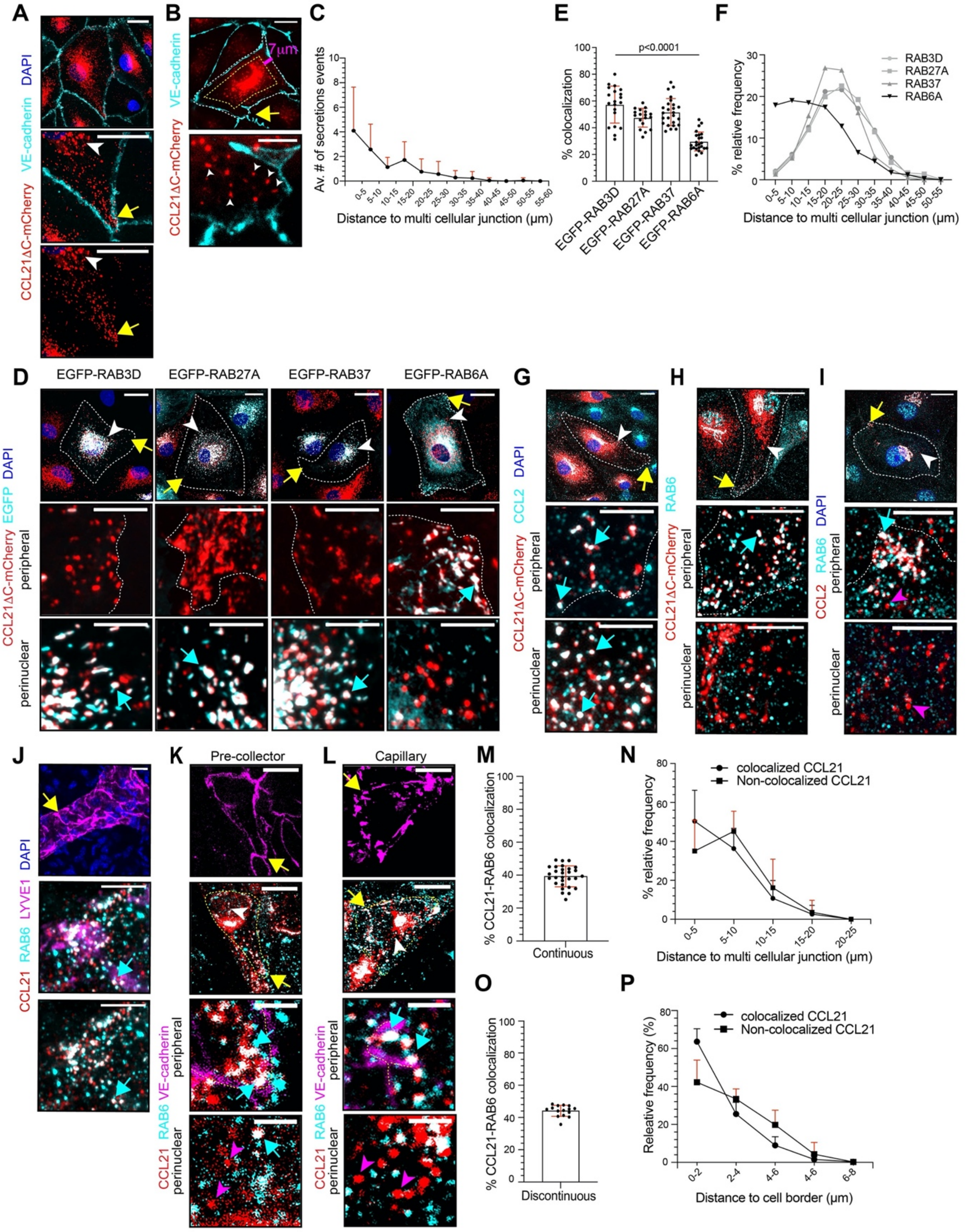

A
B
C
D
E
F
G
H
I
J
K
L
M
N
O
P
DAPI
VE-cadherin
CCL21ΔC-mCherry
7µm
Av. # of secretions events
Distance to multi cellular junction (µm)
% colocalization
p<0.0001
EGFP-RAB3D
EGFP-RAB27A
EGFP-RAB37
EGFP-RAB6A
% relative frequency
RAB3D
RAB27A
RAB37
RAB6A
CCL21ΔC-mCherry EGFP DAPI
peripheral
perinuclear
CCL21ΔC-mCherry CCL2 DAPI
CCL21ΔC-mCherry RAB6
CCL2 RAB6 DAPI
CCL21 RAB6 LYVE1 DAPI
Pre-collector
Capillary
CCL21 RAB6 VE-cadherin
% CCL21-RAB6 colocalization
Continuous
Discontinuous
colocalized CCL21
Non-colocalized CCL21
Releative frequency (%)
Distance to cell border (µm)

Appendix Figure S5: RAB-GTPase identity of chemokine CCL21 containing vesicles in LECs.

Original Fig. 2 is shown here, in Appendix Fig S5, with alternative colors. **(A)** LEC monolayer expressing CCL21ΔC-mCherry (red) and stained for VE-cadherin (cyan) and nuclei (DAPI, blue). The data in (A) represents at least n=3 independent experiments. **(B-C)** A capture of immunofluorescence live recording (Movie EV7) shows primary LEC monolayer expressing CCL21ΔC-mCherry (red) and stained for VE-cadherin (cyan). Exocytosis events were analyzed within a 7µm wide region. (C) A histogram showing exocytosis events (mean number or secretions/cell + SD) at the LEC junctions, as a function of distance (in µm) from the nearest multicellular junction. The data in (B and C) represents 241 secretion events from n=22 LECs in 5 biological replicates across two independent experiments. **(D-F)** LECs expressing chemokine CCL21ΔC-mCherry (red) and the indicated EGFP-tagged RAB-GTPase (cyan). The nuclei are stained with DAPI (blue). The images are representative of n=3 biological replicates in three independent experiments. Quantification in (E) shows percentage of CCL21ΔC-mCherry+ vesicle colocalization with the indicated EGFP-RAB GTPases in the whole LEC area. The dot plot shows the mean percentage ± SD. Each data point represents a single analyzed cell (EGFP-RAB3D (n=20); EGFP-RAB27A (n=18); EGFP-RAB37 (n=25) and EGFP-RAB6A (n=24)), from a total of 3 biological replicates in three independent experiments. The histogram (F) shows the distribution (mean percentage) of the colocalized vesicles as a function of distance from a multicellular junction. The number of samples was the same as in E. **(G)** A TNF-α-treated LEC, expressing chemokine CCL21ΔC-mCherry (red) and, stained for endogenous CCL2 (cyan), and nuclei (DAPI, blue). The images represent n=2 independent experiments. **(H)** A LEC expressing chemokine CCL21ΔC-mCherry (red) and stained for endogenous RAB6 (cyan). The images represent n=2 independent experiments. **(I)**

A TNFα-treated LEC monolayer stained for endogenous CCL2 (red), RAB6 (cyan), and nuclei (DAPI, blue). The images represent n=2 independent experiments. **(J)** Mouse-ear pinna dermis stained for CCL21 (red), RAB6 (cyan), LYVE1 (magenta), and nuclei (DAPI, blue). For clarity, the overview image shows only staining of LYVE1+ and nuclei. The images are representative of 3 mice. **(K)** Representative images show a mouse ear pinna dermis lymphatic pre-collector (continuous junctions, quantified in M-N) and **(L)** capillary (discontinuous junctions, quantified in O-P) stained for CCL21 (red), RAB6 (cyan), VE-cadherin (magenta). The overview images show VE-cadherin-only or CCL21 and RAB6. The zoom-in images show images of peripheral and perinuclear areas of the LEC. The Figure S5K and L are shown with more examples in Appendix Fig. S2A-F. **(M)** Quantification of CCL21 vesicle colocalization (mean percentage ± SD) with RAB6, in LECs showing continuous junctions in mouse ear pinna dermis *in vivo*. Each data point represents a single analyzed cell. **(N)** The histogram shows the distribution (mean percentage + SD) of CCL21 and RAB6 colocalized vesicles and non-colocalized CCL21 vesicles, as a function of distance from the multicellular junction (for LECs displaying continuous junctions). In (K) and (M-N), n=29 cells representing 6 mice. **(O)** Quantification of CCL21 vesicle colocalization (mean percentage ± SD) with RAB6, in LECs showing discontinuous junctions in mouse ear pinna dermis *in vivo*. Each data point represents a single analyzed cell. **(P)** The histogram shows the distribution (mean percentage + SD) of CCL21 and RAB6 colocalized vesicles and non-colocalized CCL21 vesicles, as a function of distance from the VE-cadherin stained cell border (for LECs displaying discontinuous junctions). In (L) and (O-P), n=15 cells representing 3 mice.

Data information: In (A), (D), (G-I) and (J-L) the yellow arrow indicates the peripheral, and the white arrowheads the perinuclear area shown in the zoom-in image. The cell borders in (D), (G-I) and (K-L) are marked with white dotted lines. The cyan arrow shows an example of colocalization and the magenta arrowheads examples of non-colocalizing vesicles. In (B), the

yellow arrow indicates LEC multicellular junction (shown in the zoom-in image and in Movie EV7). The vesicles that were exocytosed are marked with white arrowheads. The p-value in (E) was calculated using one way ANOVA test. Scale bars in (A-B), (D), and (G-J) are 20 µm in the overview images and 5 µm in the zoom-in images. In (K-L) the scale bars in overview images are 10 µm and 2 µm in the zoom-in images. Source data are available online for this figure.

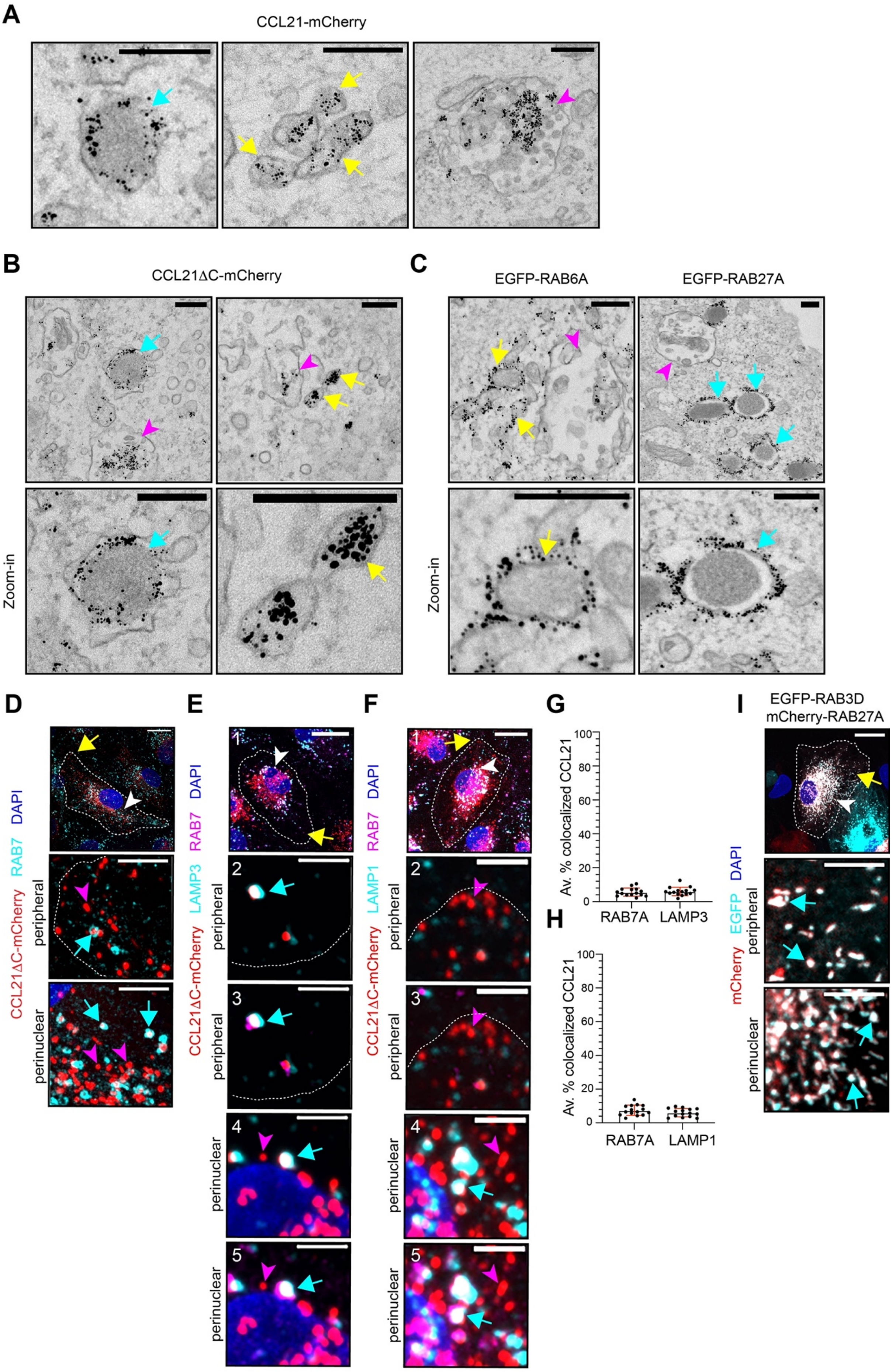
A
CCL21-mCherry
B
CCL21ΔC-mCherry
Zoom-in
C
EGFP-RAB6A
EGFP-RAB27A
Zoom-in
D
CCL21ΔC-mCherry RAB7 DAPI
peripheral
perinuclear
E
CCL21ΔC-mCherry LAMP3 RAB7 DAPI
peripheral
peripheral
perinuclear
perinuclear
F
CCL21ΔC-mCherry LAMP1 RAB7 DAPI
peripheral
peripheral
perinuclear
perinuclear
G
Av. % colocalized CCL21
RAB7A
LAMP3
H
Av. % colocalized CCL21
RAB7A
LAMP1
I
EGFP-RAB3D
mCherry-RAB27A
mCherry EGFP DAPI
peripheral
perinuclear

Appendix Figure S6: RAB27A-RAB3D vesicles represent dense-core secretory granules in LECs.

Original Fig. 3 is shown here, in Appendix Fig S6, with alternative colors. **(A-C)** Transmission electron micrograph of anti-RFP or anti-EGFP immunogold labeled (A) CCL21-mCherry, (B) CCL21ΔC-mCherry, (C) EGFP-RAB6A, or EGFP-RAB27A expressing LECs. The images represent n=2 independent experiments. Electron micrographs with control labelling are shown in Appendix Fig. S3A-B. **(D)** Shows a LEC expressing CCL21ΔC-mCherry (red) and stained for endogenous RAB7 (cyan). The images represent n=2 independent experiments. **(E)** LEC expressing CCL21ΔC-mCherry (red) and stained for endogenous LAMP3 (CD63; cyan) and RAB7 (magenta). The nuclei are stained with DAPI (blue). The zoom-in images, show either CCL21ΔC-mCherry and LAMP3 channels (images 2 and 4) or RAB7 together with CCL21ΔC-mCherry, and LAMP3 channels (images 3 and 5). The images represent n=3 independent experiments. Quantification shown in (G). **(F)** Shows LEC expressing CCL21ΔC-mCherry (red) and stained for endogenous LAMP1 (cyan), RAB7 (magenta) and nuclei (DAPI; blue). The zoom-in images, show either CCL21ΔC-mCherry and LAMP1 (images 2 and 4) or also with RAB7 (images 3 and 5). The images represent n=3 independent experiments. Quantification is shown in (H). **(G-H)** Quantification of CCL21ΔC-mCherry vesicle colocalization with endogenous RAB7A or LAMP3 in (G) and RAB7A or LAMP1 in (H). The dot plots show the mean percentage ± SD. Each data point represents a single analyzed cell with n=15. The data represents 5 biological replicates in three independent experiments. **(I)** Shows expression of EGFP-RAB3D (cyan) with mCherry-RAB27A (red) in TNF-α treated LECs. The images represent n=3 independent experiments.

Data information: In (A-C), cyan arrows indicate dense core granules, yellow arrows low-electron density vesicles, and magenta arrowheads multivesicular bodies. In (D-F) and (I), the

cell borders are marked with a white dotted line. Yellow arrows and white arrowheads indicate the site of the peripheral and perinuclear areas, respectively, shown in the zoom-in images. Cyan arrows indicate examples of CCL21ΔC-mCherry+ colocalizing vesicles and magenta arrowheads non-colocalizing CCL21ΔC-mCherry+ vesicles. Scale bars are 200 nm in transmission electron micrographs in (A-C); 20µm in overview image and 5µm in zoom-in images in (D-F) and (I). Source data are available online for this figure.

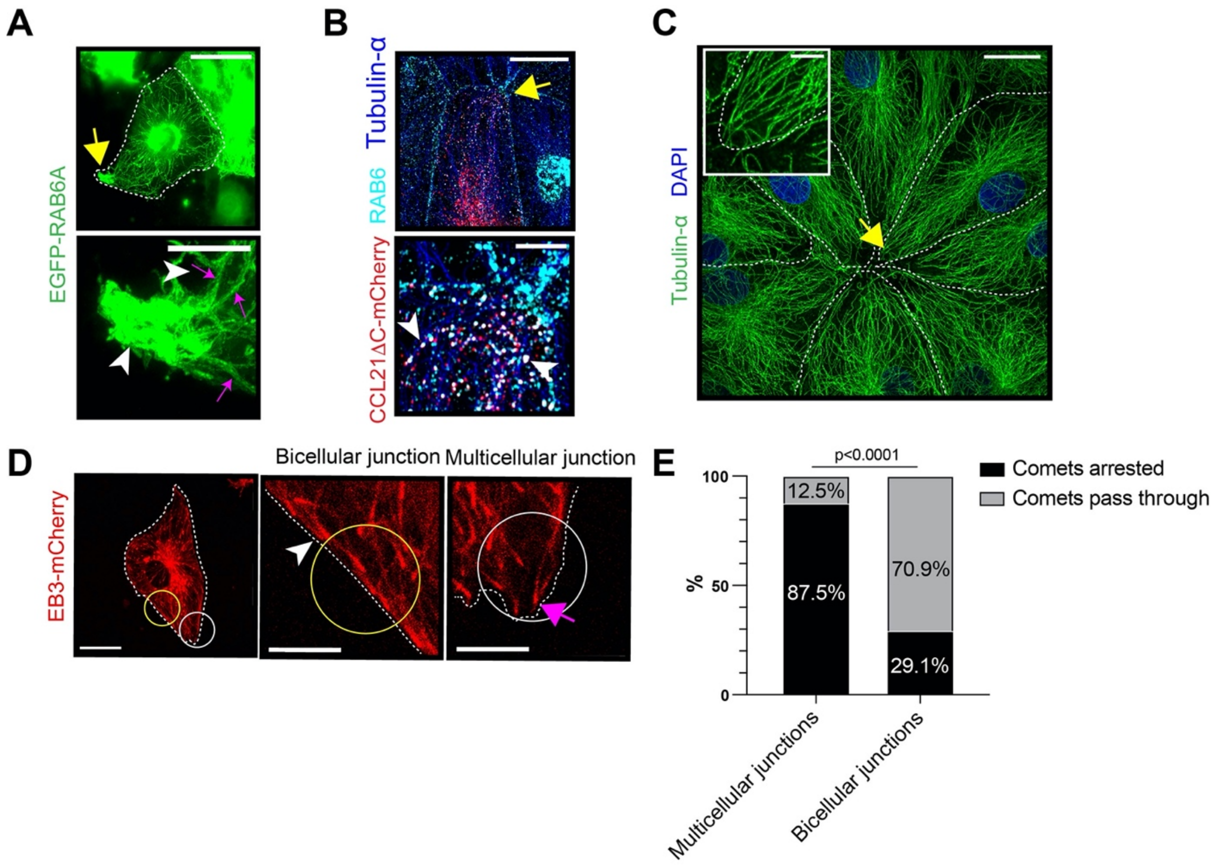

Appendix Figure S7: RAB6 tracks and microtubules target the LEC multicellular junctions.

Original Fig. 4 is shown here, in Appendix Fig S7, with alternative colors. **(A)** Maximum projection (in time) from a live recording of EGFP-RAB6A (green) expressing LEC monolayer. The images represent n=3 independent experiments. **(B)** The image shows a LEC monolayer expressing CCL21ΔC-mCherry (red) and stained for endogenous RAB6 (cyan) and tubulin-α (blue). The images represent n=2 independent experiments. (**C)** LEC monolayer stained for tubulin-α (green) and nuclei (DAPI, blue). The images represent n=3 independent experiments. **(D-E)** A capture of live recording of LEC monolayer expressing EB3-mCherry (Movie EV10). Yellow and white circles indicate examples of selected areas on bicellular and

multicellular junctions, respectively. These indicated areas were used for the quantification shown in (E). (E) A stacked bar graph shows mean percentage of EB3-mCherry comets that arrest or pass through the analyzed area. The data represents 21 cells (consisting of a total of 59 bicellular and 59 multicellular ROIs) from n=6 biological replicates in three independent experiments.

Data information: In (A), (C), and (D) white dotted line indicates LEC boundaries and in (A-C), the yellow arrow indicates the multicellular junction, which is shown in the zoom-in image. In (A), in the zoom-in images, magenta arrows indicate straight tracks of EGFP-RAB6A vesicles, whereas the white arrowheads indicate the dwelling of the EGFP-RAB6 vesicles at the multicellular junction of the LEC (see Movie EV9). In (B), white arrowheads indicate CCL21ΔC-mCherry+ RAB6 vesicles which are associated with microtubules. In (D), The white arrowhead indicates EB3-mCherry (red) positive comet, which will pass through the analyzed area (yellow circle). Whereas the magenta arrow indicates EB3-mCherry comet that arrests inside the white circle at the multicellular junction of the cell (see Movie EV10). In (E), the p-value was calculated using Fisher's exact test. The scale bar in (A) is 50µm in overview images and 10µm in zoom-in images and in (B-D), 20µm in overview images and 5µm in zoom-in images. Source data are available online for this figure.

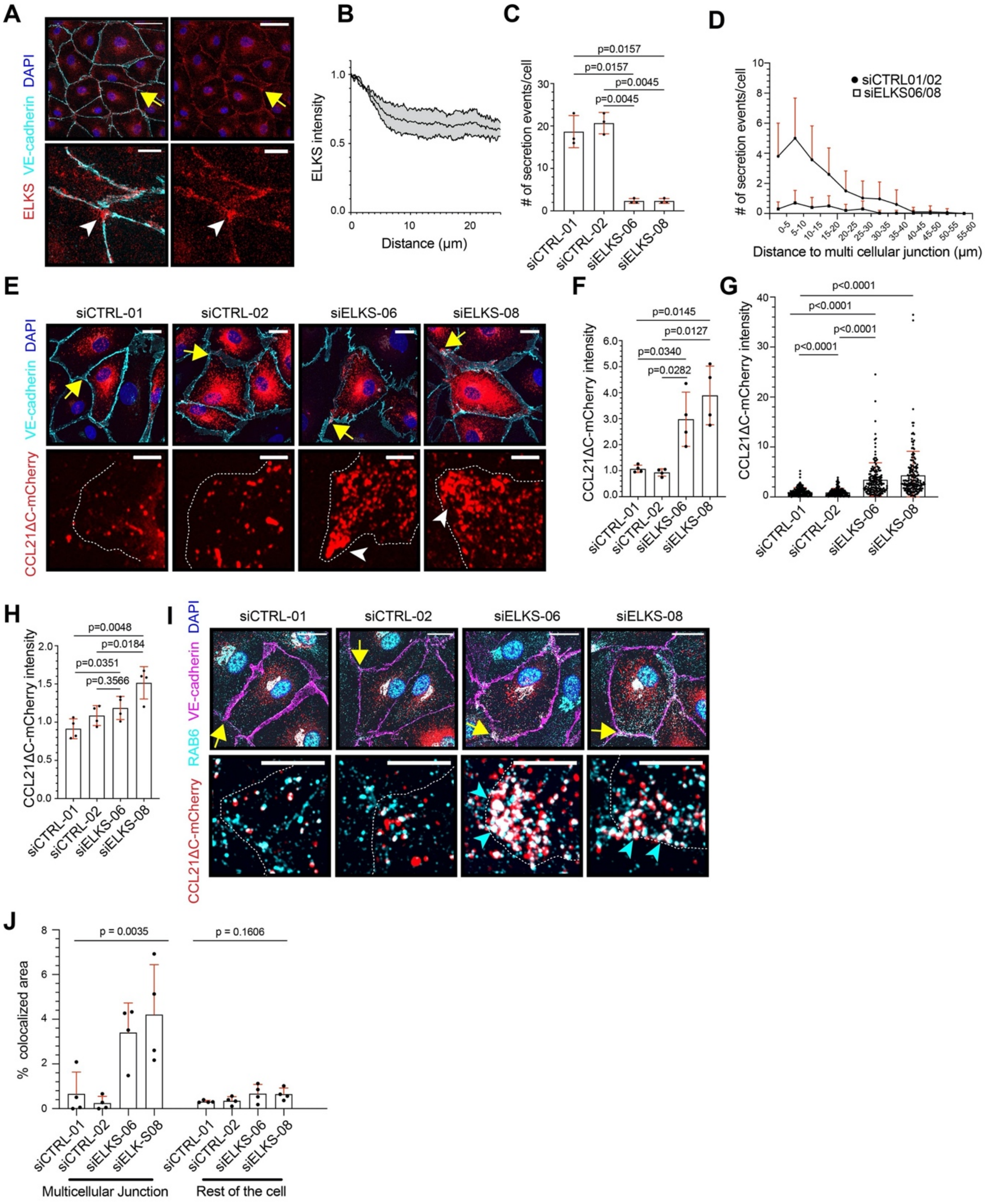

Appendix Figure S8: CCL21 is exocytosed at multicellular junctions in ELKS dependent manner.

Original Fig. 5 is shown here, in Appendix Fig S8, with alternative colors. **(A-B)** LEC monolayer stained for endogenous ELKS (red), VE-cadherin (cyan), and nuclei (DAPI, blue).

(B) A quantification of ELKS staining at the LEC junctions. The graph shows mean ELKS intensity ± SD as a function of distance from the nearest multicellular junction. In (A-B) the data represents a total of 250 junctions in 8 biological replicates and three independent experiments. **(C-D)** Quantification of the CCL21ΔC-mCherry exocytosis events at the LEC junctions in siControl and siELKS treated LEC monolayers. The dot plot in (C) shows a mean number of observed exocytosis events/cell ± SD. Whereas, in (D), the histogram shows the distribution of mean number of exocytosis events +SD, at the LEC junctions, as a function of distance from a multicellular junction. The data in (C-D) is derived from 3 independent experiments, representing, altogether, n=15 cells and 5 biological replicates per oligo (siCTRL01, siCTRL02, siELKS06, or siELKS08). In (D) the results from siCTRL01/02 or siELKS06/08 oligo transfected samples are pooled together. **(E-H)** Shows a LEC monolayer expressing CCL21ΔC-mCherry (red), treated with siControl or siELKS oligos and stained for VE-cadherin (cyan) and nuclei (DAPI, blue). The dot plots in (F-G) show the mean CCL21ΔC-mCherry intensity ± SD at the multicellular junction measured (F) per experiment and (G) per multicellular junction. Whereas the dot plot in (H) shows the mean CCL21ΔC-mCherry intensity ± SD measured in the whole LEC. Data points represent n=4 independent experiments or n=164 (siControl01), n=155 (siControl02), n=162 (siELKS06), and n=171 (siELKS08) multicellular junctions in (F-G) and 207 (siControl01), 225 (siControl02), 202 (siELKS06), and 216 (siELKS08) LECs in (H), from 6 biological replicates. In (F-H) the results were normalized to the average of controls (set at 1) in each experiment. **(I-J)** siControl and siELKS transfected LECs expressing CCL21ΔC-mCherry (red) and stained for endogenous RAB6 (cyan), VE-cadherin (magenta) and nuclei (DAPI, blue). (J) The dot plot shows the mean percentage colocalized area + SD of CCL21ΔC-mCherry and endogenous RAB6 in siControl or siELKS transfected LECs at the multicellular junctions (i.e., ≤5μm from the multicellular junction), or in the rest of the LEC. Each data point represents independent biological replicates

(n=4) from three experiments. Total number of analyzed multicellular junctions and LECs was 81 and 16, respectively, for siControl01, 91 and 18 for siControl02, 96 and 19 for siELKS06, and 92 and 19 for siELKS08.

Data information: In (A), (E), and (I) yellow arrows in the overview images indicate the multicellular junctions (shown as a zoom-in), and white arrowheads examples of accumulation in (A and E) and cyan arrowheads, the accumulation of colocalized vesicles in (I). In (E) and (I) white dotted lines mark the cell boundaries in the zoom-in images. In (C), (F) and (H) the p-values were calculated using a parametric T-test with Welch's correction, in (G) with a non-parametric Mann-Whitney's test, and in (J) with an ordinary ANOVA statistical test. The scale bar in (A) is 50µm in the overview images and 10µm in zoom-in images, and in (E and I) 20µm in overview images and 5µm in zoom-in images. Source data are available online for this figure.

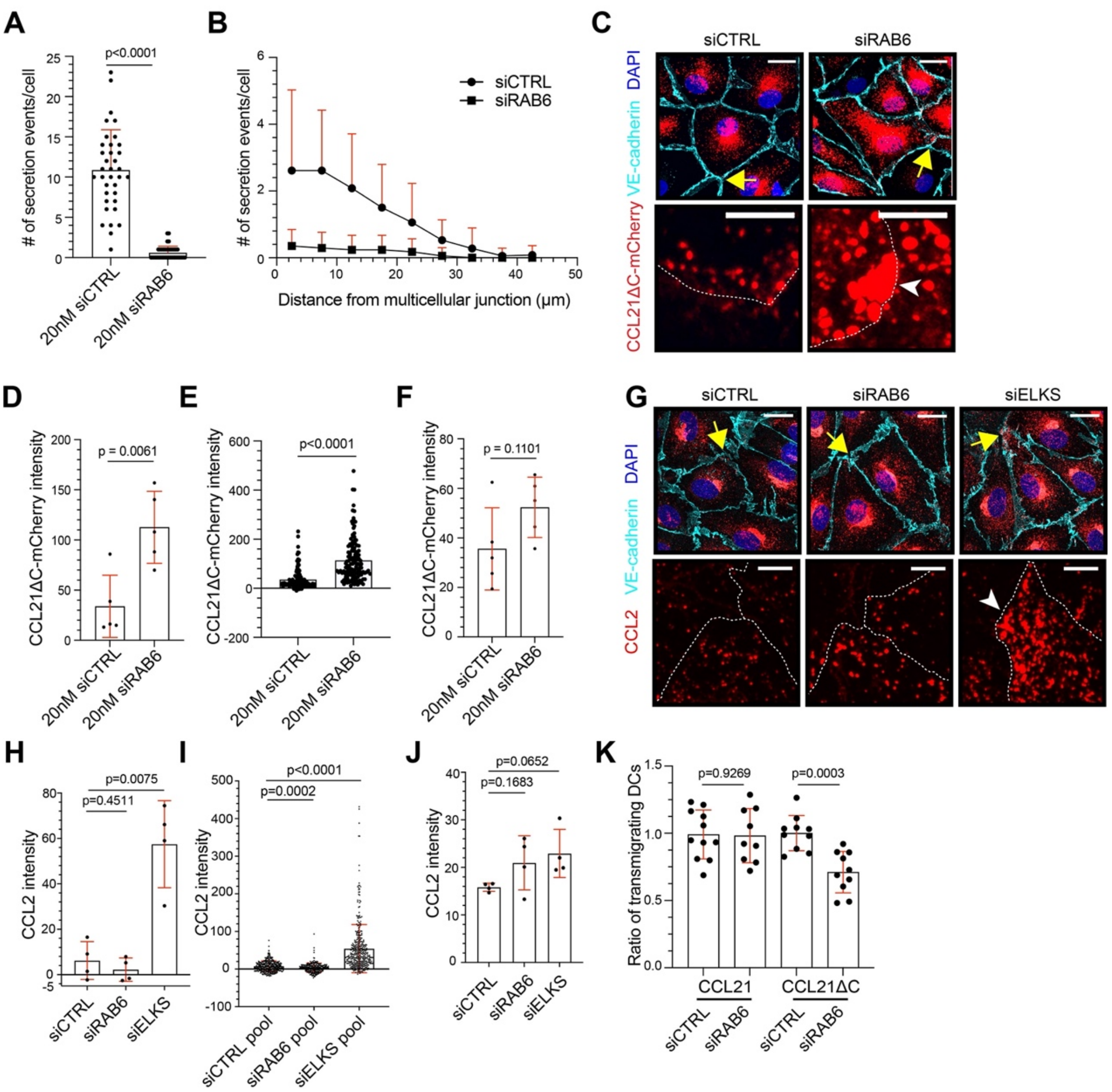

Appendix Figure S9: RAB6 is required for the CCL21 exocytosis at multicellular junctions

Original Fig. 6 is shown here, in Appendix Fig S9, with alternative colors. **(A-B)** The dot plot in (A), shows a mean number of observed exocytosis events/cell + SD. Whereas, in (B), the histogram shows the distribution of the mean number of exocytosis events/cell + SD at LEC junctions, as a function of distance from a multicellular junction. In (A-B), the data points represent n=39 cells in 13 biological replicates from three independent experiments. **(C-F)** Images of a LEC monolayer expressing CCL21ΔC-mCherry (red), treated with siControl or

siRAB6 oligos and stained for VE-cadherin (cyan) and nuclei (DAPI, blue). The dot plot in (D-E) shows the mean CCL21ΔC-mCherry intensity ± SD measured at the multicellular junctions per biological replicate (D) and (E) per multicellular junction, whereas the dot plot in (F) shows the mean CCL21ΔC-mCherry intensity ± SD measured in the whole LEC. Data points represent n=5 biological replicates or n=129 (siControl) and n=129 (siRAB6) multicellular junctions in (D-E) and 160 (siControl) and 164 (siRAB6) LECs in (F), altogether, in 4 independent experiments. **(G-J)** Images of TNF-α treated LEC monolayer transfected with siControl, siRAB6, or siELKS oligos, and stained for endogenous CCL2 (red), VE-cadherin (cyan), and nuclei (DAPI, blue). The dot plot shows the mean CCL2 intensity ± SD, measured at the multicellular junctions, (H) per experiment and (I) per multicellular junction. Whereas, in (J) the dot plot shows the mean CCL2 intensity ± SD, measured in the whole cell. Data points represents n=4 independent experiments or n=337 (siControl pool), n=268 (siRAB6 pool), and n=384 (siELKS pool) multicellular junctions from 5 (siControl pool and siRAB6 pool) or 6 (siELKS pool) biological replicates in (H-I), and 37 (siControl pool and siRAB6 pool), and 42 (siELKS pool) LECs from 6 (siControl pool and siRAB6 pool) or 7 (siELKS pool) biological replicates in (J). **(K)** Quantification of the mean DC transmigration efficiency ± SD on LEC monolayer transfected with siControl or siRAB6 and transduced with CCL21-mCherry or CCL21 ΔC-mCherry. The results were normalized to the average of control samples (set at 1) in each experiment. The data points represent biological replicates: CCL21-mCherry + siControl n=11 (4359 DCs), CCL21-mCherry + siRAB6 n=9 (4091 DCs), CCL21ΔC-mCherry + siControl n=10 (3319 DCs), CCL21ΔC-mCherry + siRAB6 n=10 (3766 DCs), across three independent experiments. The data is related to Movies EV11 and EV12.

Data information: In (C) and (G) yellow arrows indicate the multicellular junction (area shown in the zoom-in images below), and white arrowheads indicate the accumulation. White dotted lines represent the cell borders. In (A), (D), (F), (H), (J), and (K), the p-values were calculated

using a parametric T-test with Welch's correction and in (E) and (I) the p-values were calculated using Mann-Whitney's test. The scale bar in (C) and (G) is 20µm in overview images and 5µm in zoom-in images. Source data are available online for this figure.

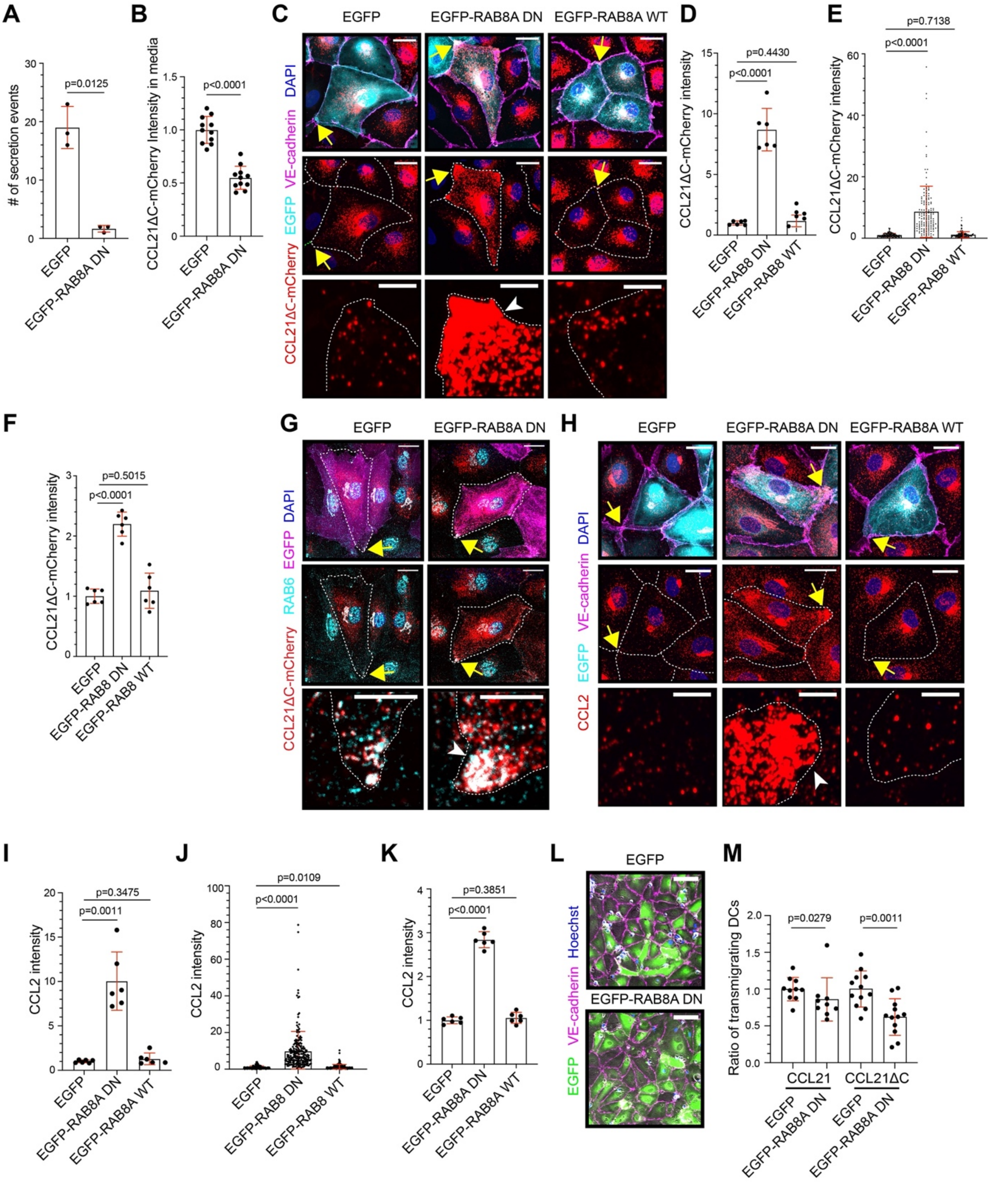

Appendix Figure S10: Dominant negative RAB8A inhibits CCL21 exocytosis and DC transmigration.

Original Fig. 7 is shown here, in Appendix Fig S10, with alternative colors. **(A)** Quantification of the CCL21ΔC-mCherry exocytosis events at the LEC junctions in EGFP or EGFP-RAB8A

DN expressing LECs. The dot plot shows the mean number of exocytosis events/cell ± SD. Data points represents n=3 independent experiments, comprising of, altogether, 24 LECs in 12 biological replicates. **(B)** Quantification of the effect of EGFP-RAB8A DN expression on CCL21ΔC-mCherry intensity in the media. The dot plot shows CCL21ΔC-mCherry mean intensity ± SD, and normalized to the average of controls, which was set at 1 in each experiment. The data points represent n=11 biological replicates in three independent experiments. **(C-F)** Shows a LEC monolayer expressing CCL21ΔC-mCherry (red) and EGFP, EGFP-RAB8A DN, or EGFP-RAB8A WT (cyan). LECs were stained for VE-cadherin (magenta) and nuclei (DAPI, blue). Quantification of the mean CCL21ΔC-mCherry intensity ± SD at the multicellular junctions of EGFP-mCherry double-positive LECs (D) per biological replicate and (E) per multicellular junction. Whereas the dot plot in (F) shows the mean CCL21ΔC-mCherry intensity ± SD measured in the whole LEC. The results were normalized to the average of controls, which was set at 1, in each experiment. The data points represent n=6 biological replicates in three independent experiments or n=149 (EGFP), n=153 (EGFP-RAB8A DN), and n=147 (EGFP-RAB8 WT) multicellular junctions in (D-E) and 212 (EGFP), 188 (EGFP-RAB8A DN), and 170 (EGFP-RAB8 WT) LECs in (F). **(G)** Immunofluorescence images of a LEC monolayer expressing CCL21ΔC-mCherry (red) and either EGFP or EGFP-RAB8A DN (magenta). The cells were stained for endogenous RAB6 (cyan), and nuclei (DAPI, blue). The data represents n=4 biological replicates from 2 independent experiments. **(H-K)** Shows TNF-α treated LEC monolayer expressing EGFP (control), EGFP-RAB8A DN, or EGFP-RAB8A WT (cyan). LECs were stained for CCL2 (red), VE-cadherin (magenta), and DAPI (blue). Quantification of the mean CCL2 intensity ± SD at the multicellular junctions (I) per biological replicate and (J) per multicellular junction. Whereas the dot plot in (K) shows mean CCL2 intensity ± SD measured in the whole LEC. The data was normalized to the average of controls (set at 1) in each experiment. The data points represent n=6 biological

replicates from 3 independent experiments or n=204 (EGFP), n=202 (EGFP-RAB8A DN), and n=192 (EGFP-RAB8 WT) multicellular junctions in (I-J), and 242 (EGFP), 240 (EGFP-RAB8A DN), and 136 (EGFP-RAB8 WT) LECs in (K). **(L-M)** A capture of live recording of LEC monolayer expressing CCL21-mCherry and either of EGFP or EGFP-RAB8A DN (green) and stained for VE-cadherin (magenta). The nuclei of dendritic cells (DC) are stained with Hoechst (blue). (M) Quantification of the mean DC transmigration efficiency ± SD on LEC monolayer co-expressing either EGFP (control) or EGFP-RAB8A DN together with either CCL21-mCherry or CCL21 ΔC-mCherry. The results were normalized to the average of control (set at 1) in each experiment. The data points represent n=10 CCL21-mCherry + EGFP (total of 3285 DCs), n=9 CCL21-mCherry + EGFP-RAB8A DN (2552 DCs), n=12 CCL21 ΔC-mCherry + EGFP (7123 DCs), and n=12 CCL21 ΔC-mCherry + EGFP-RAB8A DN (5535 DCs) biological replicates, across 4 independent experiments. The data in (L-M) is related to the Movie EV13.

Data information: In mCherry channel-only images in (C), the cell borders of all the EGFP-mCherry, double-positive LECs, are indicated with white dashed lines. Similarly, in (H) the white dashed lines indicate the cell borders of all EGFP-CCL2 double positive LECs. Cell borders in (G) are shown with white dashed line. In (C), (G), and (H), yellow arrows indicate the multicellular junction shown in the zoom-in images and the white arrowheads the accumulation. In (A-B), (D), (F), (I), (K), (M, CCL21 ΔC-mCherry samples), the p-values were calculated using a parametric T-test with Welch's correction, and in (E), (J), and (M, CCL21-mCherry samples) using Mann-Whitney's test. Scale bars in (C, G and H) are 20µm in overview images and 5µm in zoom-in images and in (L) 50µm.
