# Supplementary material for: Spatially targeted chemokine exocytosis guides transmigration at lymphatic endothelial multicellular junctions
Source: EMBO J. 2024 Jun 14;43(15):4. doi: 10.1038/s44318-024-00129-x (PMC11294460; doi:10.1038/s44318-024-00129-x)
Supplement: Supplementary file 3 — Movie EV1 [file 44318_2024_129_MOESM3_ESM.zip › Movie EV1/readme Movie EV1.rtf]

Movie EV1. Phase contrast and epifluorescence microscopy recording of a DC transmigrating across a multicellular junction (yellow arrow) of CCL21-mCherry expressing LEC monolayer. LEC junctions (magenta) were stained with a non-blocking VE-cadherin antibody. Frame interval is 90’’and scale bar 20µm. The time stamp shows minutes. The movie is related to Fig. 1A. The movie represents 8 biological replicates in three independent experiments. n=361 transmigration events. See quantification in Fig. 1B.
