# Supplementary material for: Spatially targeted chemokine exocytosis guides transmigration at lymphatic endothelial multicellular junctions
Source: EMBO J. 2024 Jun 14;43(15):4. doi: 10.1038/s44318-024-00129-x (PMC11294460; doi:10.1038/s44318-024-00129-x)
Supplement: Supplementary file 4 — Movie EV3 [file 44318_2024_129_MOESM4_ESM.zip › readme Movie EV3.rtf]

Movie EV3. Immunofluorescence confocal microscopy recording of a labeled DC (red) transmigrating across the anti-CD31-stained (grey) lymphatic endothelial junctions into a lymphatic vessel, in mouse ear explant. On the right-hand side, the CD31 channel-only is shown, and the magenta arrowhead indicates the site of transmigration. The movie shows a single Z-layer around the plane of lymphatic endothelium. The frame interval is 40’’ and scale bar 10µm. The time stamp shows minutes and seconds. See Appendix Fig. S1A for the annotation of the LECs contributing to the multicellular junction both on the top and bottom side of the vessel. Movies EV2-5 represent n=33 transmigration events in explants derived from 6 mice, altogether, in three independent experiments. The movie is related to Appendix Fig. S1A. See Fig. 1D for the quantification.
