# Supplementary material for: Spatially targeted chemokine exocytosis guides transmigration at lymphatic endothelial multicellular junctions
Source: EMBO J. 2024 Jun 14;43(15):4. doi: 10.1038/s44318-024-00129-x (PMC11294460; doi:10.1038/s44318-024-00129-x)
Supplement: Supplementary file 5 — Movie EV2 [file 44318_2024_129_MOESM5_ESM.zip › readme Movie EV2.rtf]

Movie EV2. Immunofluorescence confocal microscopy recording of a labeled DC (red) transmigrating across the anti-CD31-stained (grey) lymphatic endothelial junctions into a lymphatic vessel, in mouse ear explant. On the right-hand side, the CD31 channel-only is shown, and the magenta arrowhead indicates the site of transmigration. The movie shows the max-projection of 3 Z-layers around the plane of lymphatic endothelium. The frame interval is 54’’ and the scale bar is 10µm. The time stamp shows minutes and seconds. See Fig. 1C for the annotation of the LECs contributing to the multicellular junction. Movies EV2-5 represent n=33 transmigration events in explants derived from 6 mice, altogether, in three independent experiments. The movie is related to Fig. 1C. See Fig. 1D for the quantification.
