# Supplementary material for: Spatially targeted chemokine exocytosis guides transmigration at lymphatic endothelial multicellular junctions
Source: EMBO J. 2024 Jun 14;43(15):4. doi: 10.1038/s44318-024-00129-x (PMC11294460; doi:10.1038/s44318-024-00129-x)
Supplement: Supplementary file 7 — Movie EV4 [file 44318_2024_129_MOESM7_ESM.zip › readme Movie EV4.rtf]

Movie EV4. Immunofluorescence confocal microscopy recording of a labeled DC (red) transmigrating across the anti-CD31-stained (grey) lymphatic endothelial junctions into a lymphatic vessel, in mouse ear explant. The middle movie shows the CD31 channel-only and the magenta arrowhead indicates the site of transmigration. The movie on the left and in the middle are max-projection of 4 Z-layers around the plane of lymphatic endothelium. The movie on the right-hand side represents a single optical slice of the lymphatic vessel lumen and shows the DCs entering into the lumen of the lymphatic vessel. The frame interval in the movie is 40’’ and the scale bar is 10µm. The time stamp shows minutes and seconds. See Appendix Fig. S1B for the annotation of the LECs contributing to the multicellular junction. Movies EV2-5 represent n=33 transmigration events in explants derived from 6 mice, altogether, in three independent experiments. The movie is related to Appendix Fig. S1B. See Fig. 1D for the quantification.
