# Supplementary material for: Spatially targeted chemokine exocytosis guides transmigration at lymphatic endothelial multicellular junctions
Source: EMBO J. 2024 Jun 14;43(15):4. doi: 10.1038/s44318-024-00129-x (PMC11294460; doi:10.1038/s44318-024-00129-x)
Supplement: Supplementary file 8 — Movie EV6 [file 44318_2024_129_MOESM8_ESM.zip › Movie EV6/readme Movie EV6.rtf]

Movie EV6. Phase contrast and epifluorescence microscopy recording of wild-type (left panel) or CCR7-/- DC (right panel), migrating on a CCL21-mCherry expressing LEC monolayer, followed by an arrest (yellow arrowheads). Note that the wild-type DC repolarizes towards the LECs at multicellular junctions. However, CCR7-/- DC does not polarize towards the monolayer and, eventually, continues to travel past the LEC multicellular junctions. LEC junctions (magenta) were stained with a non-blocking VE-cadherin antibody. The frame interval is 90’’ and the scale bar is 20µm. The time stamp shows minutes. The movie represents n=6 wild-type and n=5 CCR7-/- biological replicates and, altogether, three independent experiments. The movie is related to Fig. 1E-G. See Fig. 1F-G for quantification.
