# Supplementary material for: Spatially targeted chemokine exocytosis guides transmigration at lymphatic endothelial multicellular junctions
Source: EMBO J. 2024 Jun 14;43(15):4. doi: 10.1038/s44318-024-00129-x (PMC11294460; doi:10.1038/s44318-024-00129-x)
Supplement: Supplementary file 9 — Movie EV7 [file 44318_2024_129_MOESM9_ESM.zip › Movie EV7/readme Movie EV7.rtf]

Movie EV7. Epifluorescence microscopy recording of CCL21 deltaC-mCherry (red) expressing LEC monolayer. The white line indicates the LEC junctions. CCL21 deltaC-mCherry exocytosis events at multicellular junctions were detected by the sudden disappearance of vesicles (marked with white arrowheads). The frame interval is 400ms and the scale bar is 3µm. The time stamp shows seconds. The movie is related to Fig. 2B-C. The movie represents n=5 biological replicates across two independent experiments. See Fig. 2C for quantification.
