# Supplementary material for: Spatially targeted chemokine exocytosis guides transmigration at lymphatic endothelial multicellular junctions
Source: EMBO J. 2024 Jun 14;43(15):4. doi: 10.1038/s44318-024-00129-x (PMC11294460; doi:10.1038/s44318-024-00129-x)
Supplement: Supplementary file 10 — Movie EV8 [file 44318_2024_129_MOESM10_ESM.zip › readme Movie EV8.rtf]

Movie EV8. Epifluorescence microscopy recording of LEC monolayer expressing CCL21 deltaC-mCherry. The first frame of the movie shows cell junctions stained with non-blocking VE-cadherin antibody (magenta) and a phase contrast channel showing a DC arrested on top of a multicellular junction (right panel) and absence of DC in the left panel. CCL21 deltaC-mCherry exocytosis events at multicellular junctions were detected by the sudden disappearance of vesicles (marked with white arrowheads). Note that DC at multicellular junction does not induce CCL21 deltaC-mCherry exocytosis. The frame interval is 400ms and the scale bar is 10µm. Time stamp shows seconds. For the control movie n=15 biological replicates and for “with arrested DC” n=12 biological replicates, both, in 2 experiments.
