# Supplementary material for: Spatially targeted chemokine exocytosis guides transmigration at lymphatic endothelial multicellular junctions
Source: EMBO J. 2024 Jun 14;43(15):4. doi: 10.1038/s44318-024-00129-x (PMC11294460; doi:10.1038/s44318-024-00129-x)
Supplement: Supplementary file 11 — Movie EV9 [file 44318_2024_129_MOESM11_ESM.zip › Movie EV9/readme Movie EV9.rtf]

Movie EV9. Epifluorescence microscopy recording of EGFP-RAB6A (green) expressing LEC monolayer. Only the transduced LEC is seen in the movie. The white line indicates the LEC junctions. EGFP-RAB6+ vesicles were directionally trafficked in and out of the multicellular junction, whereas, at the multicellular junction, EGFP-RAB6 vesicles show non-directional movement. The frame interval is 400ms and the scale bar 5µm. The time stamp shows seconds. The movie is related to Fig. 4A. The movie represents n=3 independent experiments.
