# Supplementary material for: Spatially targeted chemokine exocytosis guides transmigration at lymphatic endothelial multicellular junctions
Source: EMBO J. 2024 Jun 14;43(15):4. doi: 10.1038/s44318-024-00129-x (PMC11294460; doi:10.1038/s44318-024-00129-x)
Supplement: Supplementary file 12 — Movie EV10 [file 44318_2024_129_MOESM12_ESM.zip › Movie EV10/readme Movie EV10.rtf]

Movie EV10. Spinning disc confocal microscopy recording of a LEC monolayer expressing EB3-mCherry (red). Only the transduced LEC is seen in the movie. EB3-mCherry comets indicate the plus end of growing microtubules. Yellow and white circles indicate the areas used for analyses of comet movement at the bi- and multicellular junctions, respectively. The movie is a max projection of 5 Z-layers. The frame interval is 638 ms and the scale bar is 20µm. The time stamp shows seconds. The movie is related to Fig. 4D-E. The movie represents n = 21 LECs (consisting of a total of 59 bicellular and 59 multicellular ROIs) from 6 biological replicates in three independent experiments. The quantification is shown in Fig. 4E.
