# Supplementary material for: Spatially targeted chemokine exocytosis guides transmigration at lymphatic endothelial multicellular junctions
Source: EMBO J. 2024 Jun 14;43(15):4. doi: 10.1038/s44318-024-00129-x (PMC11294460; doi:10.1038/s44318-024-00129-x)
Supplement: Supplementary file 13 — Movie EV11 [file 44318_2024_129_MOESM13_ESM.zip › readme Movie EV11.rtf]

Movie EV11. Phase contrast and epifluorescence microscopy recording of a LEC monolayer expressing CCL21 deltaC-mCherry and transfected with siControl (left panel) or siRAB6 (right panel). The movie shows cell junctions stained with non-blocking VE-cadherin antibody (magenta) and the DC with Hoechst (nuclei, blue). DC transmigration events are marked with white arrowheads. The frame interval is 90’’ and the scale bar is 30µm. The time stamp shows minutes. The movies represent n=10 biological replicates in three independent experiments. The quantification is shown in Fig. 6K.
