# Supplementary material for: Spatially targeted chemokine exocytosis guides transmigration at lymphatic endothelial multicellular junctions
Source: EMBO J. 2024 Jun 14;43(15):4. doi: 10.1038/s44318-024-00129-x (PMC11294460; doi:10.1038/s44318-024-00129-x)
Supplement: Supplementary file 14 — Movie EV12 [file 44318_2024_129_MOESM14_ESM.zip › readme Movie EV12.rtf]

Movie EV12. Phase contrast and epifluorescence microscopy recording of a LEC monolayer expressing full-length CCL21-mCherry and transfected with siControl (left panel) or siRAB6 (right panel). The movie shows cell junctions stained with non-blocking VE-cadherin antibody (magenta) and the DC with Hoechst (nuclei, blue). DC transmigration events are marked with white arrowheads. The time stamp shows minutes and seconds. The frame interval is 90’’ and the scale bar is 30µm. The movies represent n=11 (siControl) and n=9 (siRAB6) biological replicates in three independent experiments. The quantification is shown in Fig. 6K.
