# Supplementary material for: Spatially targeted chemokine exocytosis guides transmigration at lymphatic endothelial multicellular junctions
Source: EMBO J. 2024 Jun 14;43(15):4. doi: 10.1038/s44318-024-00129-x (PMC11294460; doi:10.1038/s44318-024-00129-x)
Supplement: Supplementary file 15 — Movie EV13 [file 44318_2024_129_MOESM15_ESM.zip › readme Movie EV13.rtf]

Movie EV13. Epifluorescence microscopy recording of a LEC monolayer expressing CCL21-mCherry and EGFP (left panel) or EGFP-RAB8A-DN (right panel). The movie shows the EGFP channel (green), the cell junctions stained with non-blocking VE-cadherin antibody (magenta) and the DC with Hoechst (nuclei, blue). DC transmigration events are marked with white arrowheads. The frame interval is 90’’ and the scale bar is 30µm. Time stamp shows minutes. For the EGFP-Control movie n=10 biological replicates and for EGFP-RAB8A DN n=9 biological replicates, both, in 4 experiments. The movie is related to Fig. 7L-M.
