# Supplementary figures and images for: Spatially targeted chemokine exocytosis guides transmigration at lymphatic endothelial multicellular junctions
Source: EMBO J. 2024 Jun 14;43(15):4. doi: 10.1038/s44318-024-00129-x (PMC11294460; doi:10.1038/s44318-024-00129-x)

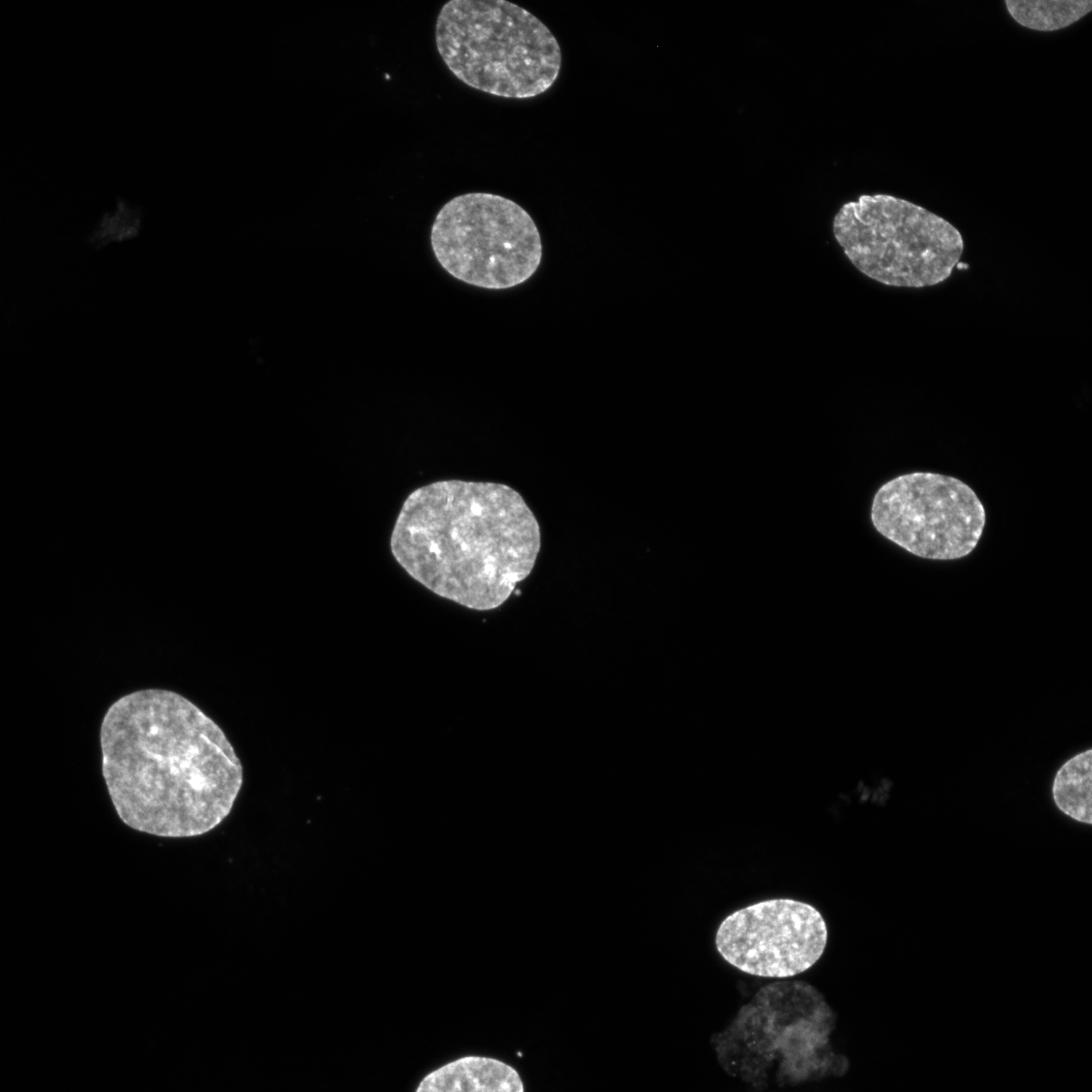

Supplement: Supplementary file 17 — Source data Fig. 2 [file 44318_2024_129_MOESM17_ESM.zip › Figure 2/2G/Figure 2G.tif]

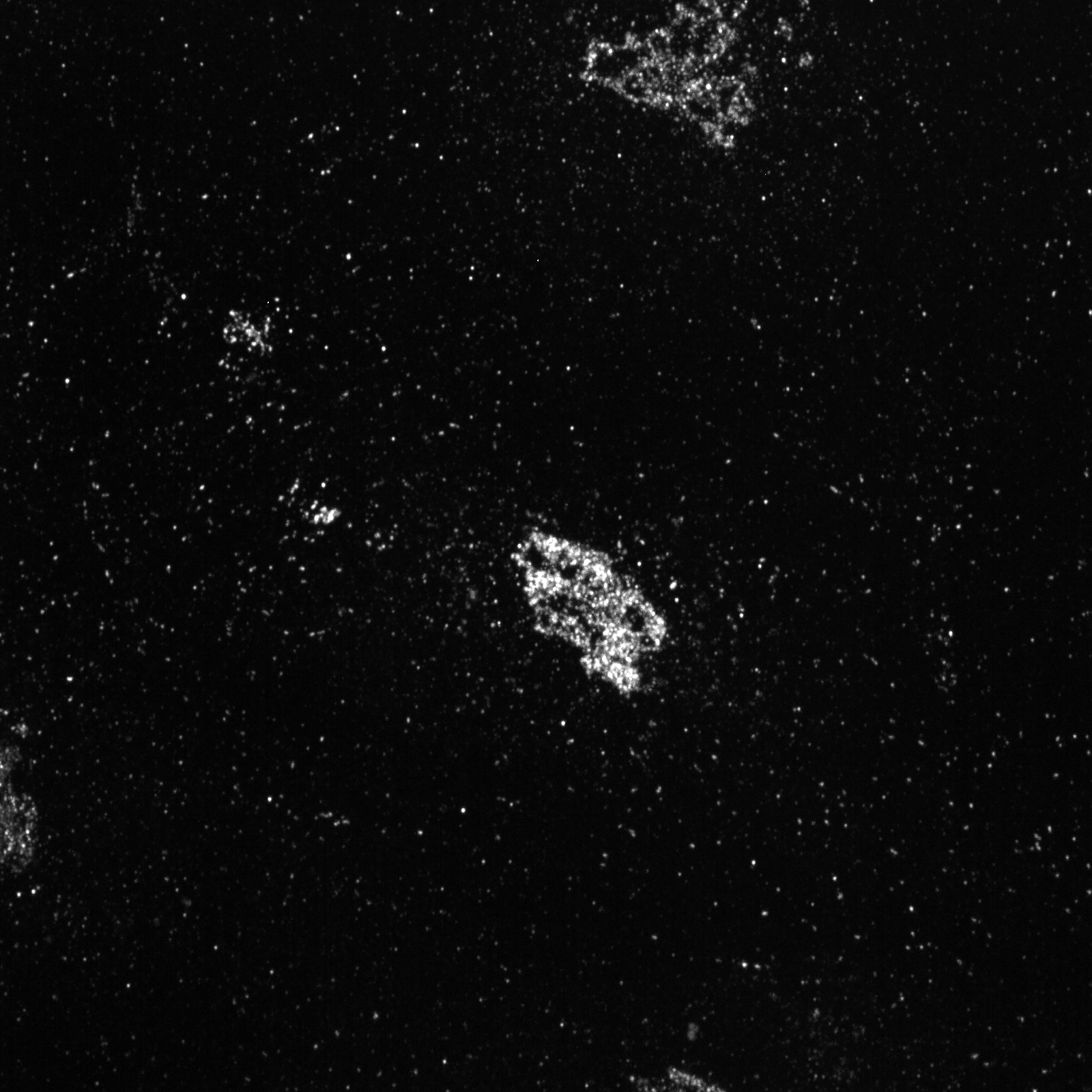

Supplement: Supplementary file 17 — Source data Fig. 2 [file 44318_2024_129_MOESM17_ESM.zip › Figure 2/2I/Figure 2I.tif]

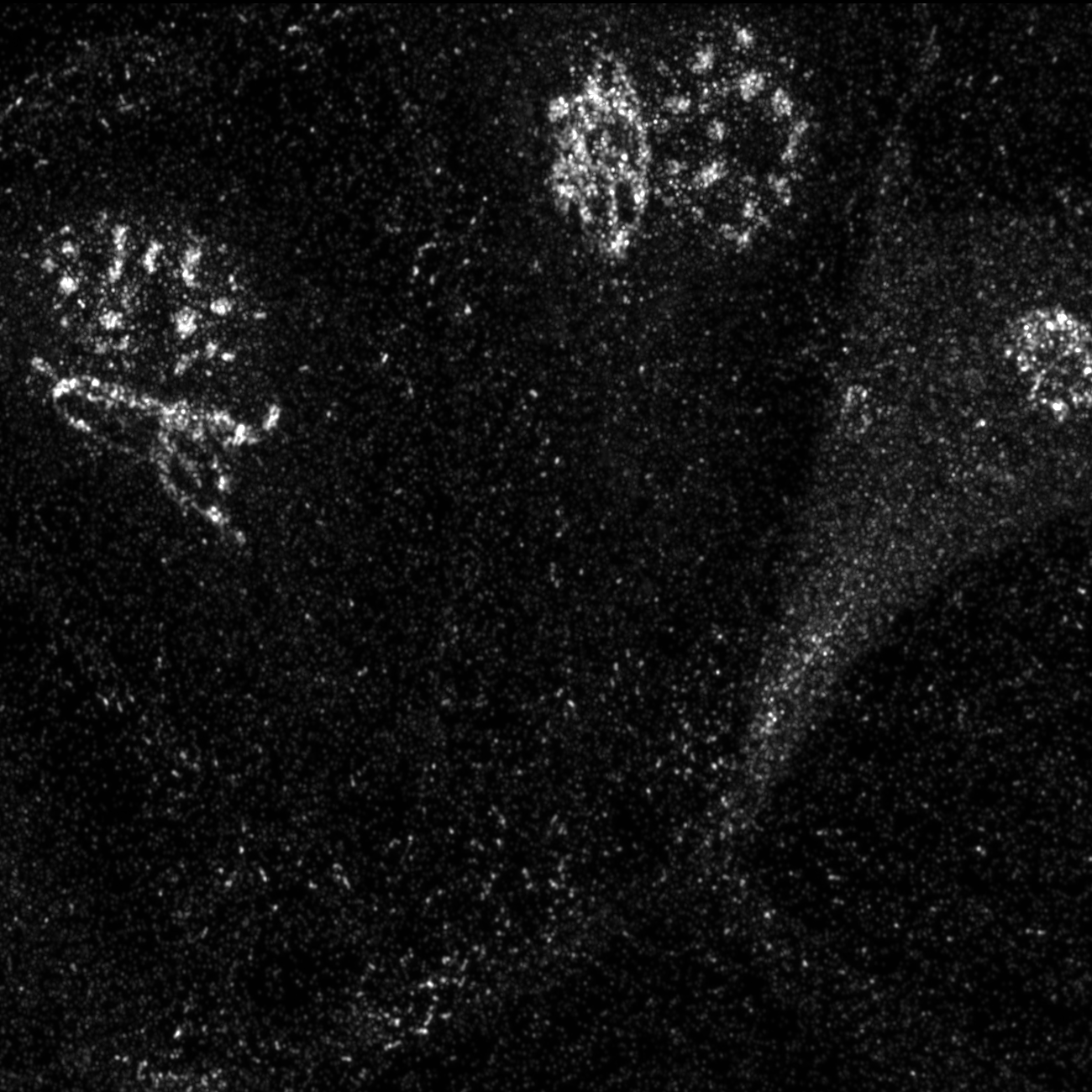

Supplement: Supplementary file 17 — Source data Fig. 2 [file 44318_2024_129_MOESM17_ESM.zip › Figure 2/2H/Figure 2H.tif]

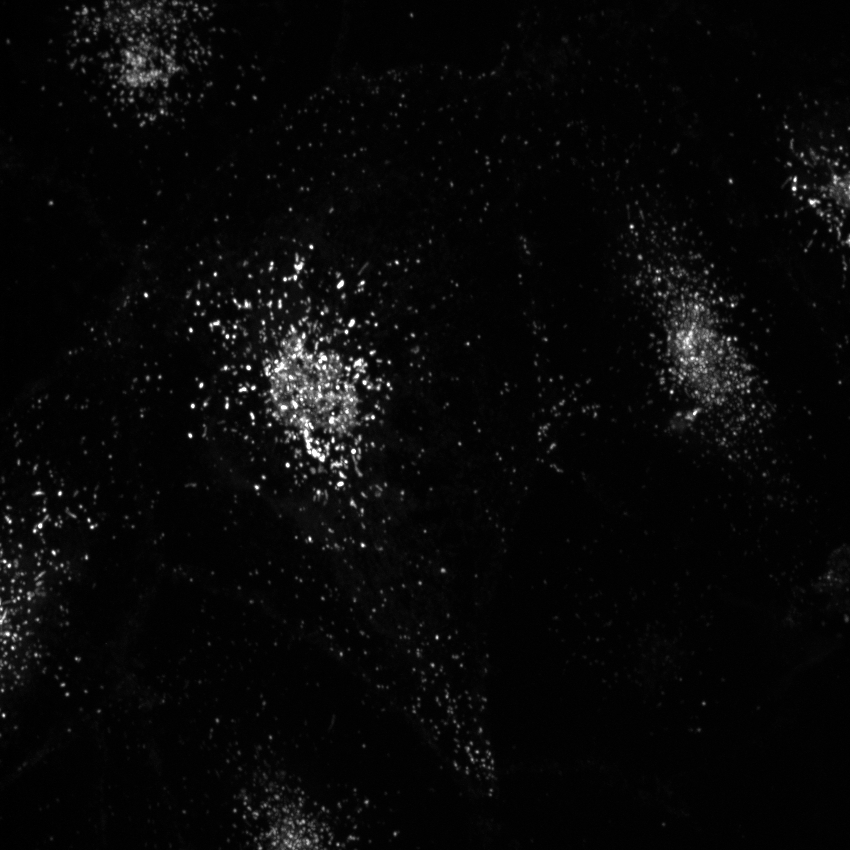

Supplement: Supplementary file 17 — Source data Fig. 2 [file 44318_2024_129_MOESM17_ESM.zip › Figure 2/2A/Figure 2A.tif]

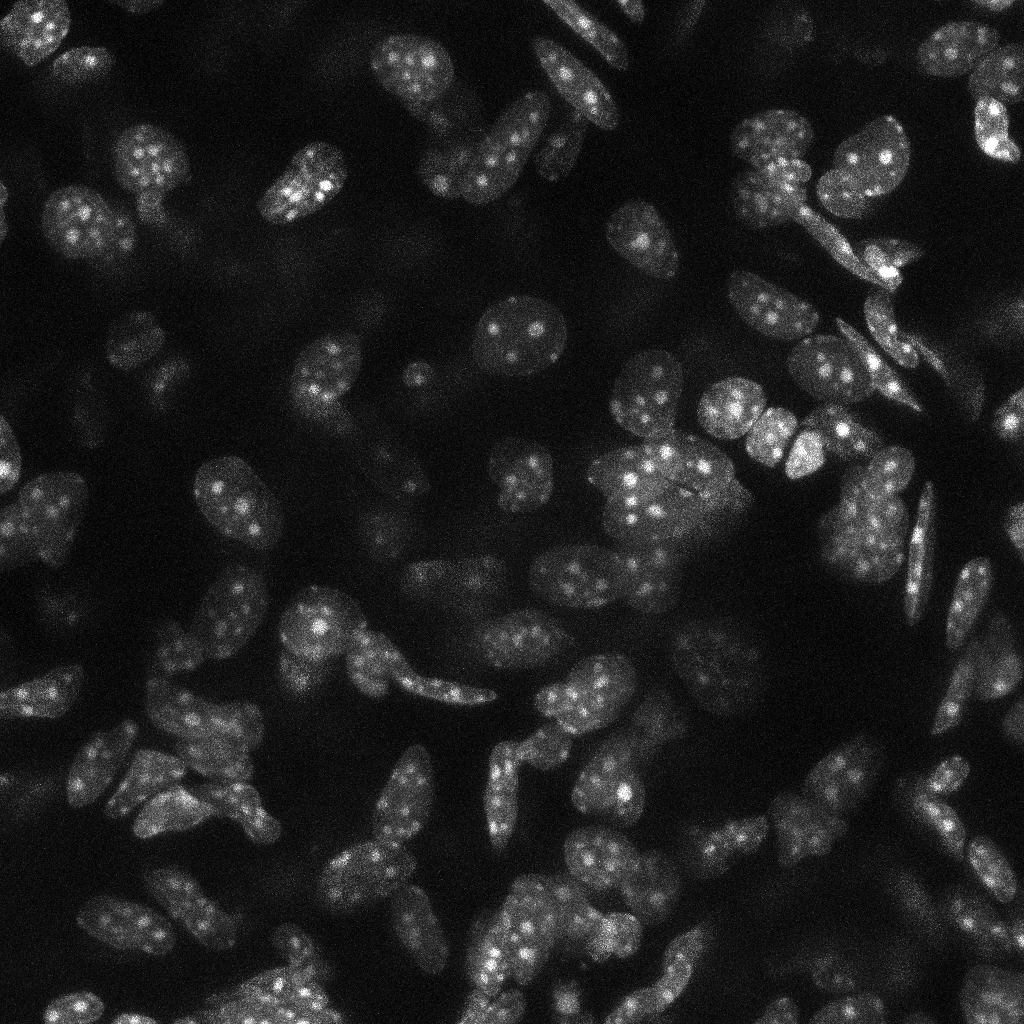

Supplement: Supplementary file 17 — Source data Fig. 2 [file 44318_2024_129_MOESM17_ESM.zip › Figure 2/2J/Figure 2J-overview.tif]

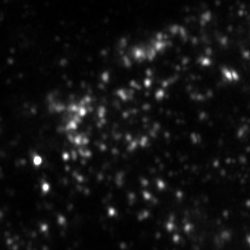

Supplement: Supplementary file 17 — Source data Fig. 2 [file 44318_2024_129_MOESM17_ESM.zip › Figure 2/2J/Figure 2J-zoom.tif]

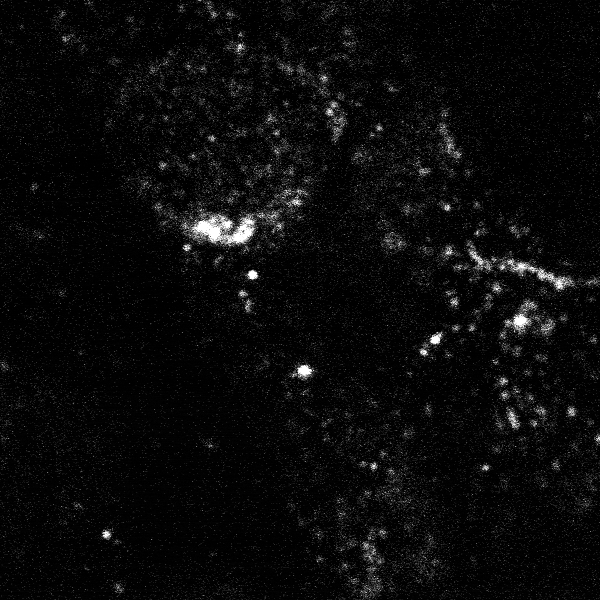

Supplement: Supplementary file 17 — Source data Fig. 2 [file 44318_2024_129_MOESM17_ESM.zip › Figure 2/2K/Figure 2K-kept stack-7-17.tif]

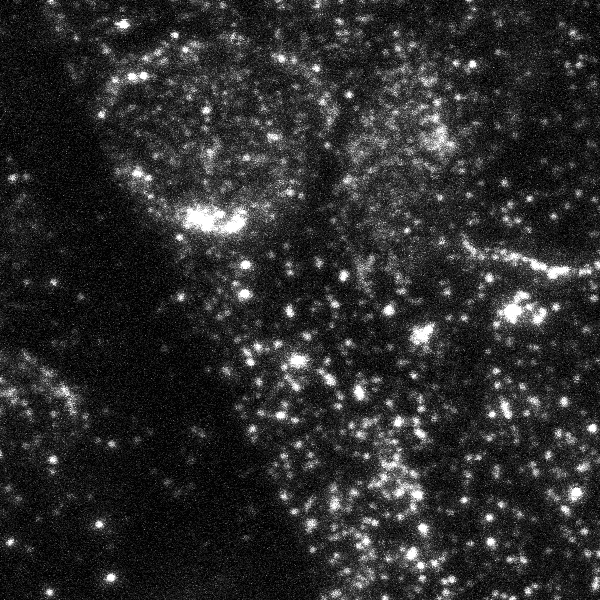

Supplement: Supplementary file 17 — Source data Fig. 2 [file 44318_2024_129_MOESM17_ESM.zip › Figure 2/2K/Figure 2K.tif]

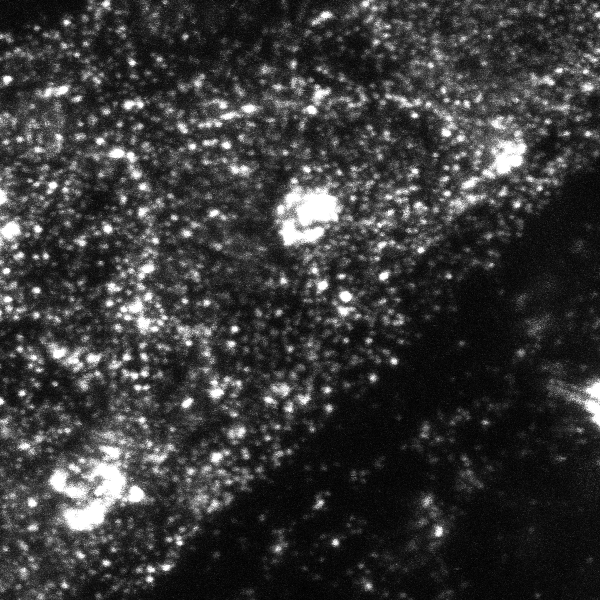

Supplement: Supplementary file 17 — Source data Fig. 2 [file 44318_2024_129_MOESM17_ESM.zip › Figure 2/2L/Figure 2L.tif]

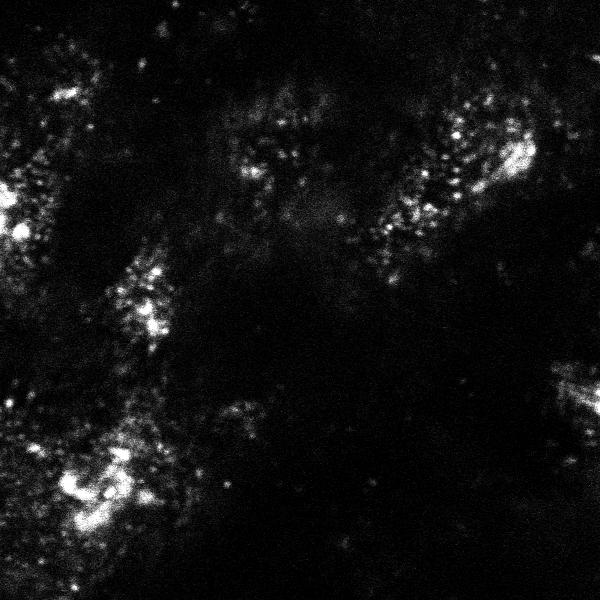

Supplement: Supplementary file 17 — Source data Fig. 2 [file 44318_2024_129_MOESM17_ESM.zip › Figure 2/2L/Figure 2L-kept stack12-24.tif]

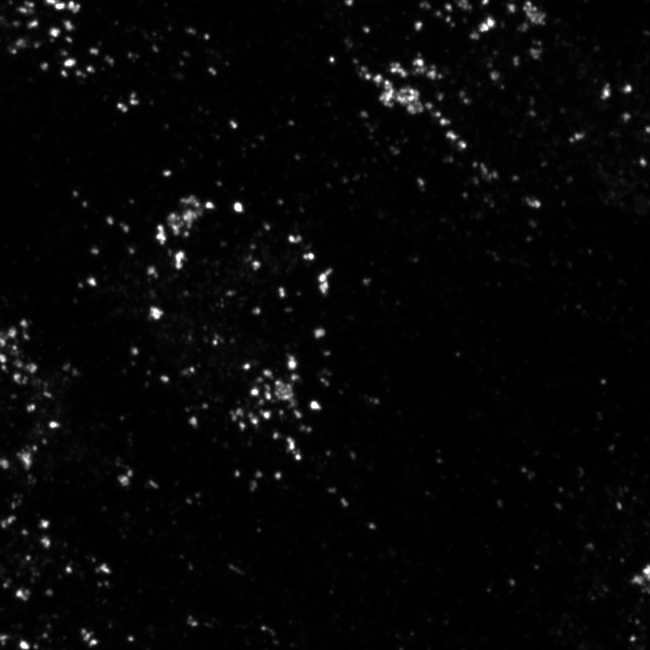

Supplement: Supplementary file 18 — Source data Fig. 3 [file 44318_2024_129_MOESM18_ESM.zip › Figure 3/3E/Figure 3E.tif]

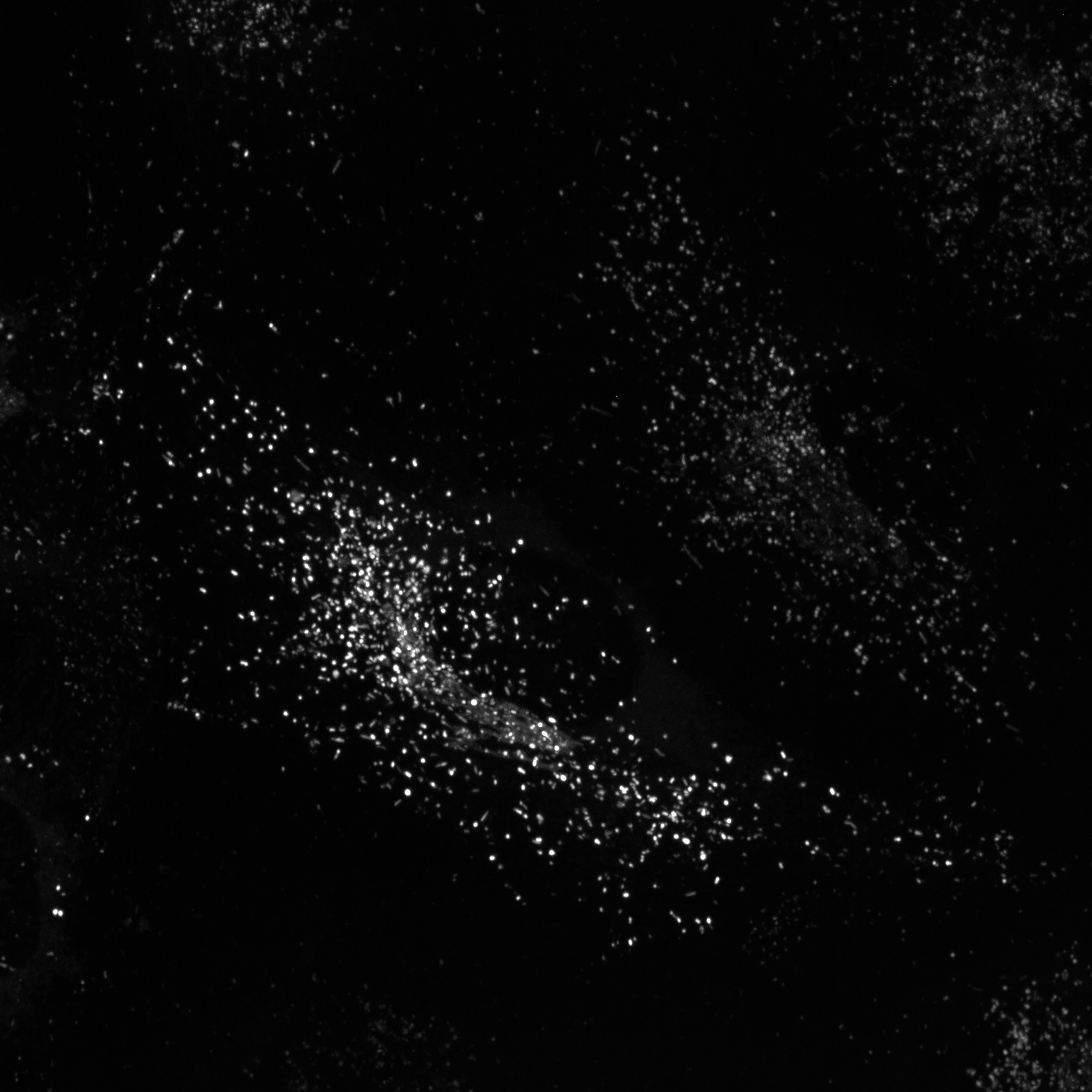

Supplement: Supplementary file 18 — Source data Fig. 3 [file 44318_2024_129_MOESM18_ESM.zip › Figure 3/3D/Figure 3D.tif]

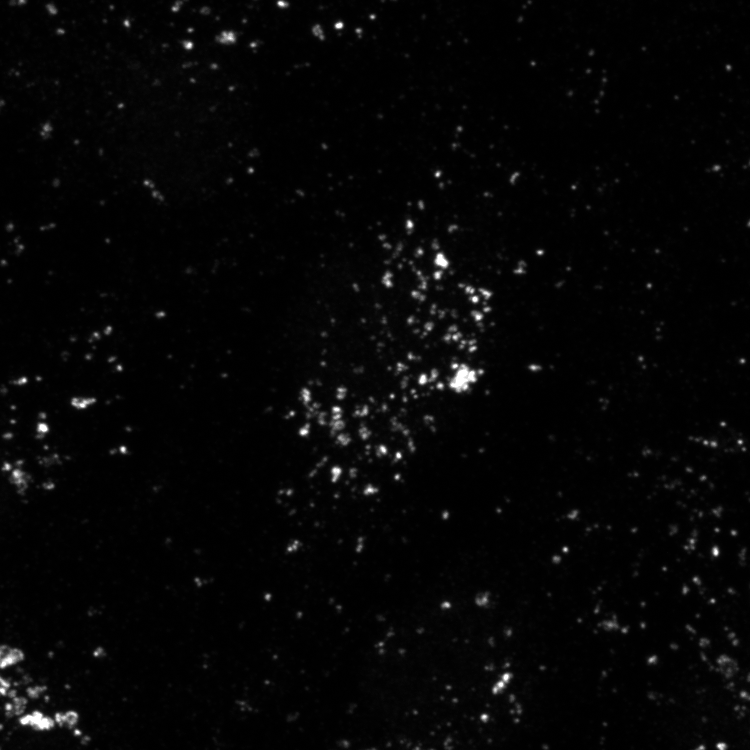

Supplement: Supplementary file 18 — Source data Fig. 3 [file 44318_2024_129_MOESM18_ESM.zip › Figure 3/3F/Figure 3F.tif]

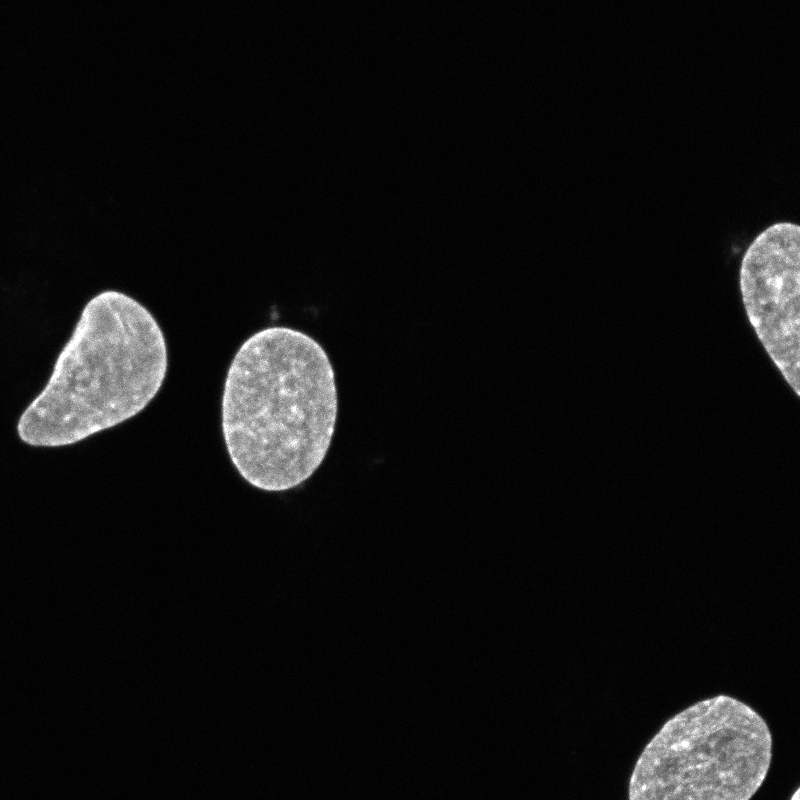

Supplement: Supplementary file 18 — Source data Fig. 3 [file 44318_2024_129_MOESM18_ESM.zip › Figure 3/3I/Figure 3I.tif]

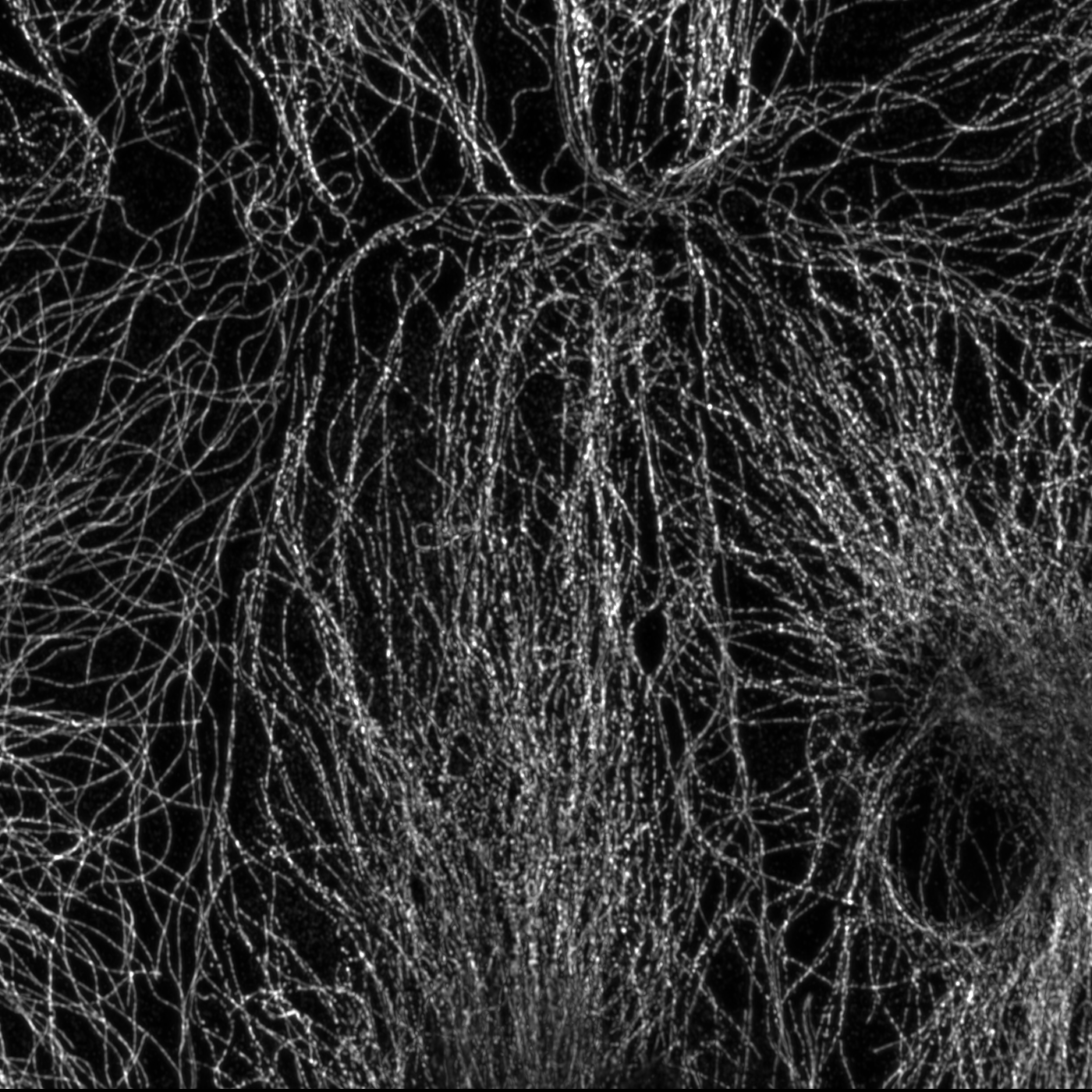

Supplement: Supplementary file 19 — Source data Fig. 4 [file 44318_2024_129_MOESM19_ESM.zip › Figure 4/4B/Figure 4B.tif]

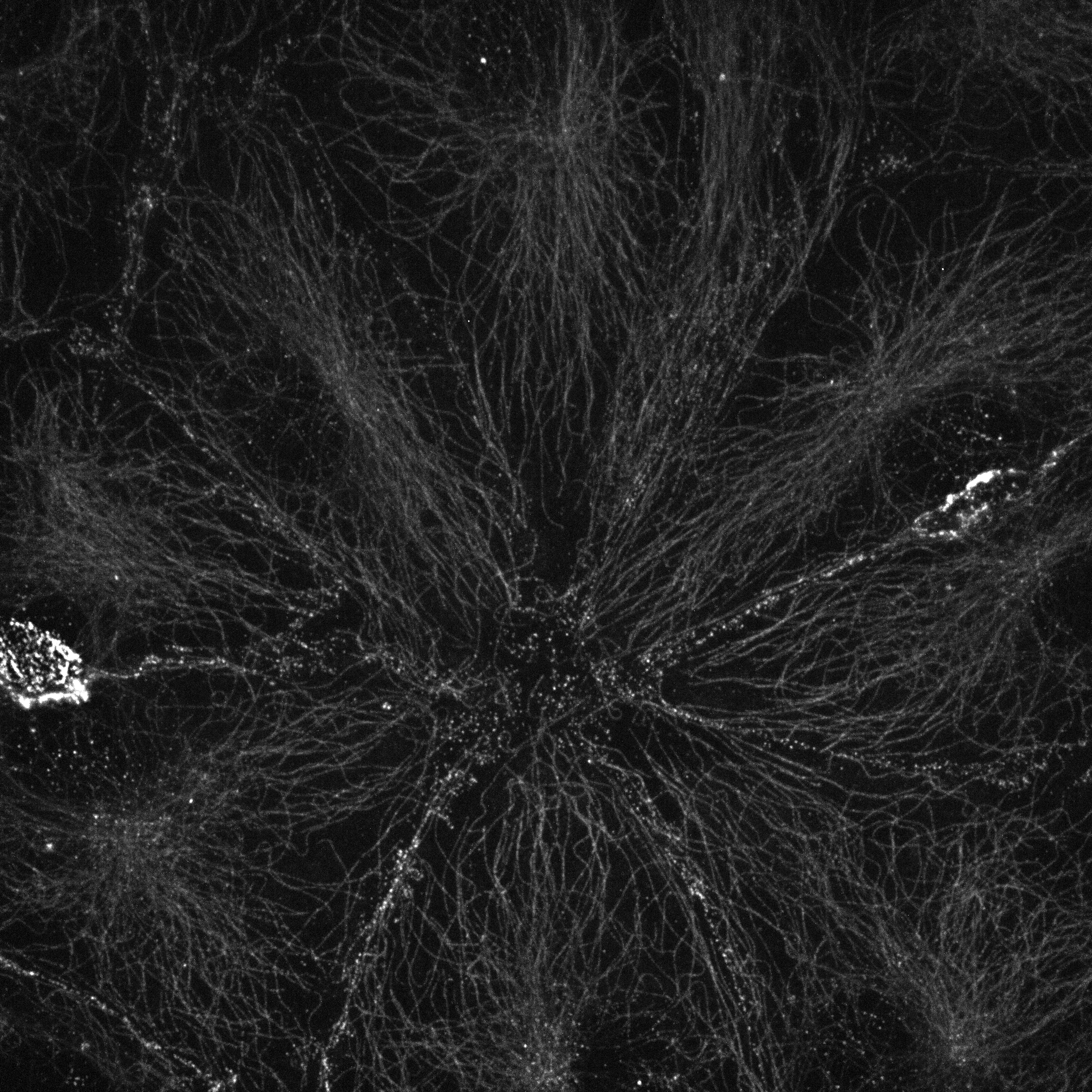

Supplement: Supplementary file 19 — Source data Fig. 4 [file 44318_2024_129_MOESM19_ESM.zip › Figure 4/4C/Figure 4C.tif]

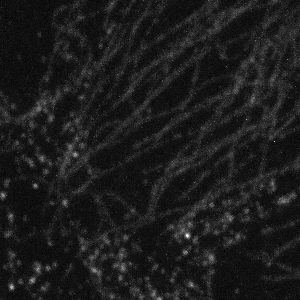

Supplement: Supplementary file 19 — Source data Fig. 4 [file 44318_2024_129_MOESM19_ESM.zip › Figure 4/4C/Figure 4C-insert.tif]

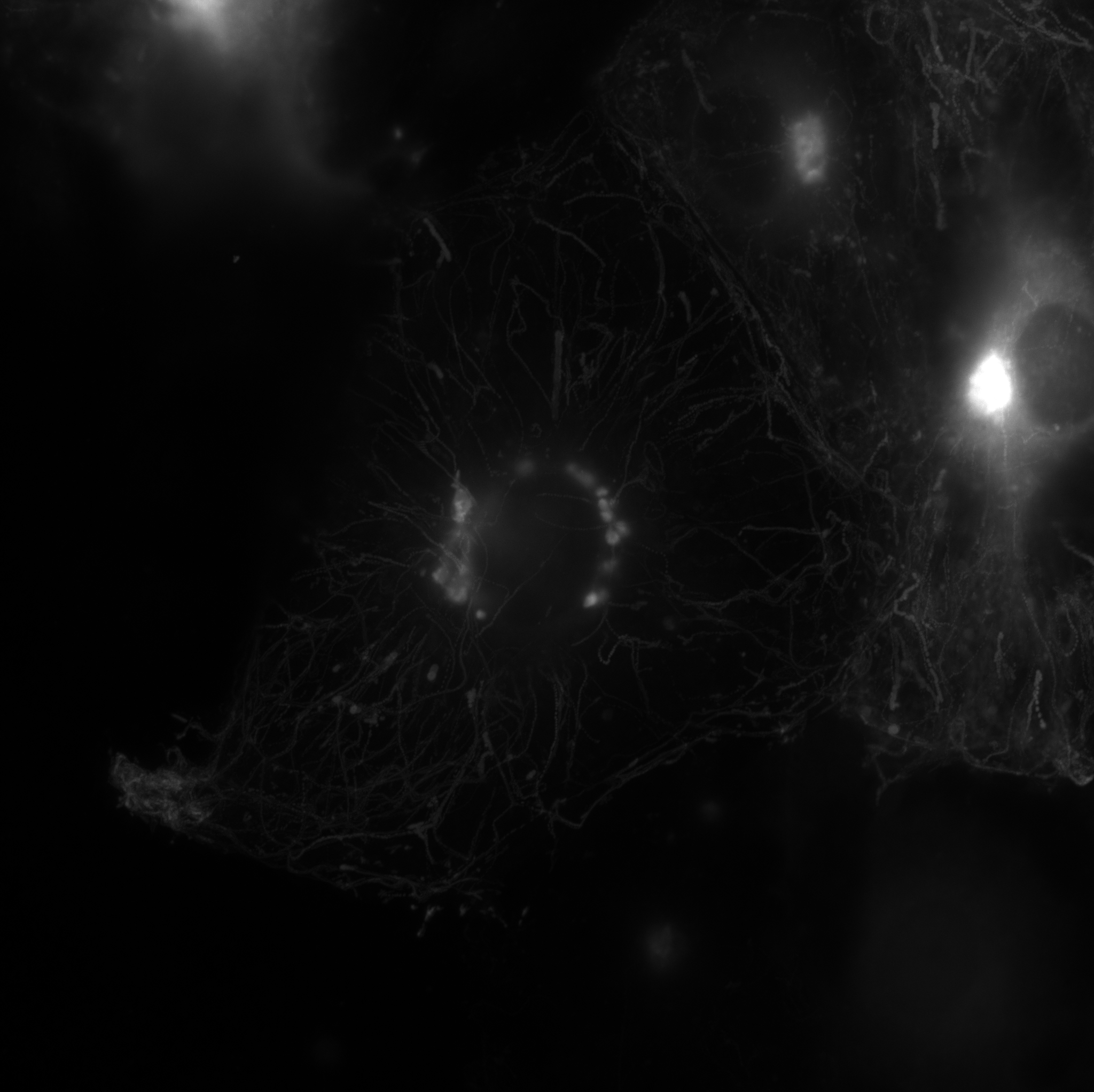

Supplement: Supplementary file 19 — Source data Fig. 4 [file 44318_2024_129_MOESM19_ESM.zip › Figure 4/4A/Figure 4A.tif]

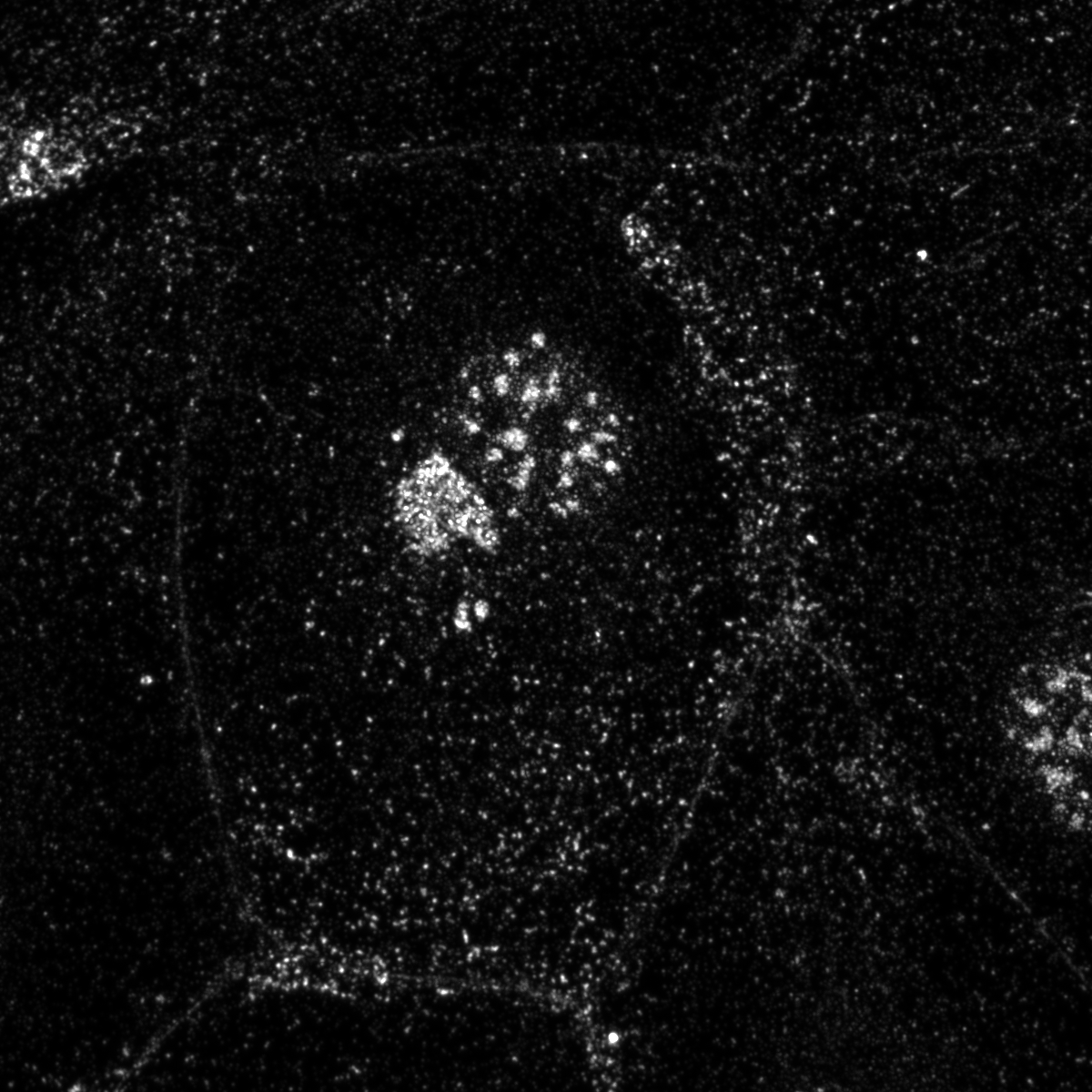

Supplement: Supplementary file 20 — Source data Fig. 5 [file 44318_2024_129_MOESM20_ESM.zip › Figure 5/5I/Figure 5I-siELKS08.tif]

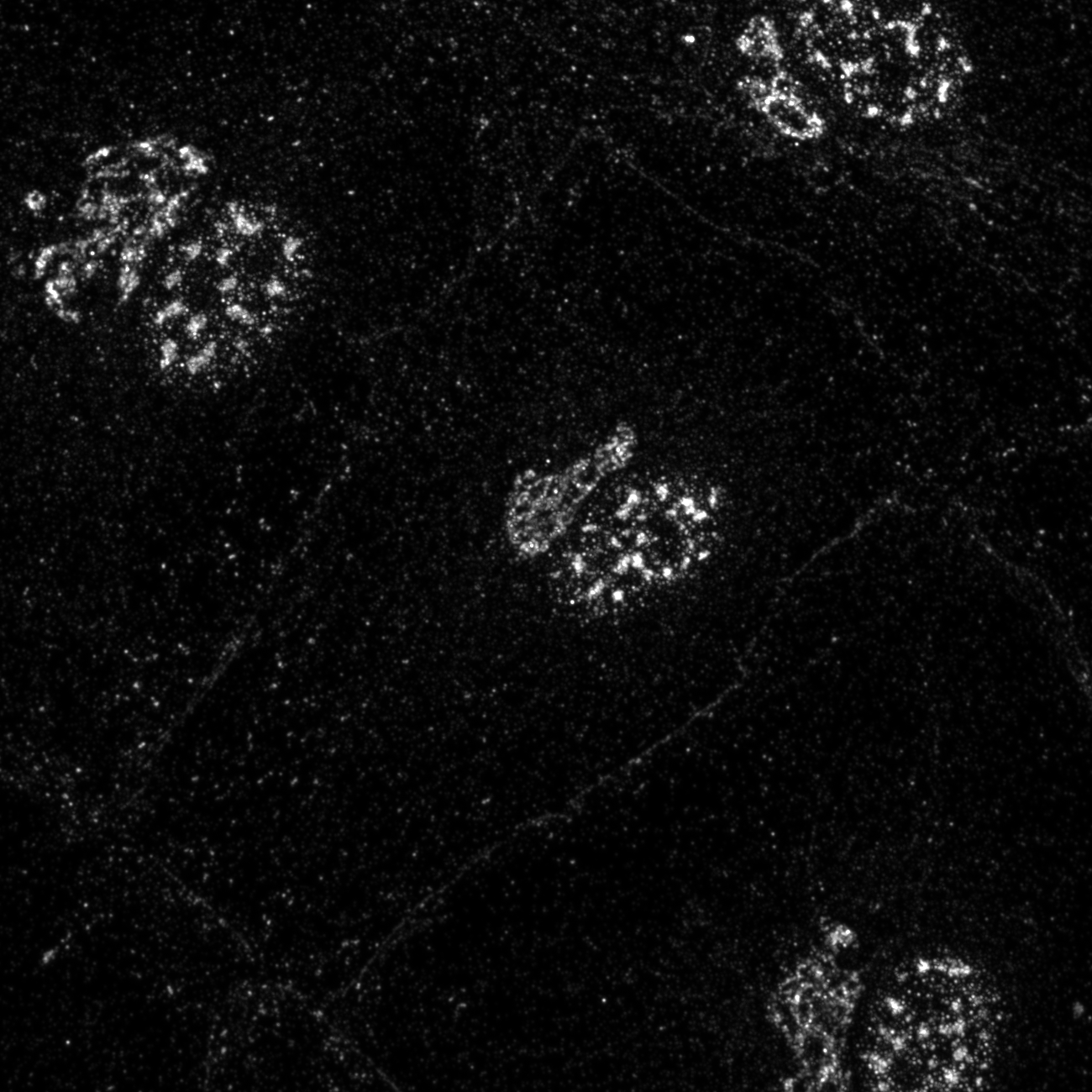

Supplement: Supplementary file 20 — Source data Fig. 5 [file 44318_2024_129_MOESM20_ESM.zip › Figure 5/5I/Figure 5I-siCTRL01.tif]

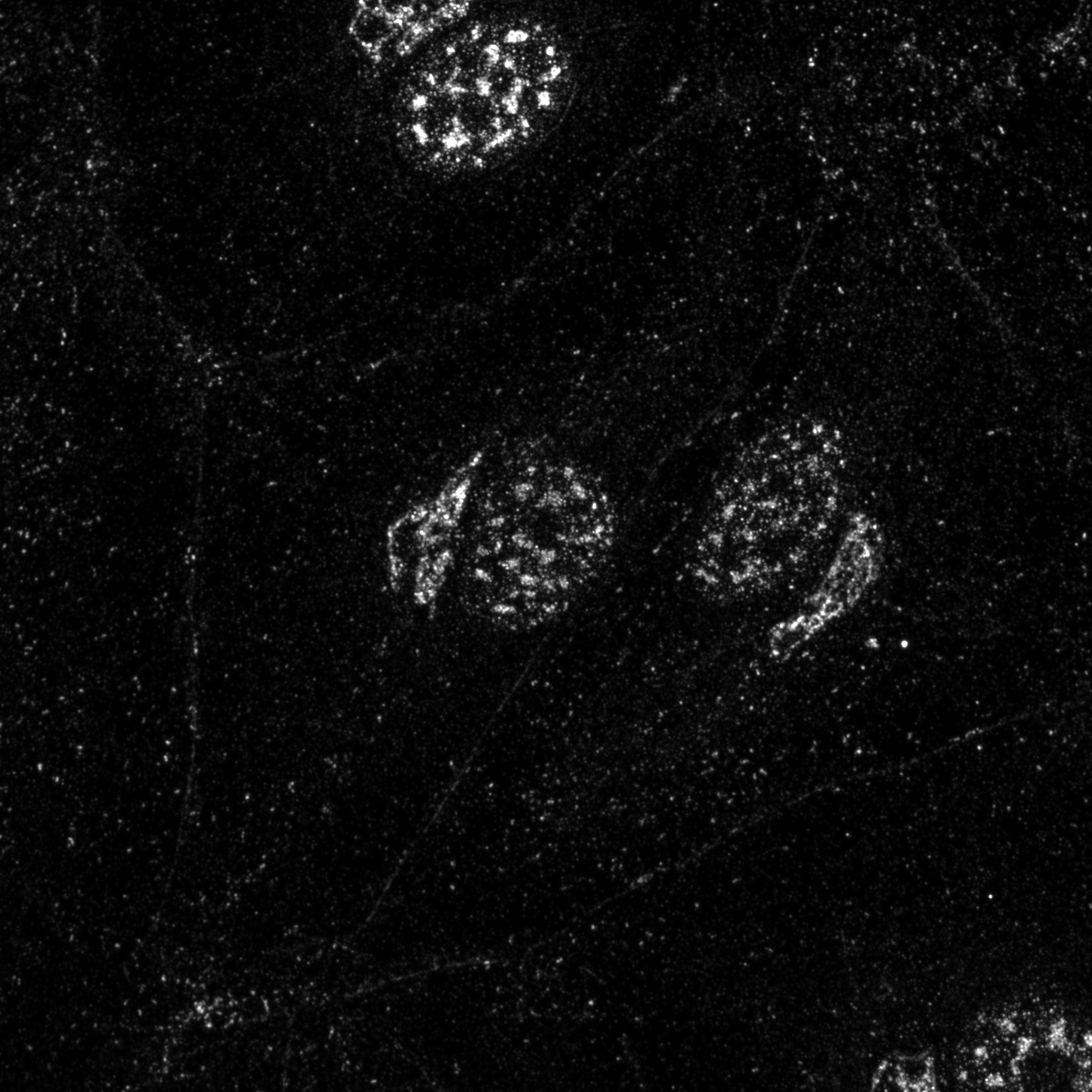

Supplement: Supplementary file 20 — Source data Fig. 5 [file 44318_2024_129_MOESM20_ESM.zip › Figure 5/5I/Figure 5I-siCTRL02.tif]

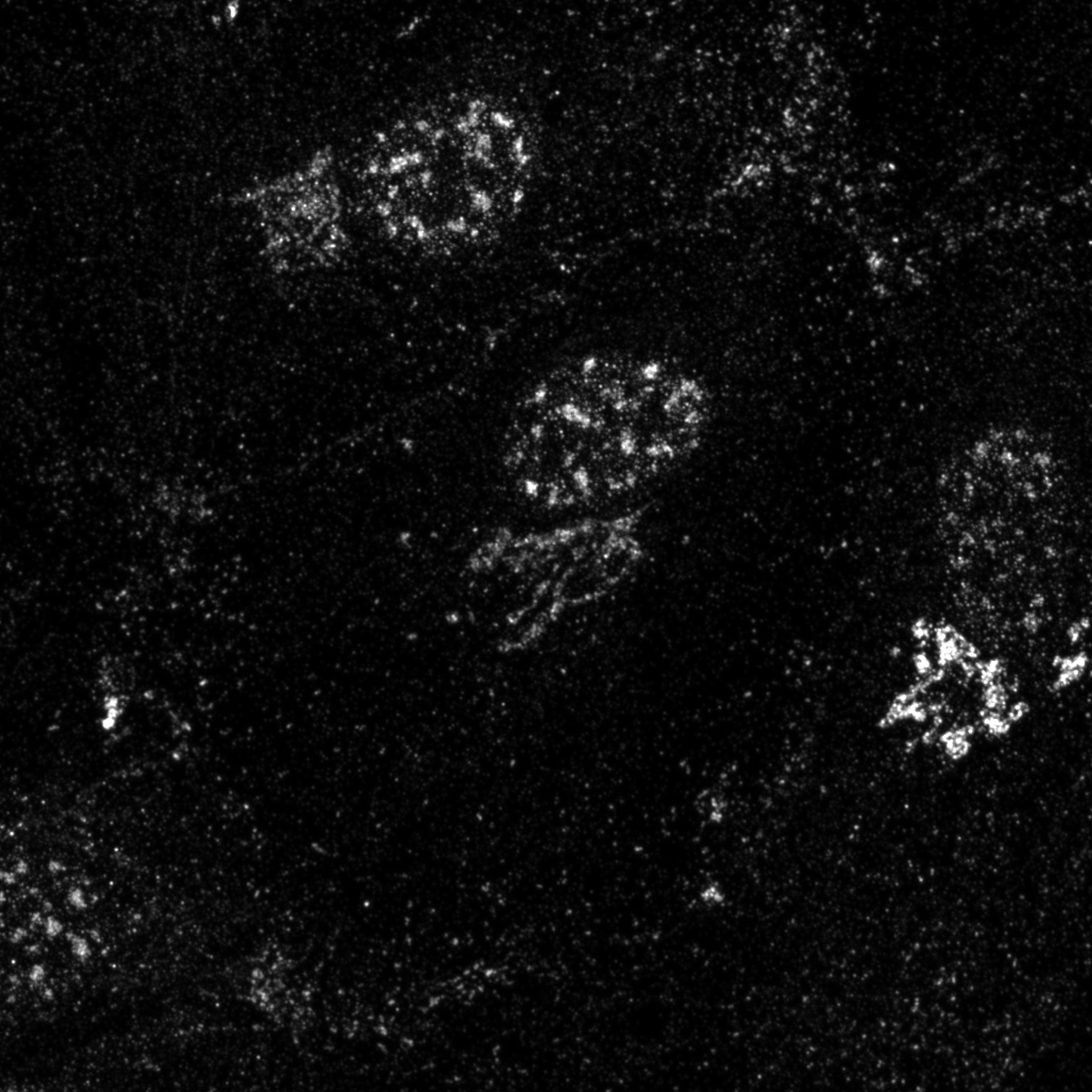

Supplement: Supplementary file 20 — Source data Fig. 5 [file 44318_2024_129_MOESM20_ESM.zip › Figure 5/5I/Figure 5I-siELKS06.tif]

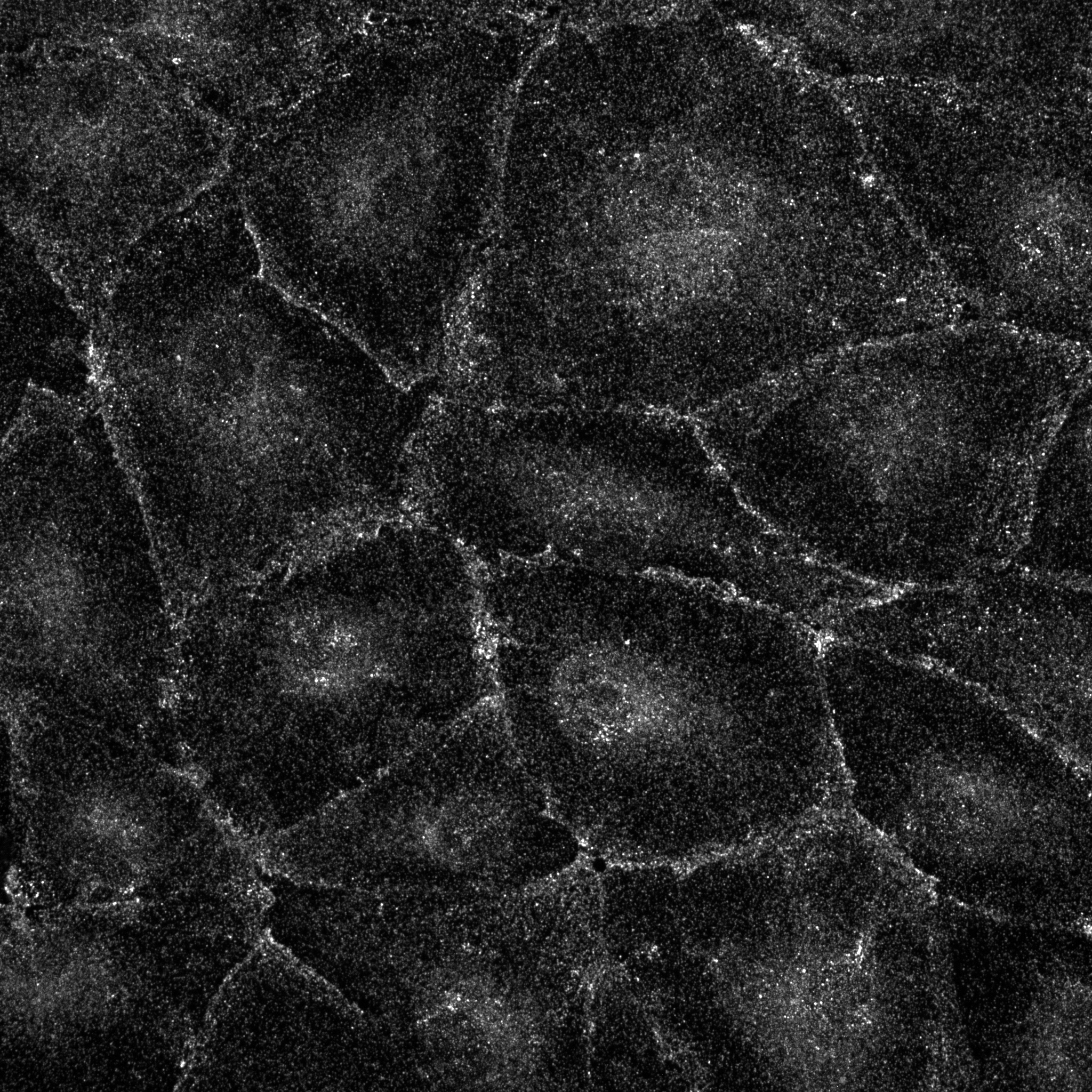

Supplement: Supplementary file 20 — Source data Fig. 5 [file 44318_2024_129_MOESM20_ESM.zip › Figure 5/5A/Figure 5A.tif]

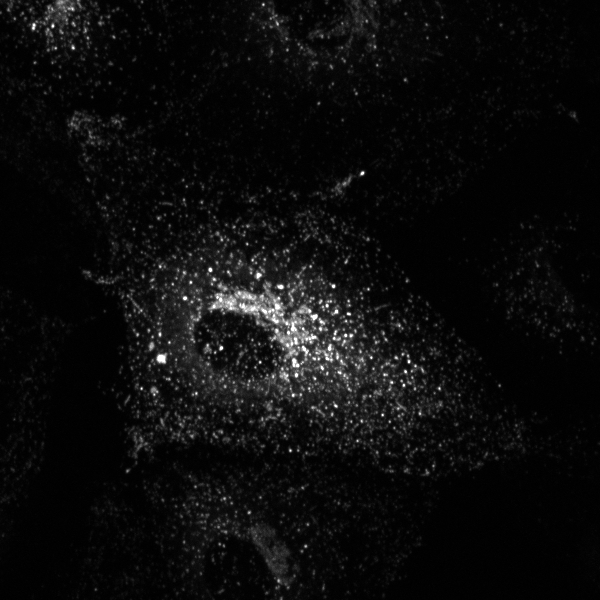

Supplement: Supplementary file 20 — Source data Fig. 5 [file 44318_2024_129_MOESM20_ESM.zip › Figure 5/5E/Figure 5E-siELKS08.tif]

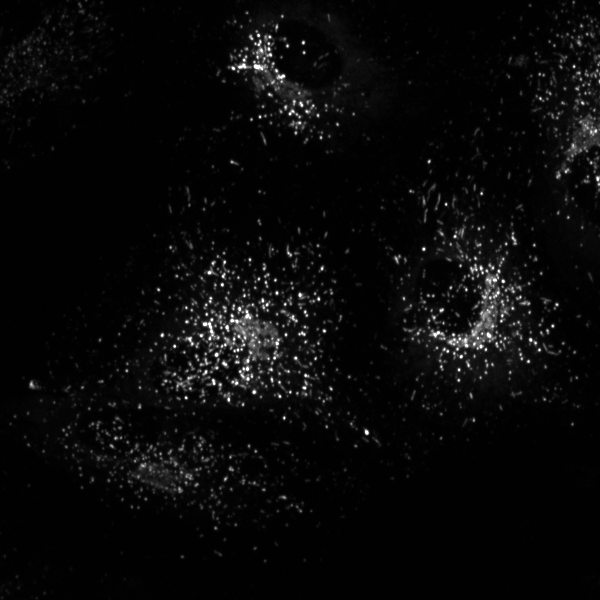

Supplement: Supplementary file 20 — Source data Fig. 5 [file 44318_2024_129_MOESM20_ESM.zip › Figure 5/5E/Figure 5E-siCTRL02.tif]

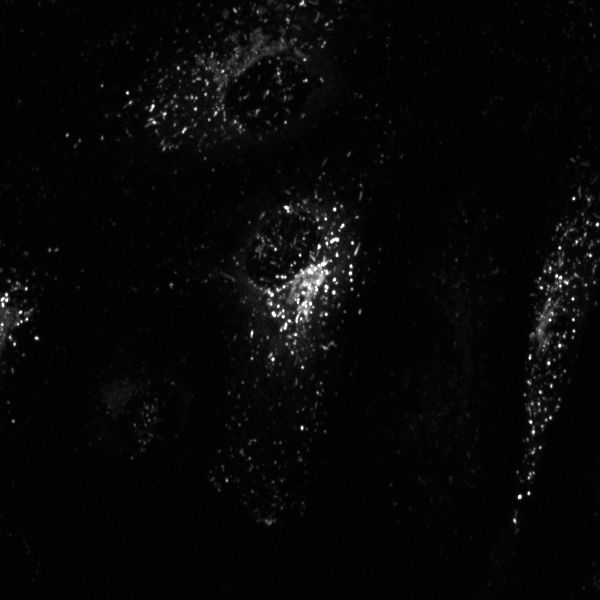

Supplement: Supplementary file 20 — Source data Fig. 5 [file 44318_2024_129_MOESM20_ESM.zip › Figure 5/5E/Figure 5E-siCTRL01.tif]

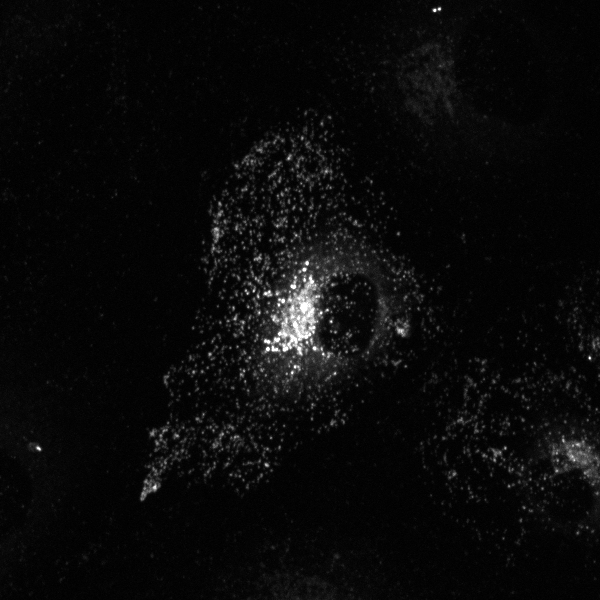

Supplement: Supplementary file 20 — Source data Fig. 5 [file 44318_2024_129_MOESM20_ESM.zip › Figure 5/5E/Figure 5E-siELKS06.tif]

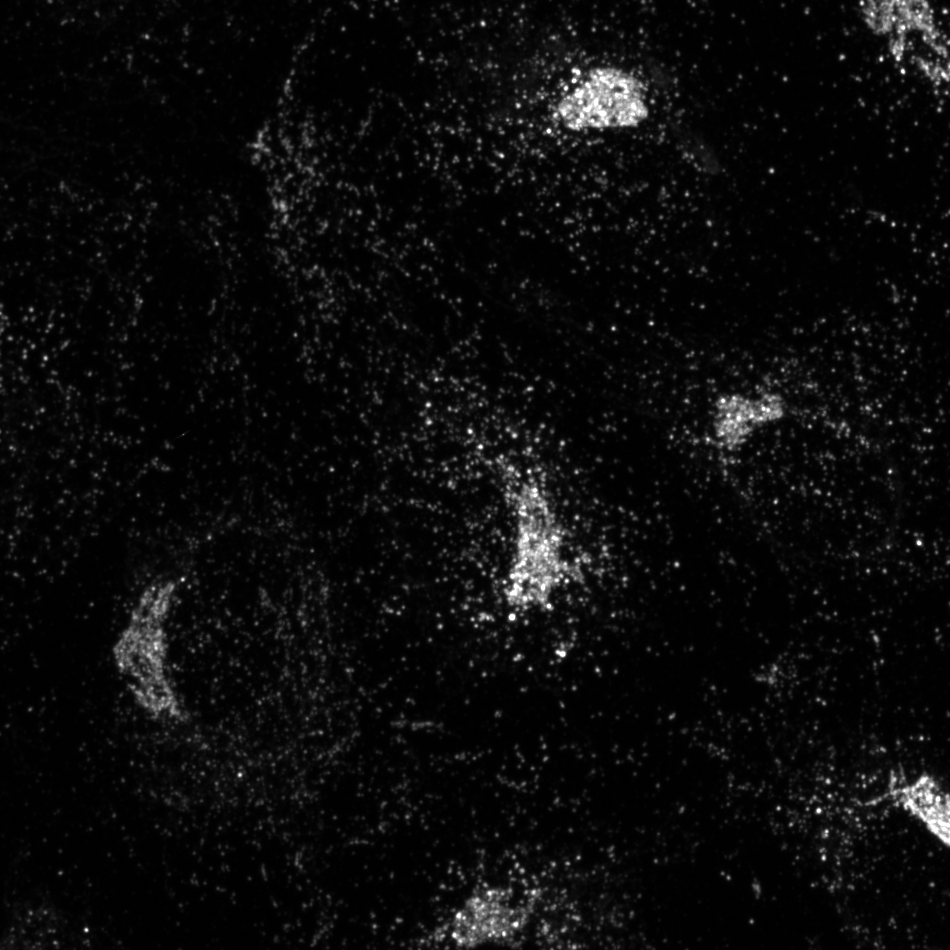

Supplement: Supplementary file 21 — Source data Fig. 6 [file 44318_2024_129_MOESM21_ESM.zip › Figure 6/6G/Figure 6G-siELKS1.tif]

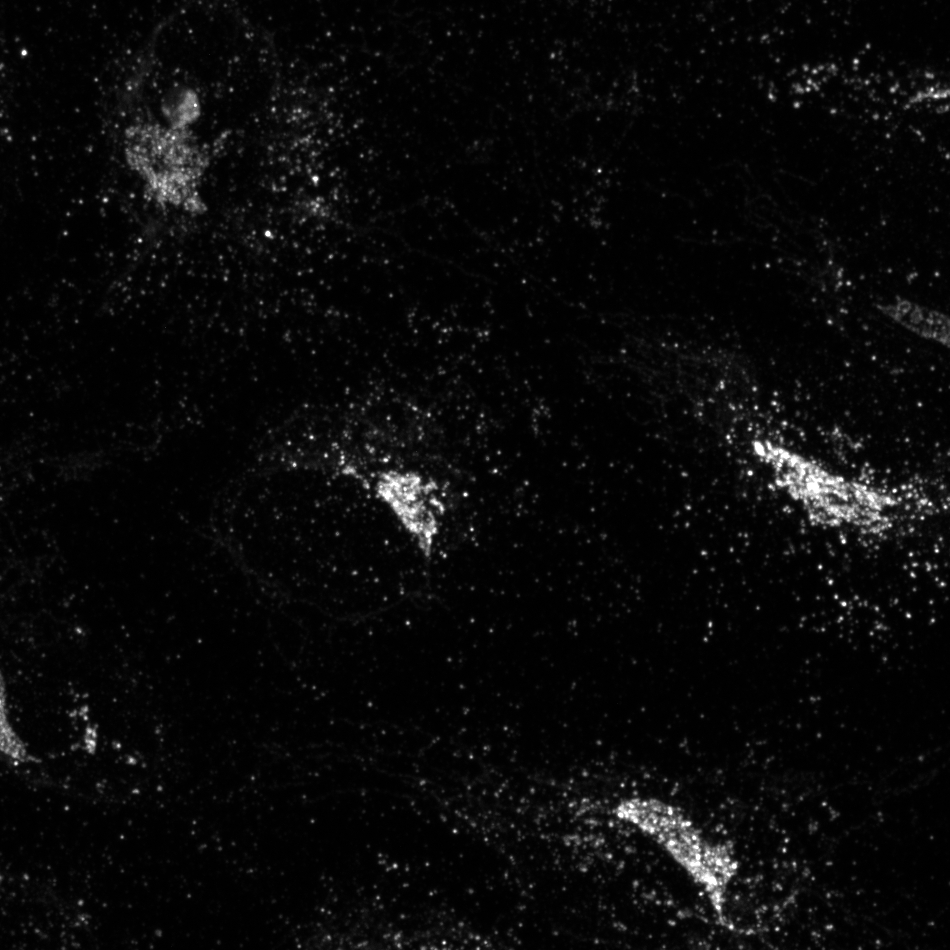

Supplement: Supplementary file 21 — Source data Fig. 6 [file 44318_2024_129_MOESM21_ESM.zip › Figure 6/6G/Figure 6G-siCTRL.tif]

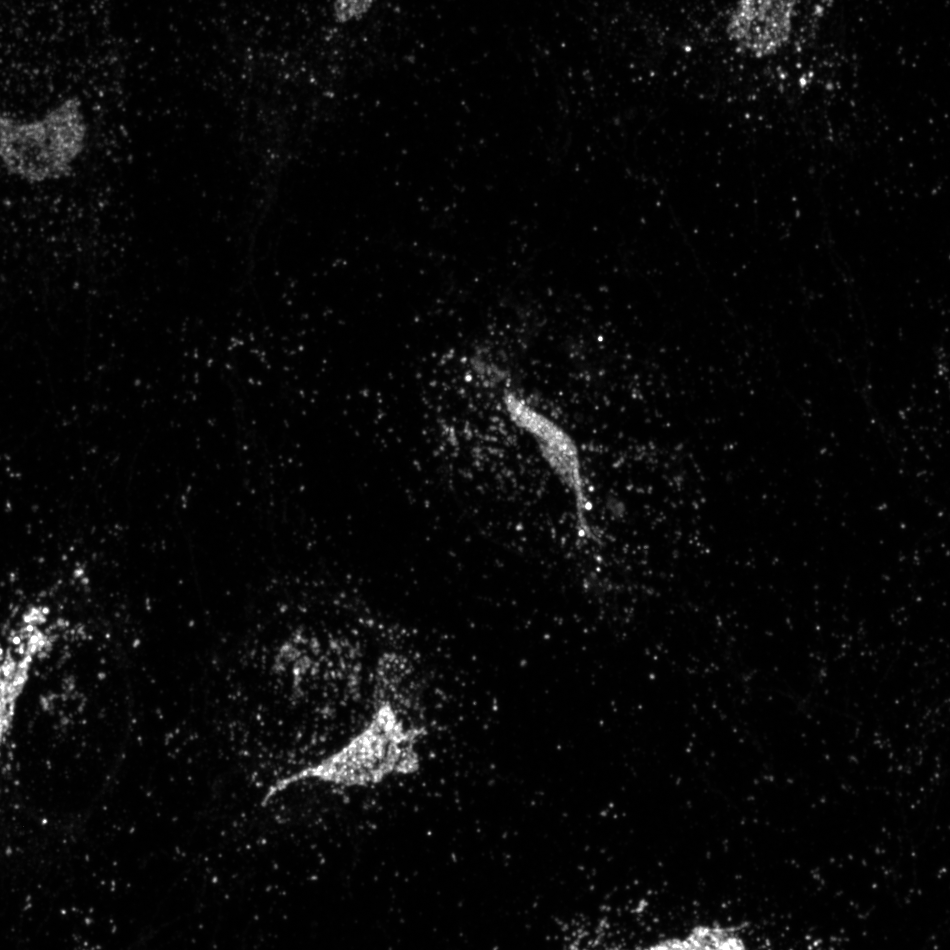

Supplement: Supplementary file 21 — Source data Fig. 6 [file 44318_2024_129_MOESM21_ESM.zip › Figure 6/6G/Figure 6G-siRab6A.tif]

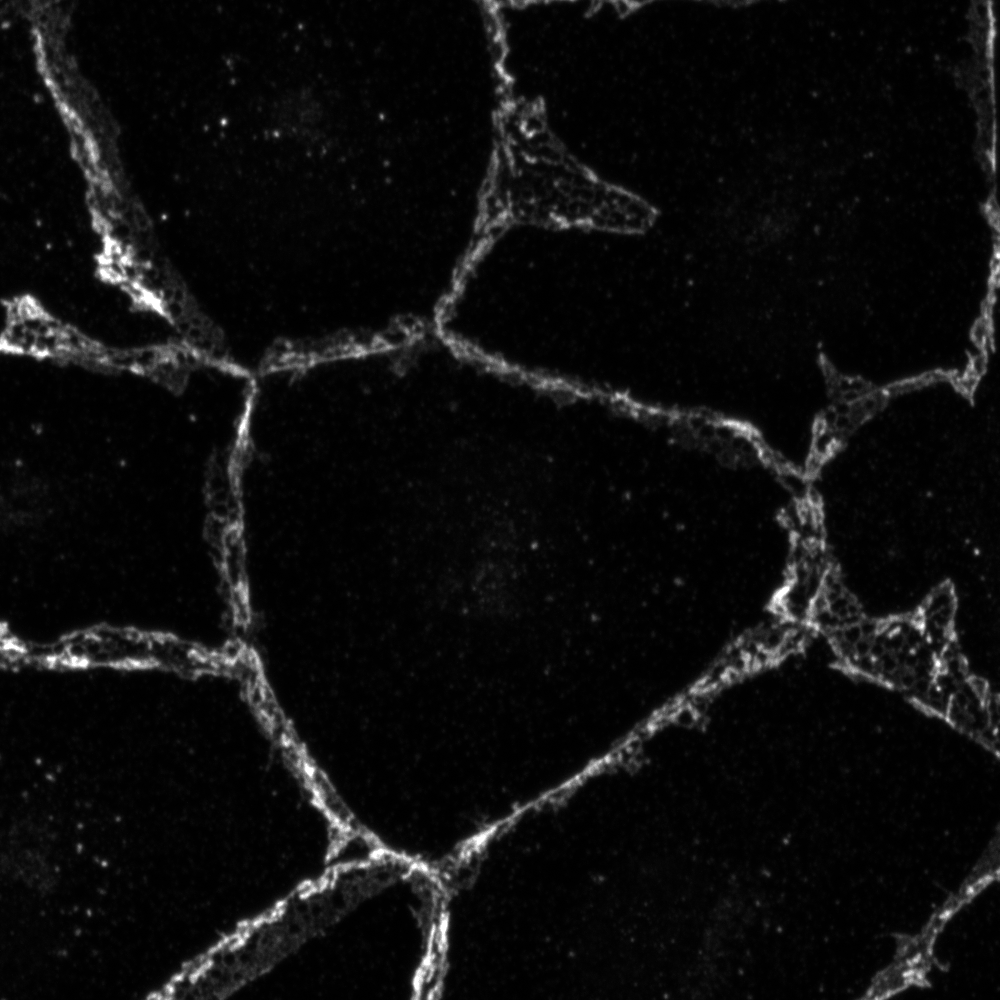

Supplement: Supplementary file 21 — Source data Fig. 6 [file 44318_2024_129_MOESM21_ESM.zip › Figure 6/6C/Figure 6C-siCTRL.tif]

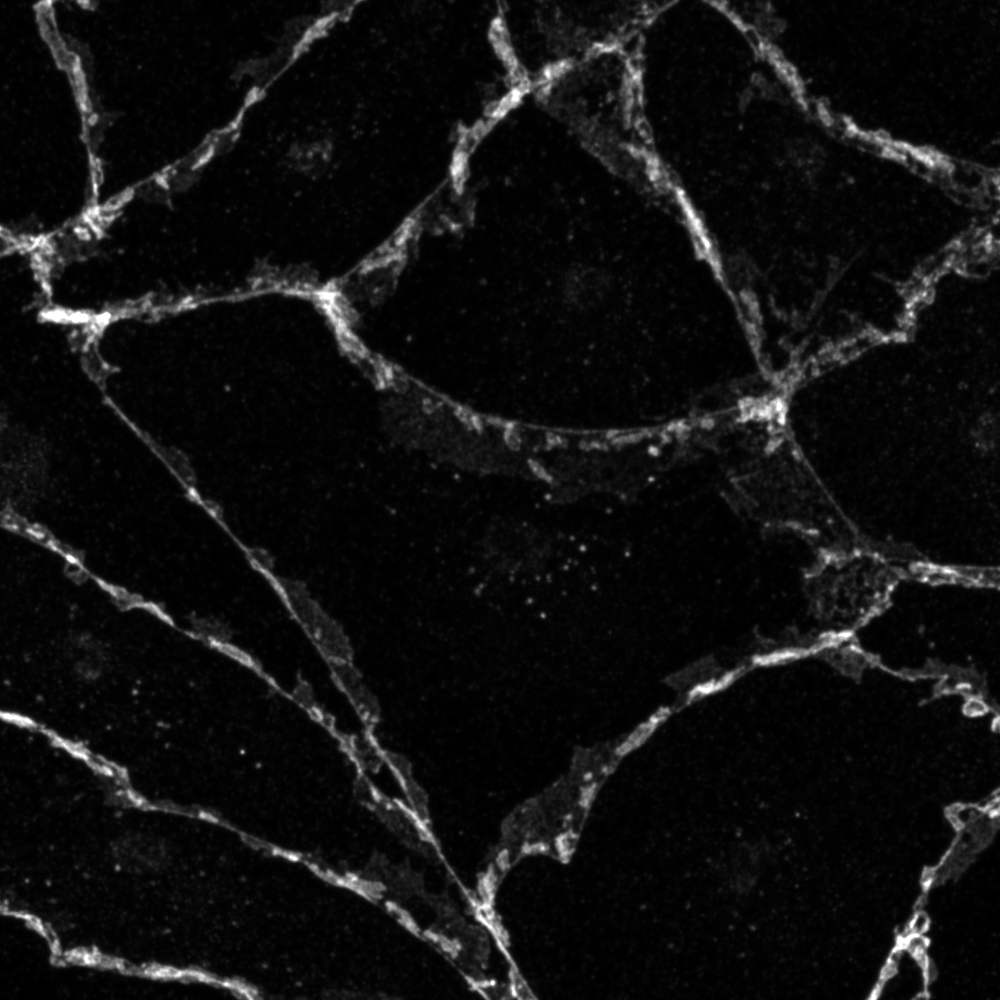

Supplement: Supplementary file 21 — Source data Fig. 6 [file 44318_2024_129_MOESM21_ESM.zip › Figure 6/6C/Figure 6C-siRAB6.tif]

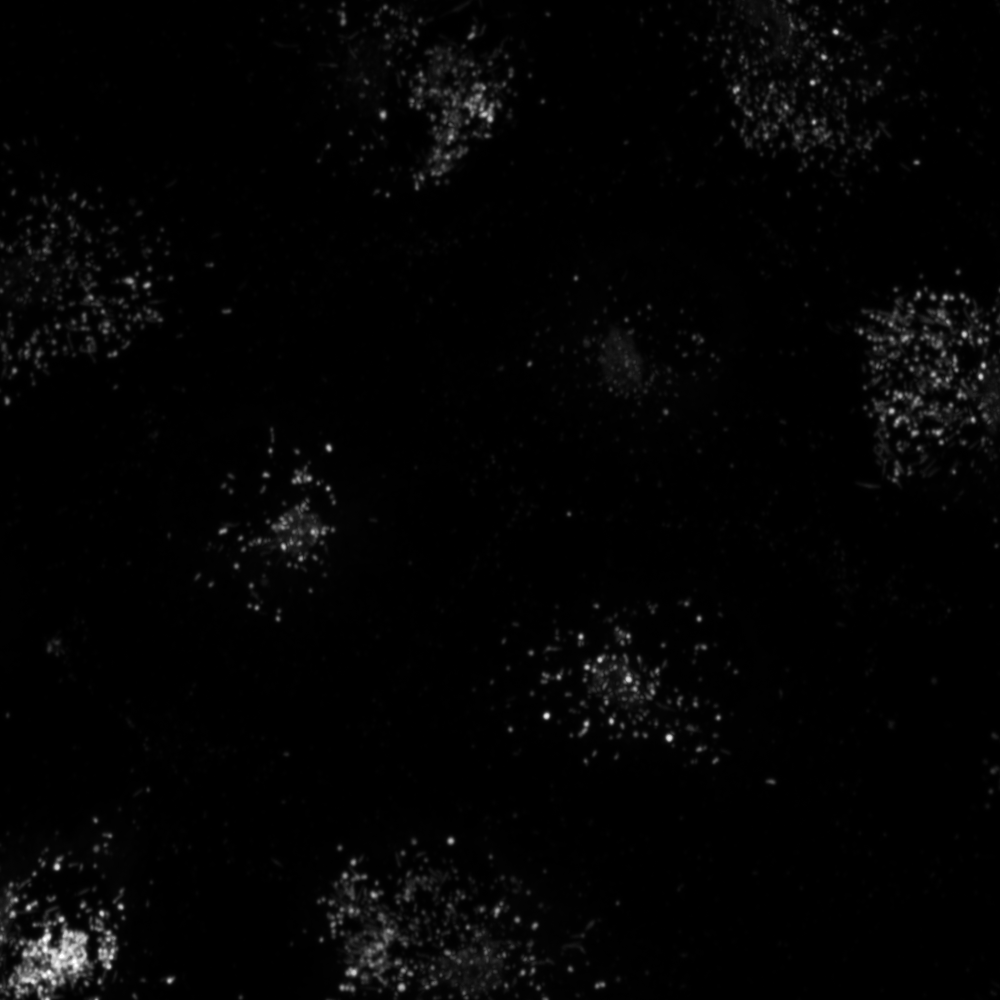

Supplement: Supplementary file 22 — Source data Fig. 7 [file 44318_2024_129_MOESM22_ESM.zip › Figure 7/7C/Figure 7C-RAB8 WT.tif]

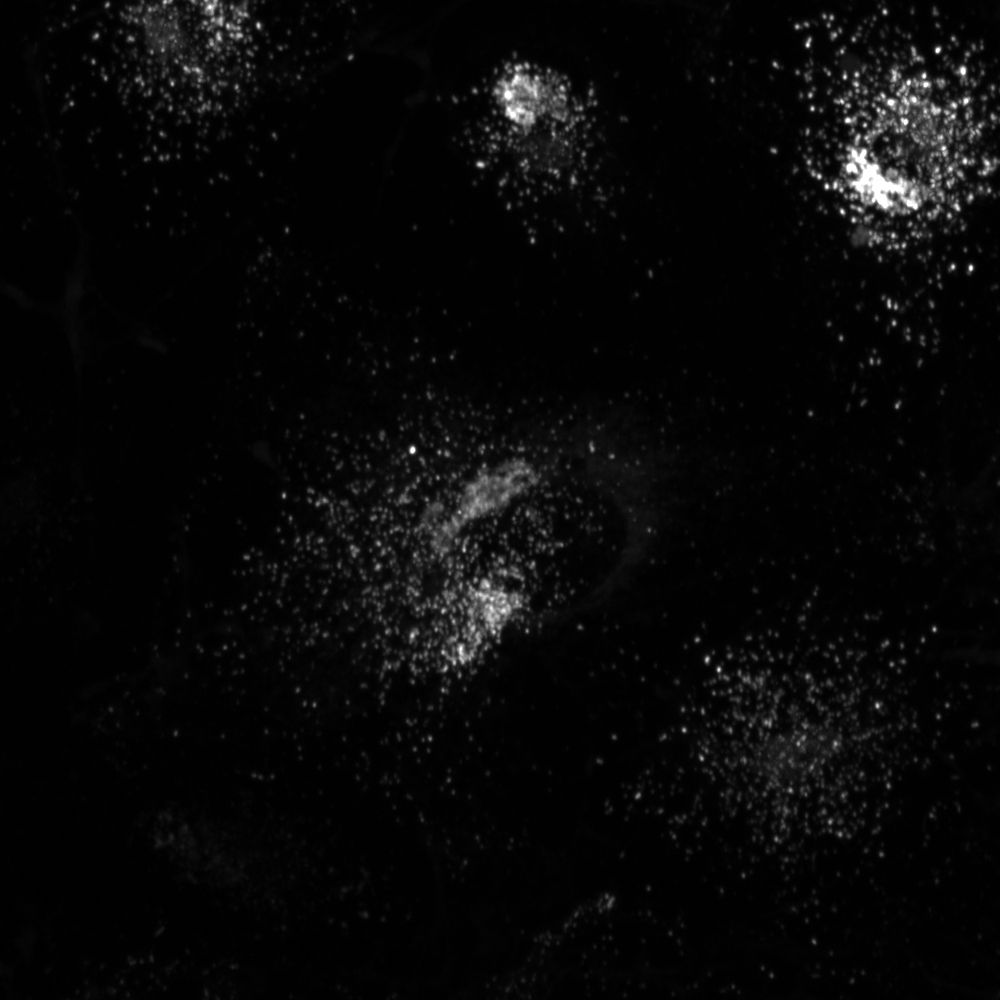

Supplement: Supplementary file 22 — Source data Fig. 7 [file 44318_2024_129_MOESM22_ESM.zip › Figure 7/7C/Figure 7C-EGFP.tif]

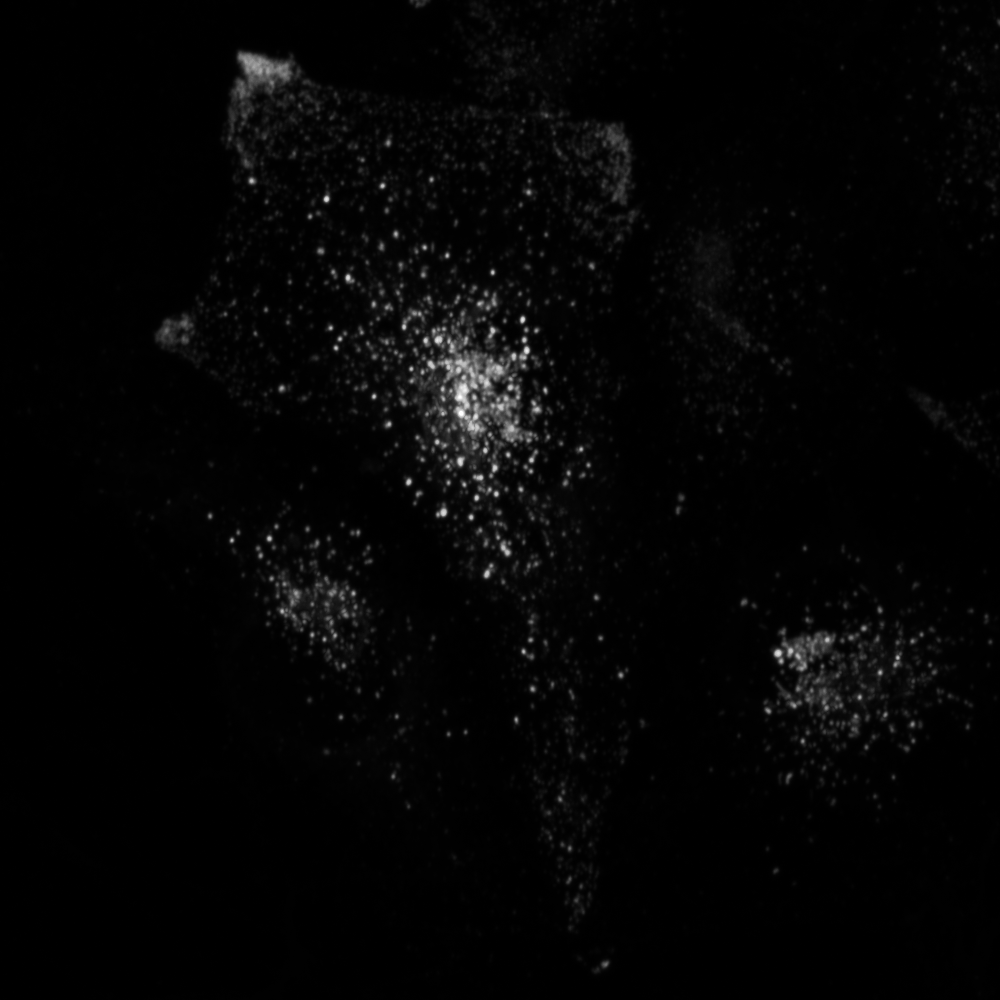

Supplement: Supplementary file 22 — Source data Fig. 7 [file 44318_2024_129_MOESM22_ESM.zip › Figure 7/7C/Figure 7C-RAB8 DN.tif]

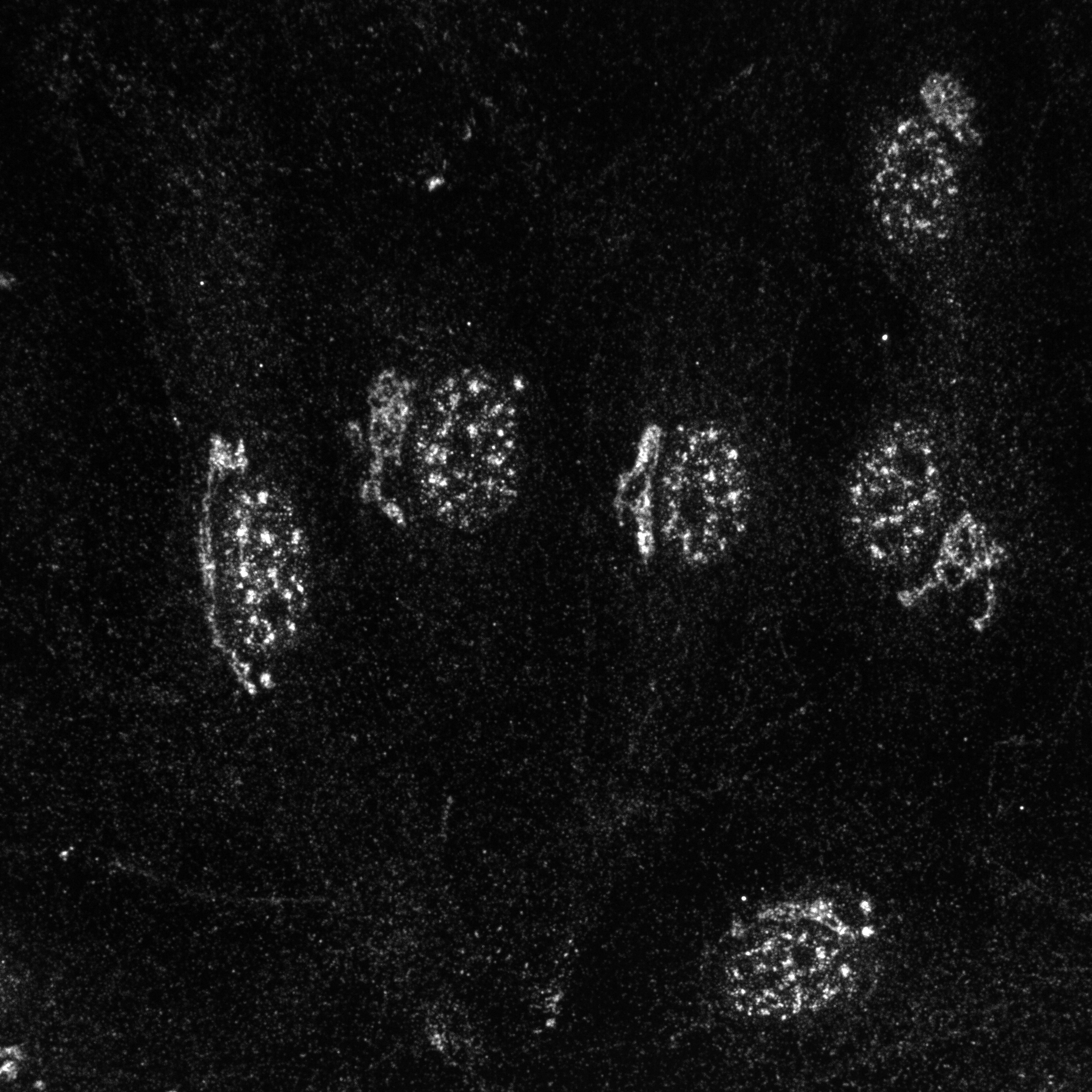

Supplement: Supplementary file 22 — Source data Fig. 7 [file 44318_2024_129_MOESM22_ESM.zip › Figure 7/7G/Figure 7G-EGFP only.tif]

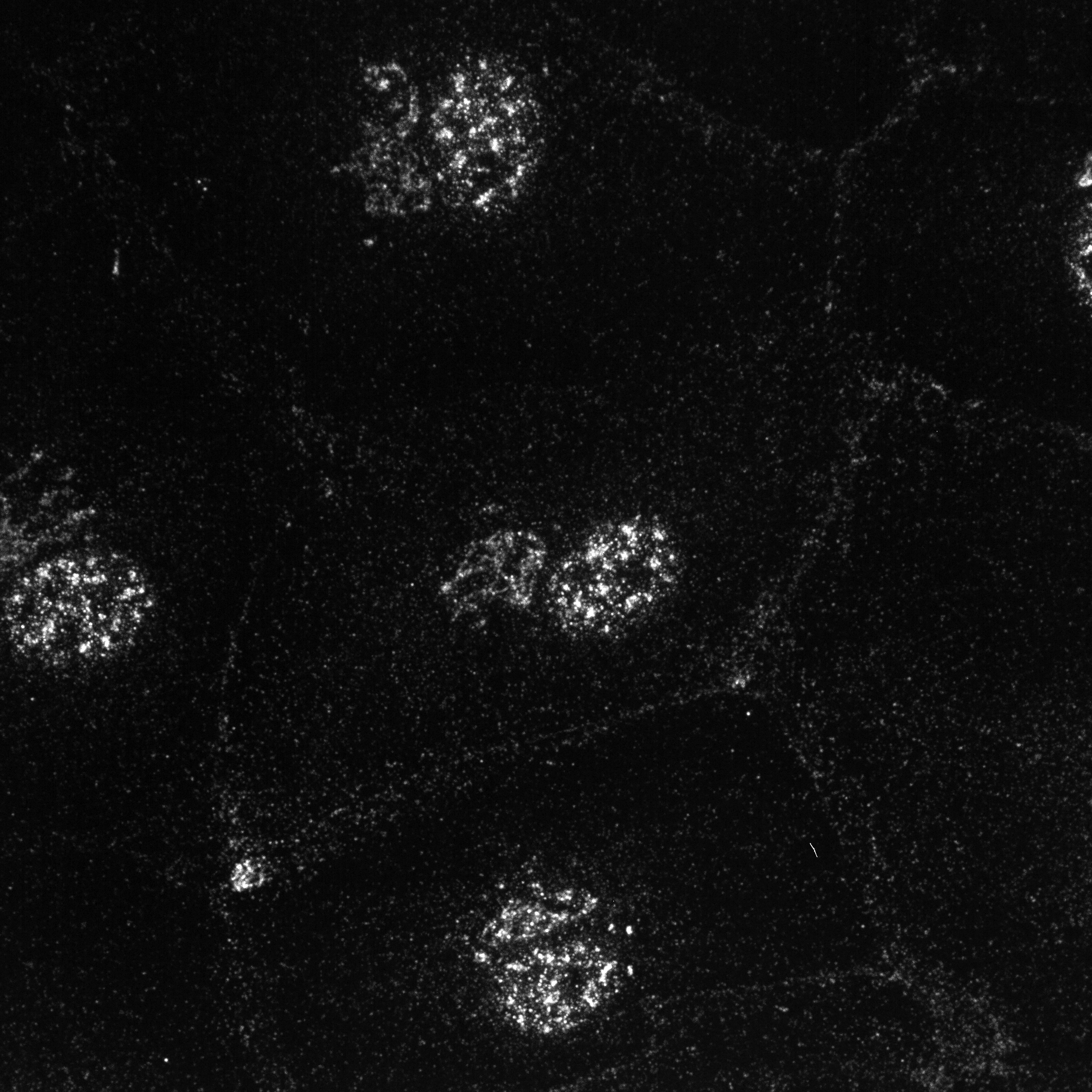

Supplement: Supplementary file 22 — Source data Fig. 7 [file 44318_2024_129_MOESM22_ESM.zip › Figure 7/7G/Figure 7G-RAB8DN.tif]

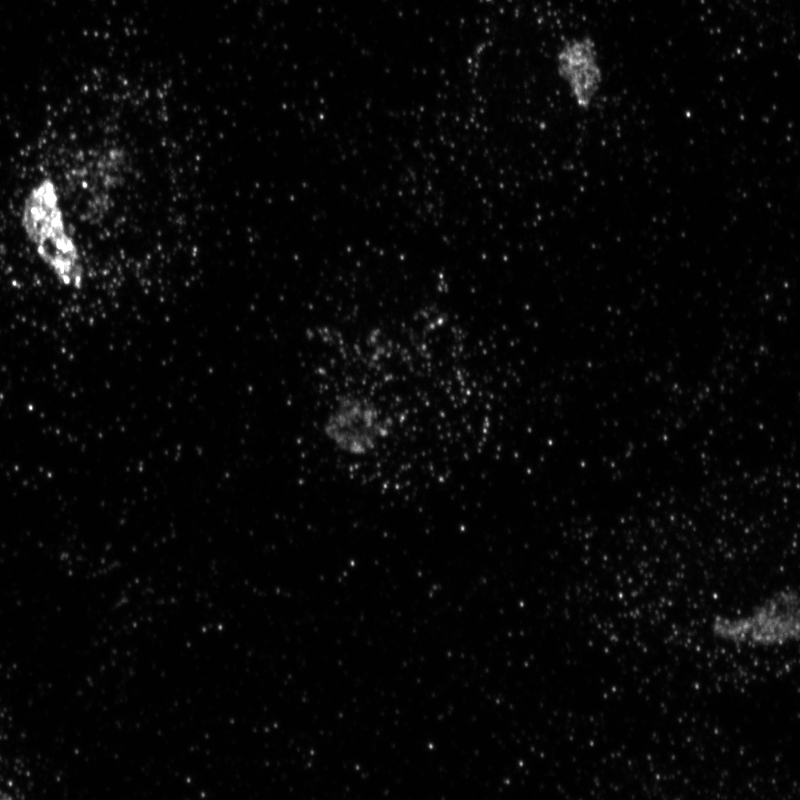

Supplement: Supplementary file 22 — Source data Fig. 7 [file 44318_2024_129_MOESM22_ESM.zip › Figure 7/7H/Figure 7H-EGFP.tif]

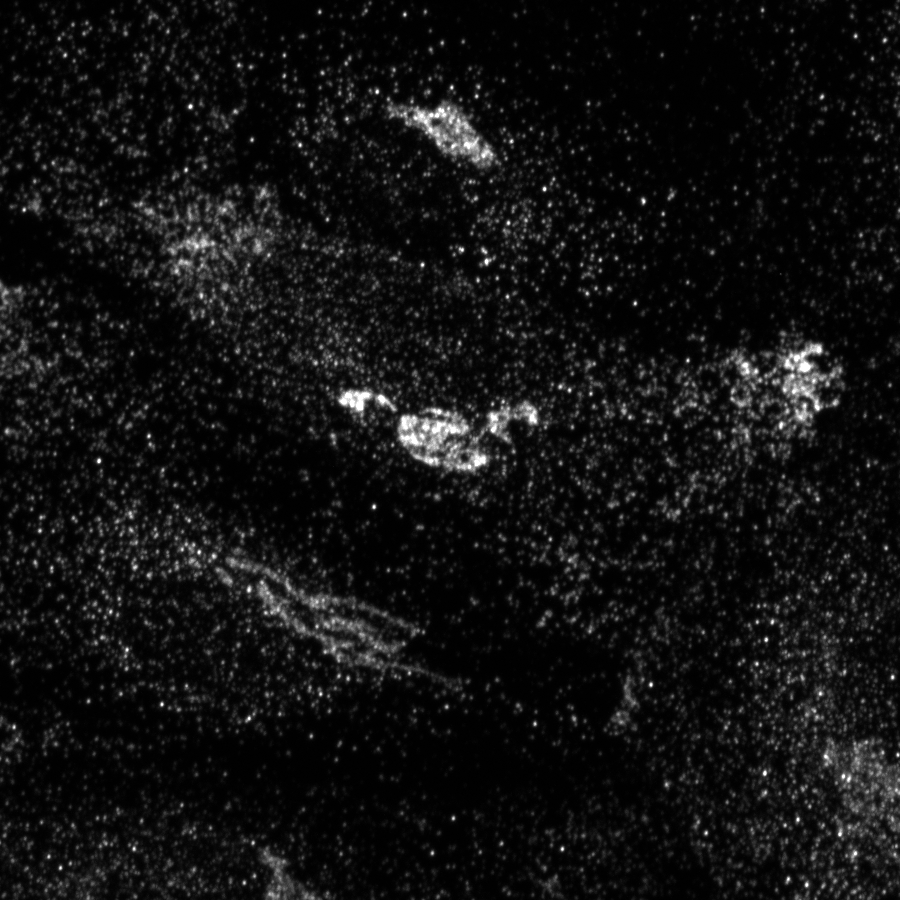

Supplement: Supplementary file 22 — Source data Fig. 7 [file 44318_2024_129_MOESM22_ESM.zip › Figure 7/7H/Figure 7H-RAB8 DN.tif]

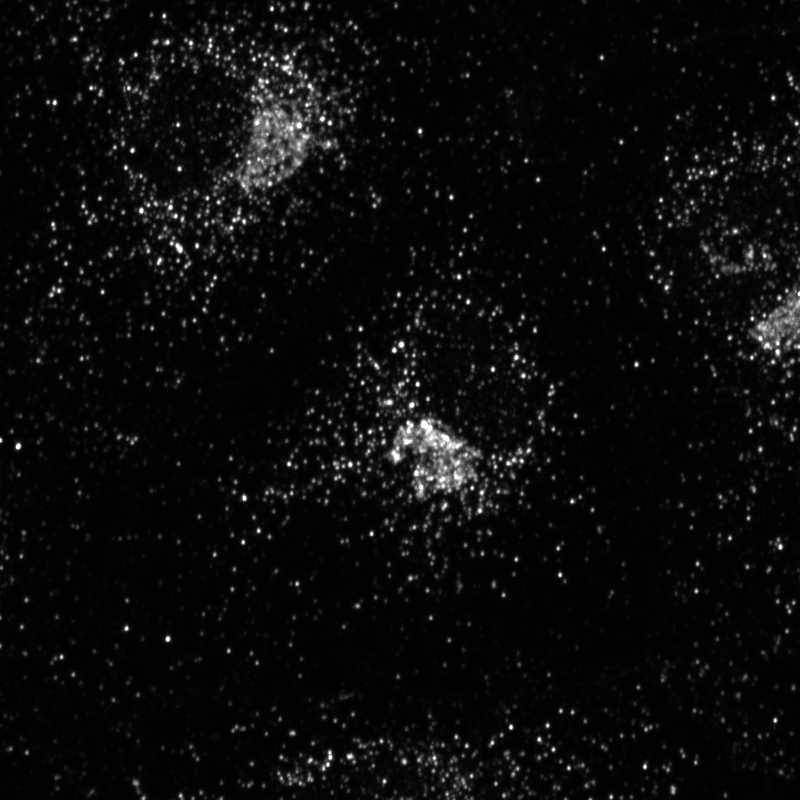

Supplement: Supplementary file 22 — Source data Fig. 7 [file 44318_2024_129_MOESM22_ESM.zip › Figure 7/7H/Figure 7H-RAB8 WT.tif]
