# Supplementary material for: Spatially targeted chemokine exocytosis guides transmigration at lymphatic endothelial multicellular junctions
Source: EMBO J. 2024 Jun 14;43(15):4. doi: 10.1038/s44318-024-00129-x (PMC11294460; doi:10.1038/s44318-024-00129-x)
Supplement: Supplementary file 21 — Source data Fig. 6 [file 44318_2024_129_MOESM21_ESM.zip › Figure 6/6C/READ ME.rtf]

Channel 1 = VE-cadherinChannel 2 = CCL21dc-mCherryChannel 3 = RAB6Channel 4 = DAPI
