# Supplementary material for: Spatially targeted chemokine exocytosis guides transmigration at lymphatic endothelial multicellular junctions
Source: EMBO J. 2024 Jun 14;43(15):4. doi: 10.1038/s44318-024-00129-x (PMC11294460; doi:10.1038/s44318-024-00129-x)
Supplement: Supplementary file 24 — Expanded View Figures [file 44318_2024_129_MOESM24_ESM.pdf]

Expanded View Figures

Figure EV1. (i) Anchoring incapable CCL21ΔC-mCherry supports DC transmigration at multicellular junctions and (ii) CCR7$^{-/-}$ are phenotypically normal.

(**A**) VE-cadherin (red) and LYVE1 (green) staining of mouse ear pinna dermis. A flattened overview image and zoom-in of a single optical slice are shown. The images represent $n = 3$ mice. (**B**) The dot plot shows mean ± SD percentage of wild-type or CCR7$^{-/-}$ DCs that detached, subsequent to the arrest at multicellular junction. The data point represents $n = 6$ wild-type and $n = 5$ CCR7$^{-/-}$ biological replicates from three independent experiments, altogether, representing 401 wild-type and 140 CCR7$^{-/-}$ DCs. The data is related to Fig. 1E–G. (**C**) Schematic shows mCherry tagged full-length CCL21 and CCL21ΔC-mCherry that lacks the charged C-terminus (Hirose et al, 2002).
(**D**) Quantification of wild-type DC transmigration sites in CCL21ΔC-mCherry expressing LEC cultures. The stacked bar graph shows transmigration sites as a percentage of all events from 15 biological replicates in three independent experiments and, altogether, $n = 128$ transmigration events. (**E–I**) The bar graphs show the mean percentage ± SD of DCs positive for (**E**) CD86, (**F**) CD11b, (**G**) CD11c, and (**H**) MHCII in wild-type versus CCR7$^{-/-}$ DCs. In (**I**) the bar graph shows mean cell size ± SD of wild-type vs CCR7$^{-/-}$ DCs normalized to the average of wild-type DCs, which was set at 1, in each experiment. In (**E, I**) The data points represent $n = 4$ biological replicates/genotype in two independent experiments. The number of analyzed DC singlets was at least 277000/sample. (**J**) A capture of a phase contrast/immunofluorescence microscopy showing wild-type (magenta) and CCR7$^{-/-}$ DC (green), on a LEC monolayer. The images represent $n = 3$ biological replicates. (**K, L**) Western blot panel of non-muscle myosin heavy chain 2 A (NMH-IIA), actin, and HSC70 (for loading control) in wild-type versus CCR7$^{-/-}$ DCs. Quantification of the band intensities is shown in the western blot data shown in (**L**). The bar graph in (**L**) shows, mean intensity ± SD. Data was normalized to the average of wild-type DCs, which was set at 1. The data in (**K, L**) represent $n = 4$ biological replicates/ genotype and two independent experiments. (**M**) Quantification of human CCL21 mRNA levels in human specific siCCL21 transfected and mouse CCL21ΔC-mCherry expressing LECs. The dot plot shows the mean hCCL21 mRNA level ± SD normalized to the average of siControl samples, which was set at 1 (green line), in each experiment. Data points represent $n = 4$ biological replicates/ siRNA oligo in 2 independent experiments. *P*-values show the comparison to controls. (**N**) Images show mouse CCL21ΔC-mCherry expression in siControl or human CCL21-specific siCCL21 oligo transfection. Images represent $n = 4$ biological replicates in 2 independent experiments. (**O**) Quantification of transmigration sites of wild-type DCs in siControl or siCCL21 transfected and CCL21-mCherry or CCL21ΔC-mCherry expressing LEC cultures. Transmigration sites are shown as a percentage of all events from 2 independent experiments, consisting of siCTRL01 $n = 209$ transmigration events (3 biological replicates), siCTRL02 $n = 187$ (3 biological replicates), siCCL21-05 $n = 137$ (3 biological replicates), or siCCL21-08 $n = 249$ (4 biological replicates) transfected and CCL21-mCherry expressing LEC cultures and of siCTRL01 $n = 96$ (4 biological replicates), siCTRL02 $n = 68$ (4 biological replicates), siCCL21-05 $n = 69$ (4 biological replicates), or siCCL21-08 $n = 223$ (8 biological replicates) transfected and CCL21ΔC-mCherry expressing LEC cultures. (**P**) The bar graph shows a mean number of observed CCL21ΔC-mCherry exocytosis events/cell ± SD in control or BAPTA-AM treated LECs. The data points represent $n = 37$ DMSO control and $n = 33$ BAPTA-AM treated cells in 11 biological replicates across two independent experiments. Data information: In (**A**), yellow arrows indicate the site of zoom-in image and white arrowheads indicate the multicellular junctions. In (**J**), the white arrowheads indicate wild-type and yellow arrows CCR7$^{-/-}$ DC dendrites. The *p*-values in (**B**), (**E–I**), and (**M**) were calculated using a parametric T-test with Welch's correction, whereas in (**L**) and (**P**) *p*-values were calculated using the Mann–Whitney test. In (**O**), Chi-square test was used to test the significance of data. The scale bar is 20 μm in overview images (**A** and **J**), 5 μm in zoom-in images (**A**), and 50 μm in (**N**).

© The Author(s)

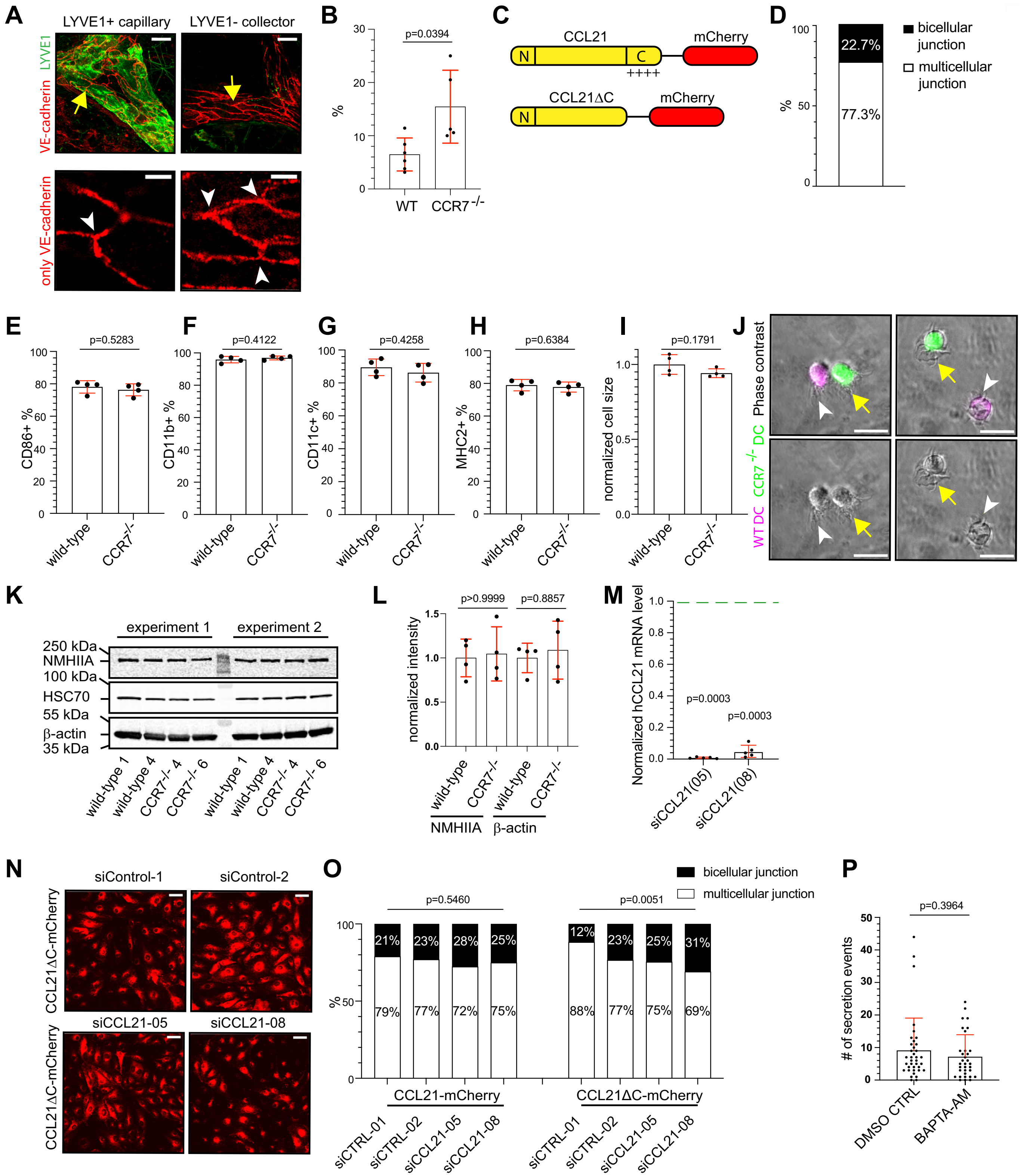
A
LYVE1+ capillary
LYVE1- collector
LYVE1
VE-cadherin
only VE-cadherin
B
p=0.0394
WT
CCR7-/-
C
CCL21
mCherry
N
C
++++
CCL21ΔC
D
bicellular junction
multicellular junction
22.7%
77.3%
E
p=0.5283
CD86+ %
wild-type
CCR7-/-
F
p=0.4122
CD11b+ %
G
p=0.4258
CD11c+ %
H
p=0.6384
MHC2+ %
I
p=0.1791
normalized cell size
J
Phase contrast
WT DC
CCR7-/- DC
K
experiment 1
experiment 2
250 kDa
NMHIIA
100 kDa
HSC70
55 kDa
β-actin
35 kDa
wild-type 1
wild-type 4
CCR7-/- 4
CCR7-/- 6
L
p>0.9999
p=0.8857
normalized intensity
NMHIIA
β-actin
M
Normalized hCCL21 mRNA level
p=0.0003
siCCL21(05)
siCCL21(08)
N
siControl-1
siControl-2
siCCL21-05
siCCL21-08
CCL21ΔC-mCherry
O
p=0.5460
p=0.0051
21%
23%
28%
25%
12%
23%
25%
31%
79%
77%
72%
75%
88%
77%
75%
69%
CCL21-mCherry
CCL21ΔC-mCherry
siCTRL-01
siCTRL-02
siCCL21-05
siCCL21-08
P
p=0.3964
# of secretion events
DMSO CTRL
BAPTA-AM

 © The Author(s)

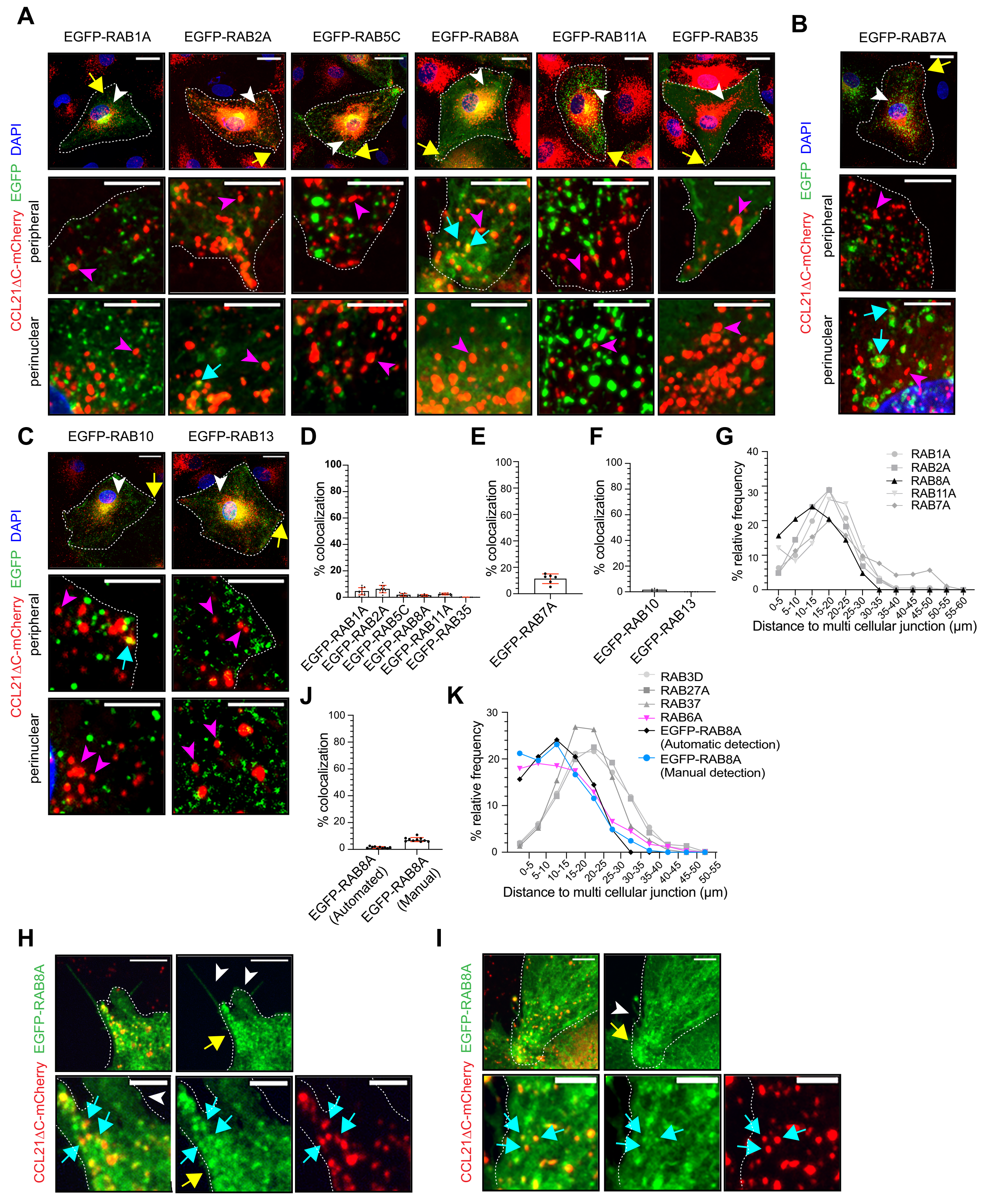
A
EGFP-RAB1A
EGFP-RAB2A
EGFP-RAB5C
EGFP-RAB8A
EGFP-RAB11A
EGFP-RAB35
CCL21ΔC-mCherry EGFP DAPI
peripheral
perinuclear
B
EGFP-RAB7A
C
EGFP-RAB10
EGFP-RAB13
D
% colocalization
EGFP-RAB1A
EGFP-RAB2A
EGFP-RAB5C
EGFP-RAB8A
EGFP-RAB11A
EGFP-RAB35
E
EGFP-RAB7A
F
EGFP-RAB10
EGFP-RAB13
G
% relative frequency
RAB1A
RAB2A
RAB8A
RAB11A
RAB7A
Distance to multi cellular junction (µm)
J
EGFP-RAB8A (Automated)
EGFP-RAB8A (Manual)
K
RAB3D
RAB27A
RAB37
RAB6A
EGFP-RAB8A (Automatic detection)
EGFP-RAB8A (Manual detection)
H
I
CCL21ΔC-mCherry EGFP-RAB8A

© The Author(s)

Figure EV2. Colocalization of CCL21ΔC-mCherry with EGFP-RAB GTPases.

(**A–G**) Immunofluorescence images in (**A–C**) show LECs expressing chemokine CCL21ΔC-mCherry (red) and the indicated EGFP-tagged RAB-GTPase (green). The nuclei were stained with DAPI (blue). In (**A**) images represent $n = 3$ biological replicates from 3 independent experiments and in (**B**, **C**) $n = 2$ biological replicates in 2 experiments. (**D–G**) Quantification of CCL21ΔC-mCherry+ vesicle colocalization with the indicated EGFP-RAB GTPases in the whole LEC area. The dot plots in (**D–F**), show the mean percentage ± SD. Each data point represents a single analyzed cell. In (**A** and **D**) $n = 11$ in EGFP-RAB1A, $n = 10$ in EGFP-RAB2A, $n = 9$ in EGFP-RAB5C, $n = 10$ in EGFP-RAB8A, $n = 9$ in EGFP-RAB11A, $n = 9$ in EGFP-RAB35, all representing three independent experiments. In (**B**, **C**, **E**, and **F**) $n = 6$ in EGFP-RAB7, EGFP-RAB10, and EGFP-RAB13 all representing two independent experiments. (**G**) The histogram shows the distribution (mean percentage) of CCL21ΔC-mCherry and the indicated EGFP-RAB-GTPase colocalized vesicles as a function of distance from a multicellular junction. The number of cells and independent experiments is the same as in (**A–E**). (**H–K**) Immunofluorescence images of LECs expressing EGFP-RAB8A and CCL21ΔC-mCherry. Quantification of colocalization in the whole LEC area is shown in (**J**). The dot plot shows the mean percentage ± SD. Each data point represents a single analyzed cell. $n = 10$ cells, from three independent experiments. (**K**) The histogram shows the distribution (mean percentage) of CCL21ΔC-mCherry and EGFP-RAB8A colocalized vesicles, as a function of distance from a multicellular junction and is projected on the histogram shown in Fig. 2F. The number of samples represented is the same as in (**J**). Data information: In (**A–C**), yellow arrows and white arrowheads indicate the site of the peripheral and perinuclear areas, respectively, shown in the zoom-in images. In the zoom-in images, cyan arrows indicate the colocalizing, and the magenta arrowheads examples of the more abundant non-colocalizing CCL21ΔC-mCherry+ vesicles. In (**H**, **I**), white arrowheads indicate plasma membrane localization of the EGFP-RAB8A and yellow arrows high-intensity areas in the vicinity of multicellular junctions. The cyan arrows highlight examples of colocalizing vesicles that were missed in the semi-automated analysis but included in the manual detection. In (**A–C**) and (**H**, **I**) cell borders are indicated with white dotted lines. Scale bars are 20 µm in overview images; 5 µm in zoom-in images in (**A**, **C** and **H**, **I**), and 3 µm in (**B**).

 © The Author(s)

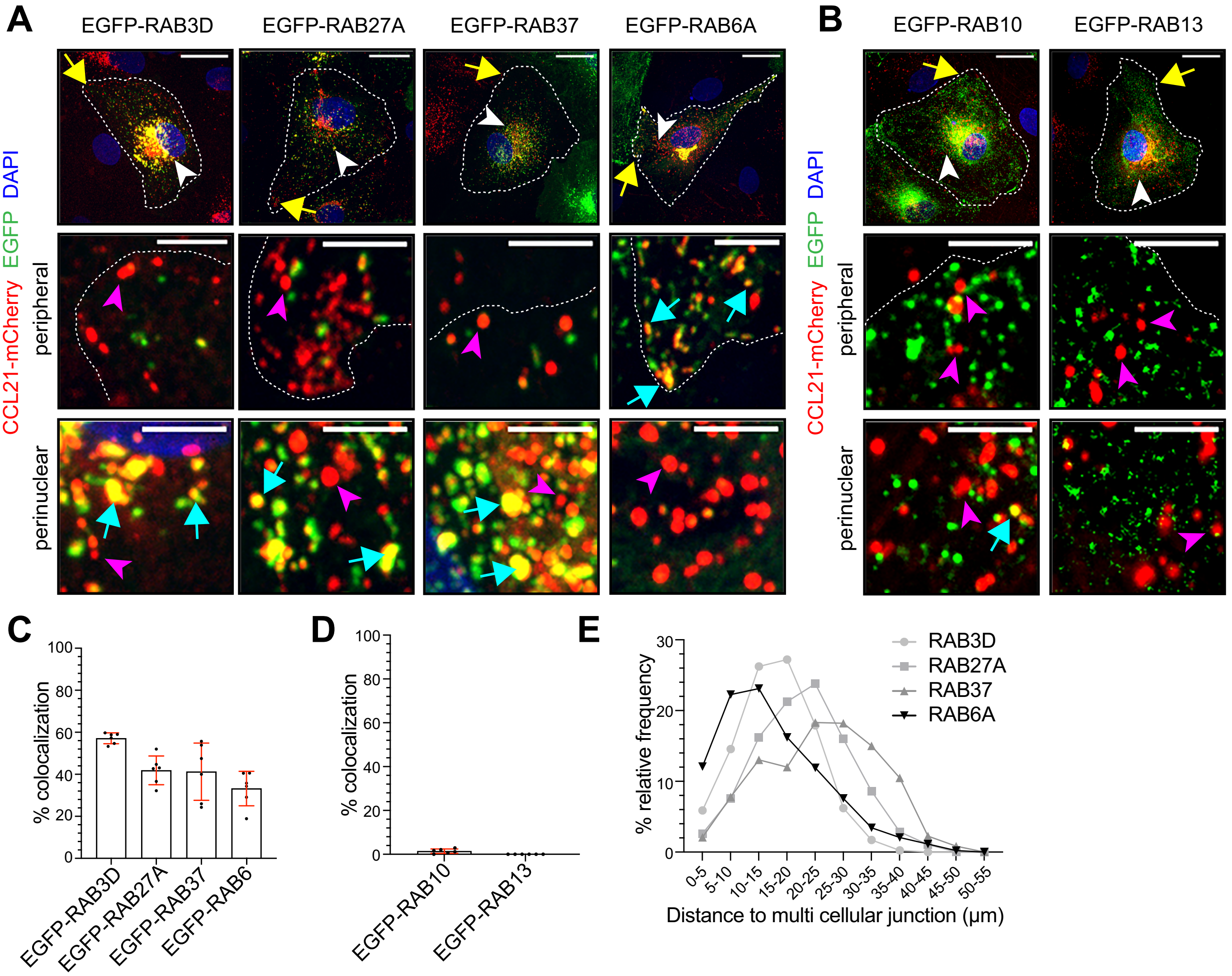

Figure EV3. Colocalization of CCL21-mCherry with EGFP-RAB GTPases.

(**A–E**) Shows colocalization of full-length CCL21-mCherry (red) with the indicated EGFP-tagged RAB-GTPase (green). The nuclei were stained with DAPI (blue). (**C, D**) Quantification of CCL21-mCherry+ vesicle colocalization with the indicated EGFP-RAB GTPases in the whole LEC area. The dot plots show the mean percentage ± SD. Each data point represents a single analyzed cell with $n = 6$ cells for each of EGFP-RAB3D, EGFP-RAB27A, EGFP-RAB37, EGFP-RAB6, EGFP-RAB10, and EGFP-RAB13 representing two independent experiments. (**E**) The histogram shows the distribution (mean percentage) of CCL21ΔC-mCherry and the indicated EGFP-RAB-GTPase colocalized vesicles as a function of distance from a multicellular junction. The number of cells and independent experiments is the same as in (**C**). Data information: In (**A, B**), the perinuclear and peripheral areas (shown in the zoom-in images below the overviews) are indicated with white arrowheads and yellow arrows, respectively. Colocalization is indicated with cyan arrows and non-colocalizing CCL21-mCherry vesicles are indicated with magenta arrowheads. The cell borders are indicated with white dotted lines. Scale bars are 20 μm in the overview images; 3 μm in zoom-in images.

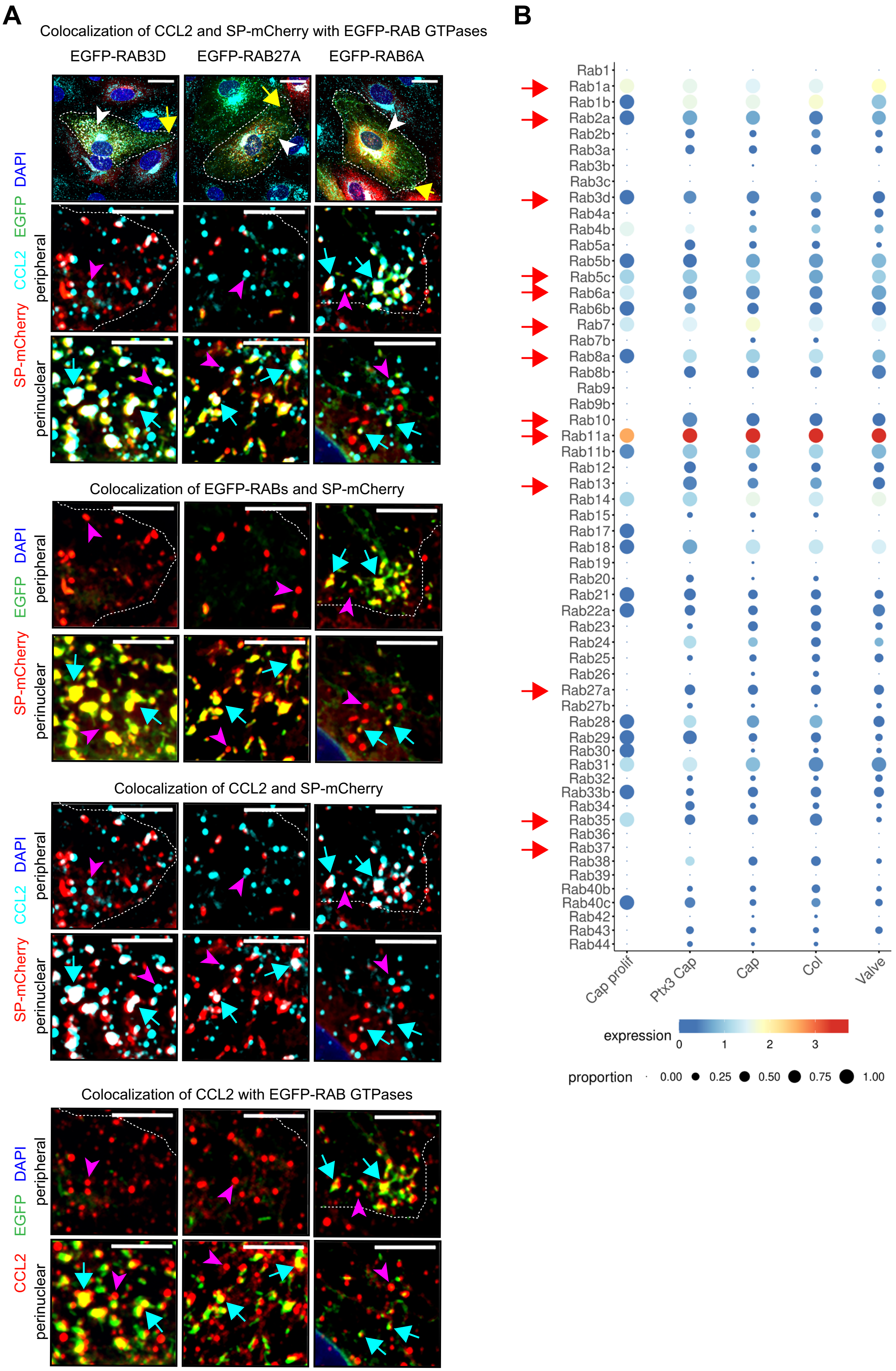
A
Colocalization of CCL2 and SP-mCherry with EGFP-RAB GTPases
EGFP-RAB3D
EGFP-RAB27A
EGFP-RAB6A
DAPI EGFP CCL2 SP-mCherry
peripheral
perinuclear
Colocalization of EGFP-RABs and SP-mCherry
DAPI EGFP SP-mCherry
Colocalization of CCL2 and SP-mCherry
DAPI CCL2 SP-mCherry
Colocalization of CCL2 with EGFP-RAB GTPases
DAPI EGFP CCL2
B
Rab1
Rab1a
Rab1b
Rab2a
Rab2b
Rab3a
Rab3b
Rab3c
Rab3d
Rab4a
Rab4b
Rab5a
Rab5b
Rab5c
Rab6a
Rab6b
Rab7
Rab7b
Rab8a
Rab8b
Rab9
Rab9b
Rab10
Rab11a
Rab11b
Rab12
Rab13
Rab14
Rab15
Rab17
Rab18
Rab19
Rab20
Rab21
Rab22a
Rab23
Rab24
Rab25
Rab26
Rab27a
Rab27b
Rab28
Rab29
Rab30
Rab31
Rab32
Rab33b
Rab34
Rab35
Rab36
Rab37
Rab38
Rab39
Rab40b
Rab40c
Rab42
Rab43
Rab44
Cap prolif
Ptx3 Cap
Cap
Col
Valve
expression
0
1
2
3
proportion
0.00
0.25
0.50
0.75
1.00

 © The Author(s)

Figure EV4. (i) Colocalization of chemokine CCL2 and general secretory marker SP-mCherry with EGFP-RAB-GTPases, (ii) expression levels of RAB-GTPases in dermal LECs.

(**A**) TNF-α treated LECs expressing signal peptide tagged with mCherry (SP-mCherry; red) together with the indicated EGFP-RAB GTPases (green) and stained for endogenous CCL2 (cyan) and nuclei (DAPI, blue). The overview images show a merge of CCL2, SP-mCherry, and EGFP-RAB signals. The top panel shows a merge of all three channels and the lower panels show a merge of the indicated 2 channels. The images represent $n = 3$ independent experiments. (**B**) Re-analyses of single-cell mRNA sequencing data shows RAB-GTPase expression in mouse dermal LECs in vivo. Cap prolif = proliferative capillary LECs, Ptx3 Cap = Ptx3+ capillary LECs, Cap = capillary LECs, Col = collector LECs, and Valve = valve LECs. The size of the dots indicates the proportion of LECs expressing the indicated RAB-GTPases and the color indicates the level of expression. The data was compiled using the database interface published at https://makinenlab.shinyapps.io/DermaLymphaticEndothelialCells/, which is related to a recent study reported by Petkova et al (data ref: Gene Expression Omnibus, GSE201916) (Petkova et al, 2023). The RAB-GTPases used in this study are indicated with red arrows. Data information: In (**A**), yellow arrows and white arrowheads indicate the site of the peripheral and perinuclear areas, respectively, shown in the zoom-in images. In the zoom-in images, examples of triple positive vesicles (SP-mCherry, EGFP-RAB, and CCL2) are shown with cyan arrows. Magenta arrowheads indicate non-colocalizing CCL2+ or SP-mCherry vesicles. Cell borders are indicated with white dotted lines. Scale bars, 20 µm in overview images; 5 µm in zoom-in images.

© The Author(s)

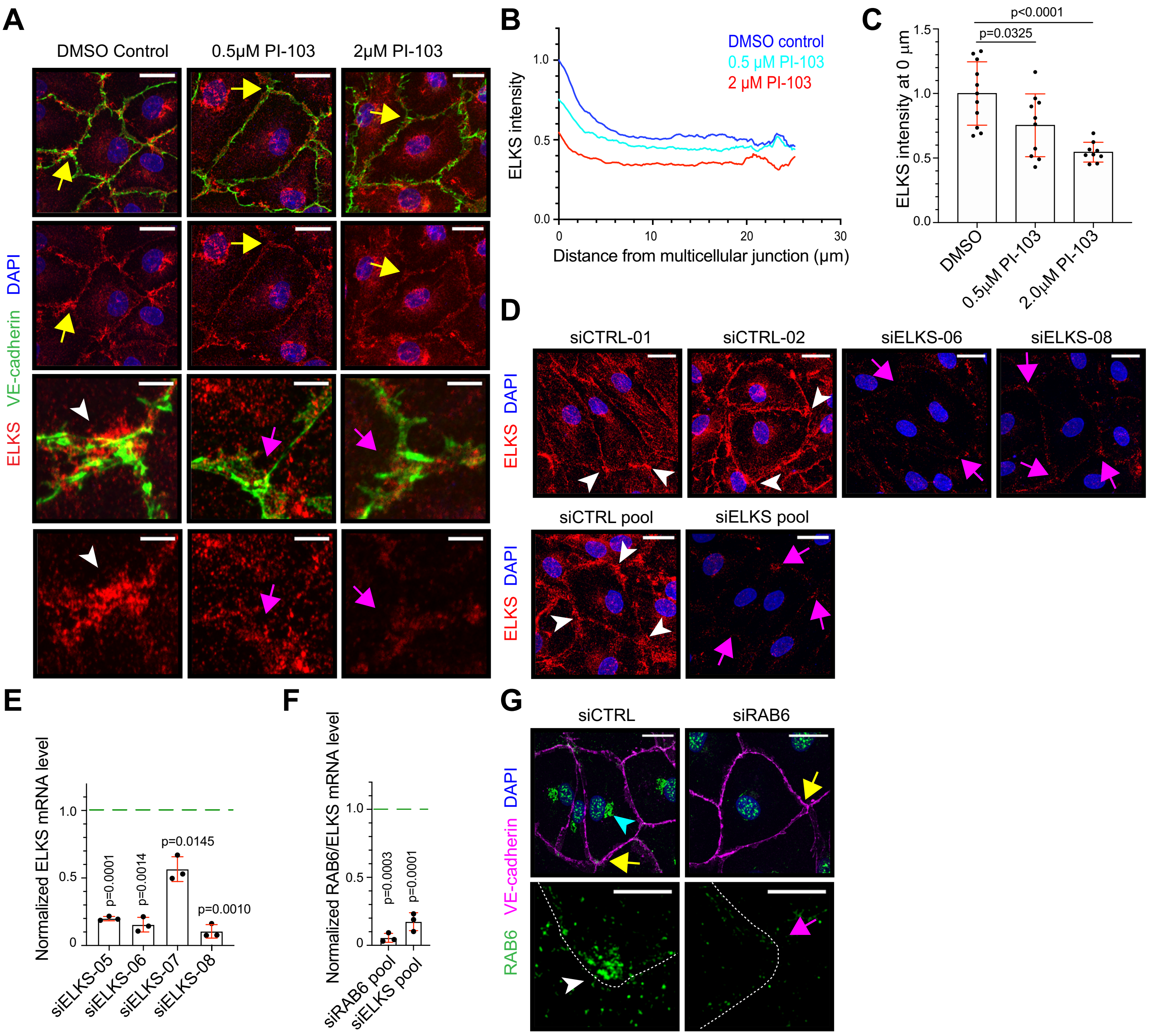

Figure EV5. (i) Inhibition of the PI3K attenuates ELKS localization at the LEC multicellular junctions, and (ii) confirmation of the silencing efficiency of the used siRNA oligos.

(A–C) LEC monolayer was treated with DMSO (control), 0.5 μM P1-103, or 2 μM PI-103 for 1 h. Fixed samples were stained for ELKS (red), VE-cadherin (green), and nuclei (DAPI, blue). **(B, C)** Quantification of ELKS staining at the LEC junctions in control (DMSO) or 0.5 μM, or 2 μM PI-103 treated LEC monolayers. The graph in **(B)** shows mean ELKS intensity as a function of distance from the nearest multicellular junction. In **(C)**, the dot plot shows mean ELKS intensity ± SD at the multicellular junction (distance 0 μm). The results were normalized to the average of controls (set at 1) in each experiment. In **(A–C)**, the data represent $n = 11$ (DMSO), $n = 10$ (0.5 μM PI-103), and $n = 9$ (2 μM PI-103) biological replicates, representing, altogether, 399 (DMSO control), 318 (0.5 μM PI-103), and 286 (2 μM PI-103) junctions in 5 independent experiments. **(D)** Images show the effect of siRNA-mediated knockdown of ELKS. Monolayers were stained for ELKS (red) and nuclei (DAPI, blue). Images are representative of at least $n = 3$ independent experiments. **(E, F)** Quantification of ELKS and RAB6 mRNA levels upon siELKS or siRAB6 treatments, respectively. The dot plots show ELKS or RAB6 mean mRNA level ± SD normalized to the average of siControl samples (set at 1, green dashed line) in each experiment. *P*-values show the comparison to controls. Data points represent $n = 3$ independent experiments derived, altogether, from 6 (siELKS-05), 8 (siELKS06), 6 (siELKS-07), 8 (siELKS08), 12 (siControl 01/02), 7 (siELKS pool), 5 (siRAB6 pool), or 12 (siControl pool) biological replicates. **(G)** Images show the effect of siRAB6 on RAB6 protein level. LEC monolayer was treated with the indicated oligos and stained for RAB6 (green), VE-cadherin (magenta), and nuclei (DAPI, blue). Images are representative of at least $n = 3$ independent experiments. Data information: In **(A)** and **(G)**, yellow arrows indicate the site of the zoom-in images shown below. In **(A)**, **(D)**, and **(G)** white arrowheads indicate accumulation at the multicellular junctions and the magenta arrows lack of accumulation. In **(G)**, the cyan arrow in the siCTRL-treated sample shows RAB6 signal in the Golgi apparatus, which is not seen upon knock down in siRAB6 treated sample. In **(G)**, LEC borders are marked with white dotted line. The *p*-values in **(C)** and **(E, F)** were calculated using parametric t-test with Welch's correction. Scale bars, 20 μm in overview images; 5 μm in zoom-in images.

 © The Author(s)
